# The Streamlined Genome of *Phytomonas* spp. Relative to Human Pathogenic Kinetoplastids Reveals a Parasite Tailored for Plants

**DOI:** 10.1371/journal.pgen.1004007

**Published:** 2014-02-06

**Authors:** Betina M. Porcel, France Denoeud, Fred Opperdoes, Benjamin Noel, Mohammed-Amine Madoui, Tansy C. Hammarton, Mark C. Field, Corinne Da Silva, Arnaud Couloux, Julie Poulain, Michael Katinka, Kamel Jabbari, Jean-Marc Aury, David A. Campbell, Roxana Cintron, Nicholas J. Dickens, Roberto Docampo, Nancy R. Sturm, V. Lila Koumandou, Sandrine Fabre, Pavel Flegontov, Julius Lukeš, Shulamit Michaeli, Jeremy C. Mottram, Balázs Szöőr, Dan Zilberstein, Frédéric Bringaud, Patrick Wincker, Michel Dollet

**Affiliations:** 1 Commissariat à l'Energie Atomique (CEA), Institut de Génomique (IG), Genoscope, Evry, France; 2 Université d'Evry, UMR 8030, Evry, France; 3 Centre National de Recherche Scientifique (CNRS), UMR 8030, Evry, France; 4 de Duve Institute, Université catholique de Louvain, Brussels, Belgium; 5 Institute of Infection, Immunity and Inflammation, College of Medical, Veterinary and Life Sciences, University of Glasgow, Glasgow, United Kingdom; 6 Department of Pathology, University of Cambridge, Cambridge, United Kingdom; 7 Department of Microbiology, Immunology & Molecular Genetics, David Geffen School of Medicine, University of California at Los Angeles, Los Angeles, California, United States of America; 8 Center for Tropical and Emerging Global Diseases and Department of Cellular Biology, University of Georgia, Athens, Georgia, United States of America; 9 Wellcome Trust Centre for Molecular Parasitology, Institute of Infection, Immunity and Inflammation, College of Medical, Veterinary and Life Sciences, University of Glasgow, Glasgow, United Kingdom; 10 Biomedical Research Foundation, Academy of Athens, Athens, Greece; 11 CIRAD, TA A-98/F, Campus International de Baillarguet, Montpellier, France; 12 Institute of Parasitology, Biology Centre and Faculty of Sciences, University of South Bohemia, České Budějovice (Budweis), Czech Republic; 13 The Mina & Everard Goodman Faculty of Life Sciences, Bar-Ilan University, Ramat-Gan, Israel; 14 Centre for Immunity, Infection and Evolution, Institute of Immunology and Infection Research, School of Biological Sciences, University of Edinburgh, Edinburgh, United Kingdom; 15 Faculty of Biology, Technion-Israel Institute of Technology, Haifa, Israel; 16 Centre de Résonance Magnétique des Systèmes Biologiques, Université Bordeaux Segalen, CNRS UMR-5536, Bordeaux, France; Virginia Tech, United States of America

## Abstract

Members of the family Trypanosomatidae infect many organisms, including animals, plants and humans. Plant-infecting trypanosomes are grouped under the single genus *Phytomonas*, failing to reflect the wide biological and pathological diversity of these protists. While some *Phytomonas* spp. multiply in the latex of plants, or in fruit or seeds without apparent pathogenicity, others colonize the phloem sap and afflict plants of substantial economic value, including the coffee tree, coconut and oil palms. Plant trypanosomes have not been studied extensively at the genome level, a major gap in understanding and controlling pathogenesis. We describe the genome sequences of two plant trypanosomatids, one pathogenic isolate from a Guianan coconut and one non-symptomatic isolate from *Euphorbia* collected in France. Although these parasites have extremely distinct pathogenic impacts, very few genes are unique to either, with the vast majority of genes shared by both isolates. Significantly, both *Phytomona*s spp. genomes consist essentially of single copy genes for the bulk of their metabolic enzymes, whereas other trypanosomatids e.g. *Leishmania* and *Trypanosoma* possess multiple paralogous genes or families. Indeed, comparison with other trypanosomatid genomes revealed a highly streamlined genome, encoding for a minimized metabolic system while conserving the major pathways, and with retention of a full complement of endomembrane organelles, but with no evidence for functional complexity. Identification of the metabolic genes of *Phytomonas* provides opportunities for establishing *in vitro* culturing of these fastidious parasites and new tools for the control of agricultural plant disease.

## Author Summary

Some plant trypanosomes, single-celled organisms living in phloem sap, are responsible for important palm diseases, inducing frequent expensive and toxic insecticide treatments against their insect vectors. Other trypanosomes multiply in latex tubes without detriment to their host. Despite the wide range of behaviors and impacts, these trypanosomes have been rather unceremoniously lumped into a single genus: *Phytomonas*. A battery of molecular probes has been used for their characterization but no clear phylogeny or classification has been established. We have sequenced the genomes of a pathogenic phloem-specific *Phytomonas* from a diseased South American coconut palm and a latex-specific isolate collected from an apparently healthy wild euphorb in the south of France. Upon comparison with each other and with human pathogenic trypanosomes, both *Phytomonas* revealed distinctive compact genomes, consisting essentially of single-copy genes, with the vast majority of genes shared by both isolates irrespective of their effect on the host. A strong cohort of enzymes in the sugar metabolism pathways was consistent with the nutritional environments found in plants. The genetic nuances may reveal the basis for the behavioral differences between these two unique plant parasites, and indicate the direction of our future studies in search of effective treatment of the crop disease parasites.

## Introduction

Flagellated protists of the family Trypanosomatidae, class Kinetoplastea, infect a large variety of organisms including animals, plants and humans [1]. While African and South-American trypanosomes are responsible for sleeping sickness [2] and Chagas' disease [3], respectively, different *Leishmania* spp. cause visceral, cutaneous and mucocutaneous manifestations of leishmaniasis in many tropical and subtropical regions [4].

Various eukaryotes, particularly filamentous microorganisms like oomycetes and fungi have acquired the capacity to infect and grow inside the plant tissues. While some of these organisms could influence plant growth positively, in most cases they can cause major diseases in plants of economic importance [5]. The genomes of numerous of these filamentous plant pathogens have already been sequenced, unveiling an amazing variety of genome sizes and organization [6]. Certainly, a great number of these plant pathogens were molded into larger genomes by repeat-driven expansions, with the genes coding for proteins involved in host interactions located within repeat-rich regions [6]. In contrast, some filamentous plant pathogens have fairly small genomes, as a consequence of intron or gene loss, like *U. maydis*;(21 Mb) [7] and *Albugo laibachii*; (37 Mb) [8], or abridged transposon content as in *Sclerotinia sclerotiorum*; (38 Mb) [9].

Like fungi and oomycetes, trypanosomatids also infect plants, but using a radically different strategy to colonize and propagate inside the host [10]–[12]. Multiple insect species of the order Heteroptera act as the natural vectors of plant trypanosomatids, both in the transmission to lactiferous hosts [10], [13], and for infection by intraphloemic plant trypanosomes [14]–[16]. *Phytomonas* is the arbitrary genus name proposed for all trypanosomatids specific to plants [17]; however this rather restricted taxonomic description fails to fully capture the wide diversity of trypanosomatids encountered in plants, both with respect to their biological properties and their impact on the host [18]–[22]. Indeed, *Phytomonas* spp. infect more than 100 plant species, distributed primarily in tropical and subtropical zones, by multiplying in latex tubes, fruits and seeds or colonizing the phloem sap inside the sieve tubes. *Phytomonas* infection can occur without apparent pathogenicity, but conversely it can cause lethal disease in plants of substantial economic value, including the coffee tree, coconut and oil palms [10], [23]. This results in important economic losses in Latin America and the Caribbean [24]–[27]. Ten distinct subgroups of plant trypanosomatids have been defined using the internal transcribed spacer region of the ribosomal RNA locus [22]. Only Group H, encompassing the Latin American intraphloemic trypanosomatids responsible for severe wilts, can be distinguished both by rRNA markers as well as biological and serological properties [19]. A full definition of the diversity of trypanosomatids within the overarching *Phytomonas* genus is still outstanding.

The whole genome sequences of *Trypanosoma cruzi*, *Trypanosoma brucei* and *Leishmania major* were released in 2005 [28]–[31]. Since then, the genomes of several additional trypanosomatids, including several pathogens of mammals, have been completed and described [32]–[35]. These databases have provided an essential platform for investigations of basic biology and mechanisms of pathogenesis and facilitated the exploration of novel therapies.

However, to date genome level analysis of *Phytomonas* spp. is limited. The biology of these parasites is reasonably well described [11], [36], but little information exists on their effective control by chemicals or, most critically, on their specific adaptations to the plant host and the mechanisms underpinning pathogenesis. Moreover, few genes are available in sequence databases, and little is known about genome size, chromosomal organization and ploidy [37], [38].

We describe here the genome sequences of two plant trypanosomatids, one phloem-restricted pathogenic isolate from a diseased coconut from Guiana (HART1 from Group H) and the other a non-symptomatic latex isolate from *Euphorbia* (EM1 from Group D) [22]. The comparison of these two plant parasite genomes with each other and with those of other trypanosomes reveals a common simplified genome organization for the plant trypanosomes. Identification of the genes involved in *Phytomonas* metabolism is an important step for improving *in vitro* culture protocols and for development of new and better tools for the control and diagnosis of *Phytomonas*-mediated diseases.

## Results and Discussion

### General features of *Phytomonas* EM1 and HART1 genomes

**Figure 1:**
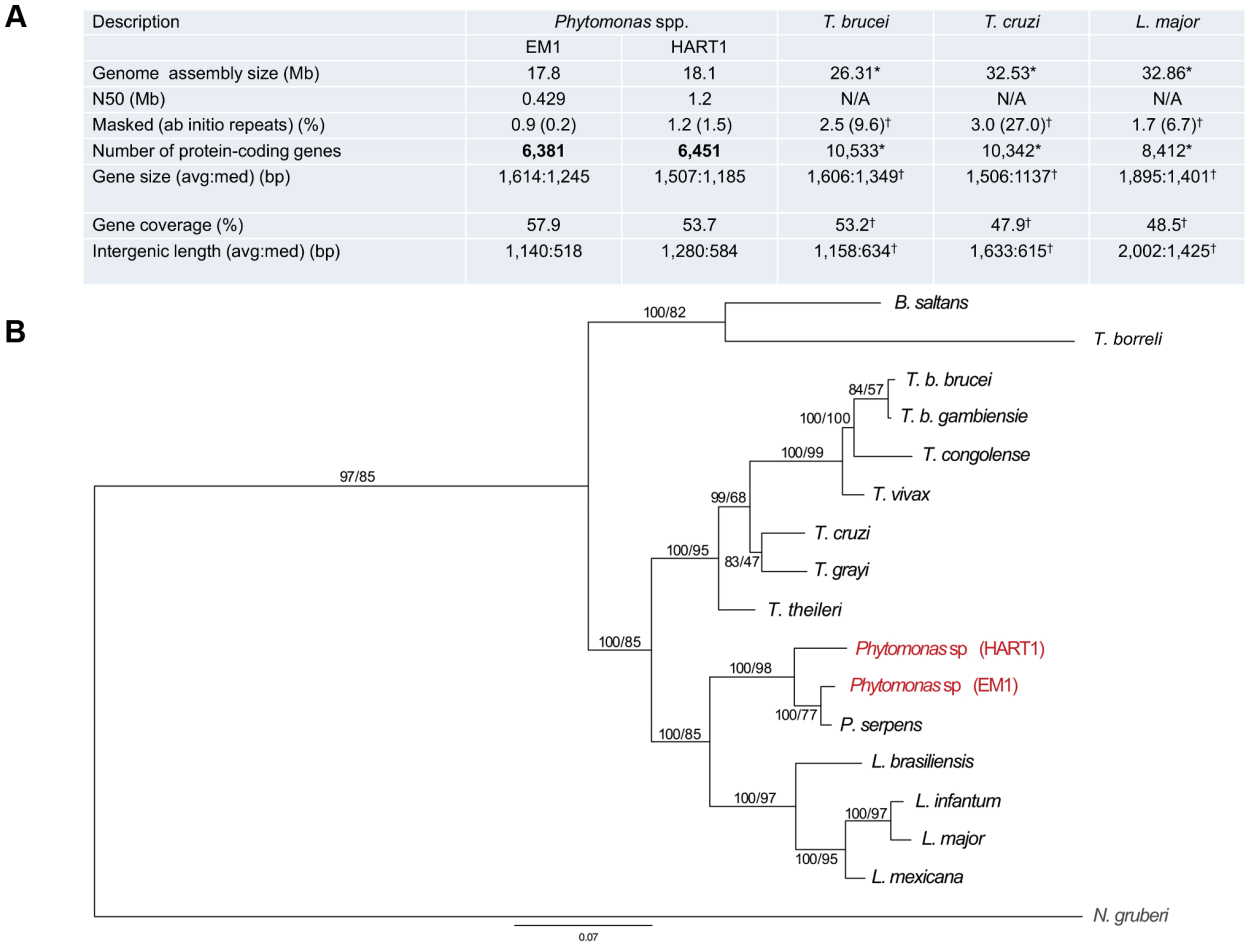
General features of *Phytomonas* EM1 and HART1 isolates. A. Statistics on *Phytomonas* EM1 and HART1 genome annotations. Results of both *Phytomonas* genome annotations, together with statistics on *T. brucei* TRE927 (*T. brucei*), *T. cruzi* CL Brener Esmeraldo-like (*T. cruzi*) and *L. major* Friedlin (*L. major*) genome annotations, either obtained by directly querying the TriTrypDB release 4.2 (*) or by using the same analysis pipeline applied for both *Phytomonas* spp. (†), are summarized; B. Phylogenetic reconstruction of HSP90 evolution in the trypanosomatids. HSP90 sequence data are taken from [127], together with the top BLAST hits retrieved from HART1 and EM1 genome sequence data using HSP90 as a query. Orthology was established by reverse BLAST into the non-redundant database. Trees were constructed by multiple sequence alignment followed by trimming of the N- and C-termini (the EM1 sequence is truncated), and reconstructions by Mr Bayes and PhyML. Statistical support is shown for all nodes as Mr Bayes/PhyML posterior probabilities/bootstraps, respectively. The two *Phytomonas* isolates analysed here are colored in red, and the *N. gruberi* sequence, included as an outgroup, is in gray. Note that branch lengths indicate that EM1 and HART1 are of similar divergence as *T. brucei brucei* versus *T. congolense* or *L. infantum* versus *L. mexicana*. EM1 is more closely related to *P. serpens* than to HART1 based on this dataset.

Recently, the molecular karyotype of several different latex plant symbiont-like (i.e. not associated with apparent pathology in the host) *Phytomonas* isolates were analyzed by pulsed-field gel electrophoresis (PFGE), showing 21 chromosomal bands for EM1 (group D) [37]. Similar analysis performed on phloem-restricted trypanosomatids allowed the identification of 7 chromosomal bands for the Hartrot wilt pathogen isolate (HART1, Group H) [38]. A systematic genome sequencing project of these two *Phytomonas* isolates was initiated, as they represent two distinct phenotypes in terms of impact on the host. Both EM1 and HART1 genomes were assembled using 10× 454-technology and 0.1× Sanger reads, together with deep coverage Illumina sequencing reads for correction of sequencing errors [39] (European Nucleotide Archive accession numbers CAVQ010000001-CAVQ010001400 for EM1 and CAVR010000001-CAVR010002560 for HART1; details in Text S1). Ninety percent of the EM1 genome assembly was placed in 45 scaffolds longer than 100 kb, with one third in the size range of the *Phytomonas* EM1 chromosomes previously observed by PFGE [37]. In the case of EM1, the scaffold N50 (the scaffold size above which 50% of the total length of the sequence assembly can be found) was 429 kb (Figure 1A). Meanwhile, the scaffold N50 for HART1 isolate was 1.2 Mb, with 90% of the genome located in 15 of the scaffolds, again in the size range previously estimated for the HART1 chromosomes [38] (Figure 1A). These assembly statistics indicate that majority coverage of both EM1 and HART1 genomes was achieved. A striking feature of these two plant parasite genomes is their small size (18.1 Mb for HART1; 17.8 Mb for EM1), when compared to that of the human pathogenic trypanosomatids (26.3 Mb for *T. brucei*; 32.5 Mb for *T. cruzi* and 32.9 Mb for *L. major*) [31].

*Phytomonas* EM1 and HART1 are likely fundamentally diploid with some supernumerary chromosomes, with an unknown level of polymorphism between the two haplotypes [37], [38], a feature they have in common with other trypanosomatids. The massively parallel sequencing strategy provided important read depth coverage across both EM1 and HART1 assemblies (Table S1), which was used to establish ploidy for both *Phytomonas* genomes (details in Text S1). Median read depth analysis revealed an even depth across both *Phytomonas* assemblies (Figure S1), pointing towards an underlying euploidy of diploid for both *Phytomonas* isolates. The use of allele frequency for heterozygous single nucleotide polymorphisms (SNPs) across the scaffolds revealed a consistent diploid distribution of frequencies (Figure S2; method as described by [40]). Clusters of duplicated genes were found to be biased towards disomic scaffolds using a Monte Carlo simulation (*p* = 7×10^−4^ hypergeometric distribution). These results were similar to the distribution of multicopy genes observed in *Leishmania* spp. chromosomes [40], suggessting the existence of separate mechanisms for gene duplication and chromosome (scaffold) duplication in *Phytomonas* spp.

Nonetheless, read depth reached values greater than twofold in some cases (scaffolds 24 and 25 in Figure S1 A; scaffolds 13 and 22 in Figure S1 B), probably indicating, as for other parasite genomes, aneusomy of certain chromosomal regions (Figure S3 and S4) [40], [41]. Possible aneusomy was already envisaged for the *Phytomonas* HART1 isolate after study of its molecular karyotype [38]. This increase in read depth is not likely due to the amplification of specific regions of the scaffolds, since read depth was constant along the whole of both the disomic and tetrasomic regions (Figure S5).

Both assemblies were annotated using a combination of evidence (Table S2; for details, see Text S1), with the major features of the genome annotation presented in Figure 1 A. The reference annotation of the *Phytomonas* EM1 and HART1 genomes (European Nucleotide Archive Accession HF955061–HF955198 for EM1 and HF955199–HF955282 for HART1) harbor 6,381 and 6,451 putative protein-coding genes, covering 57.9 and 53.7% of the genome respectively (Figure 1 A). The total number of predicted genes in both *Phytomonas* isolates is lower than in other sequenced trypanosomatids (EuPathDB-TriTryp 4.2: *T. cruzi*, CL Brener Esmeraldo-like 10,342; *T. cruzi* CL Brener Non-Esmeraldo-like 10,834; *T. brucei* TREU927, 10,533; Figure 1 A), but slightly closer to the *Leishmania* spp. (EuPathDB-TriTryp 4.2: *L. braziliensis*, 8,357; *L. infantum*, 8,241; *L. major*, 8,412, Figure 1 A), as expected by the close phylogenetic relationship of *Phytomonas* with *Leishmania* [42]. Such a decrease in predicted gene numbers is the consequence of an almost complete absence of tandemly-linked duplicated genes in both *Phytomonas* genomes as observed when compared to other sequenced trypanosomes [43], [44]. Indeed, the genomes of *T. brucei*, *T.cruzi* and *L. major* contain a high percentage of repetitive genes (Figure 1A; 27% for *T. cruzi*, 9.6% for *T. brucei* and 6.7% for *L. major*), whereas both *Phytomonas* isolates only possess a very low percentage of such genes (Figure 1 A; EM1 and HART1). This is the case for the NADH-dependent fumarate reductase, arranged in several copies in the *T. brucei* (6 copies), *T. cruzi* (7 copies) and *L. major* genomes (4 copies) but only detected as a single-copy gene in both *Phytomonas* isolates (Table S3). The uniform read depth coverage observed all along the *Phytomonas* EM1 and HART1 scaffolds overrules a collapse of multiple tandem repeats into fewer copies during assembly as an explanation for the *Phytomonas* gene copy number observed (Figure S5). A small fraction of EM1 genes were observed in multiple copies on the genome: only 99 clusters of paralogous protein-coding genes (corresponding to 171 genes; for details see Methods) were identified, constituting 2.6% of the *Phytomonas* EM1 putative genes. Typical cases are those of the chaperonin HSP60 (32 copies (on average) in the *T. cruzi* CL Brener genome) and the thioredoxin peroxidase, both identified in three copies in the EM1 assembly. Excluding a multigene family (six genes) with a histone-fold domain, most of the “duplicated” genes were present in only two copies. A similar situation in which the genome was almost exclusively comprised of single-copy genes was observed in HART1, with the exception of a gene family homologous to a major surface metallopeptidase of *Leishmania* promastigotes [45]. The metalloprotease gp63/leishmanolysin (EC 3.4.24.36) was originally described as the most abundant surface protein of *Leishmania* spp, but has been subsequently demonstrated to be pan-eukaryotic. A massive expansion in the gp63 family is evident in HART1 with over 20 members, while EM1 has only two. Both expansions are lineage-specific. GP-63 has been implicated in interactions with both vertebrate and insect hosts of *Leishmania*, and there is preliminary evidence for it playing a role in insect interactions in *P. serpens* and other lower trypanosomatids [46], [47]. In *P. serpens* gp63 is present in many endomembrane compartments; significantly expression levels can be reduced by exposure to fetal calf serum, suggesting an ability to respond to alterations in the environment, and/or potential for degradation of specific proteins or peptides [48].

Unlike the majority of eukaryotes, mRNA transcription in trypanosomatids is polycistronic. These genomes are organized into large polycistronic transcription units (PTUs), with tens –to -hundreds of protein-coding genes arranged head-to-tail on the same DNA strand and apparently transcribed from a single upstream RNA pol II entry site, or promoter [28]–[30], [49]. This unusual gene organization was observed in both *Phytomonas* isolates as well, where genes are organized into 298 (EM1) and 334 (HART1) putative PTUs with an average of 21 (EM1) and 19 (HART1) genes per cistron (Figure S6).

Protein-coding genes in *Phytomonas* appear to lack conventional introns, similar to the structure of genes in other trypanosomatids [1], [50]. Classical cis-splicing introns are documented only in the poly(A) polymerase and an ATP-dependent DEAD/H RNA helicase genes from *T. brucei*, *T. cruzi* [51], and *Leishmania* spp. This striking feature is not conserved in the *Phytomonas* EM1 and HART1 isolates.

Contraction in both plant parasite genomes is also reflected by the short length of the intergenic regions (on average 1,140 bp for EM1; 1,280 for HART1) and a relatively low frequency of repeated sequences (0.9% and 1.2% for EM1 and HART1, respectively) (Figure 1A). No significant difference in overall gene sizes was observed between these isolates (1,614 bp and 1,507 bp on average for EM1 and HART1, respectively). These data suggest that the EM1 and HART1 genomes are compact and might lack many of the expansions of both coding and non-coding sequences that have been described for other trypanosomes [30], [43].

Members of the order Kinetoplastida display an impressive number of structural and biochemical peculiarities. The acquisition of foreign genes through lateral gene transfer is a possible explanation of the trypanosome-specific evolution of novel processes and organization [52]. A systematical search for candidate bacterial horizontal gene transfer (HGT) events (Material and Methods) allowed us to identify 87 HGT candidates in these *Phytomonas* isolates, all shared between the two isolates, with eight of them specific to *Phytomonas* (*i.e.* absent from *Leishmania* and *Trypanosoma*) (Table S4). Several genes of bacterial HGT origin already identified in *Leishmania* were also found in *Phytomonas*, specifically sugar kinases and other genes involved in carbohydrate metabolism, which probably reflects their life cycle in plants and phytophagous insects [52], [53]. All HGT events were common to EM1 and HART1, but a metallocarboxypeptidase of potential bacterial origin was found in only one copy in EM1 and 11 copies in HART1.

In other trypanosomatids, the tRNA genes tend to occur in clusters with a synteny often conserved among different genera (Figure S7; details in Text S1). Most of the tRNA genes predicted for EM1 and HART1 corresponded to those identified previously in *T. brucei*, *L. major* and *T. cruzi* (Table S5). Interestingly, *Phytomonas* isolates possess two tRNAs not found among the animal pathogens, and present in the plant trypanosome branch: they are Asn (ATT)-tRNA (in HART1) and Ser (GGA)-tRNA (in EM1) (Table S5, highlighted in green).

### Kinetoplast DNA genome and transcriptome in *Phytomonas* EM1 and HART1

In all Trypanosomatidae the mitochondrial genome consists of a single network of kinetoplast (k) DNA, one of the most complex organellar genomes known. It is composed of dozens of maxicircles that carry protein-coding and mitoribosomal genes, and thousands of minicircles that encode guide (g) RNAs. The EM1 maxicircle could not be assembled, but a single maxicircle contig of 12,099 bp was recovered for HART1. A homologous 10,478-bp region was sequenced previously for *Phytomonas serpens* [54], and the identity over the matching region of 9,816 bp between the two *Phytomonas* isolates is 76.8%.

Similar to the *P. serpens* maxicircle, the maxicircle of HART1 is characterized by a complete absence of cytochrome *c* oxidase subunits I–III (COI, COII, COIII), and cytochrome b (Cyb) of the bc1 complex. Other maxicircle-encoded genes typical for trypanosomatids, 12S and 9S rRNAs, ND1 to ND5, ND7 to ND9, subunit 6 of ATP synthase (A6), ribosomal protein subunit 12 (RPS12), maxicircle unknown reading frames (MURF) 2 and 5, and unidentified cryptogenes G3 and G4, are present (Figure S8). Since PCR and limited sequencing data indicated that the same deletions are present in EM1 and in three *P. serpens* strains [54], these deletions likely became established at the base of the *Phytomonas* clade.

Some maxicircle-encoded transcripts are known to undergo extensive RNA editing via the insertion and/or deletion of four to hundreds of uridylate residues [55]. Information for the editing process is provided by hundreds of heterogeneous minicircle-encoded gRNAs. The extent of editing is reflected by the sequence identities of individual maxicircle-encoded genes. Although we lack RNA sequence data for HART1, DNA sequence alignments with other kinetoplastids allow determination of the extent of editing for a given gene (Table S6). Genes that are pan-edited in almost all trypanosomatids studied [56] (ND3, ND8, ND9, RPS12, G3, and G4) show no reduction of the edited region in HART1 as compared to *P. serpens* (Figure S8).

When all maxicircle-encoded genes are considered, HART1 and *P. serpens* are more divergent from each other than *L. donovani* is from. *L. tarentolae*, but less so than *T. cruzi* is from *T. brucei*. Furthermore, the HART1 maxicircle genes have slightly lower identity to *L. tarentolae*, *T. brucei* and *T. cruzi* genes, than the genes from these species have among themselves (Table S6). These facts reflect the relatively long branch of the *Phytomonas* clade observed in the SSU rRNA- and glycosomal GAPDH-based phylogenies and deep separation between individual branches of this clade [57], [58].

Recovered full-length kDNA minicircles differ between both *Phytomonas* EM1 and HART1. In HART1 the minicircles range in length from 1,626 to 1,652 bp and contain one conserved region, as does *P. serpens* [59]. The EM1 minicircles are longer (2,791 to 2,819 bp), and carry two conserved sequences opposite each other. These variations are not unprecedented, as the size of minicircles as well as the number of conserved regions are typically uniform within a species, but variable among species [60], [61].

### Transposable elements in the *Phytomonas* EM1 and HART1 genomes

Extensive bioinformatics analyses have been performed for all known transposable elements (TEs) present in the trypanosomatid genomes. While both LTR-retrotransposons (also called retrotransposons) and non-LTR retrotransposons (also called retroposons) were described in the genome of *T. brucei*, *T. congolense*, *T. vivax*, *T. cruzi*, and *Leishmania* spp. (∼3% of nuclear genome), no transposons have been identified to date [31], [32], [62]–[67].

Significantly, there is evidence for involvement of non-autonomous TEs in the regulation of gene expression [65]. *Leishmania* spp. (∼2,000 copies per haploid genome), but not trypanosomes, have domesticated and expanded these small TEs, named SIDER (Short Interspersed DEgenerated Retroposon) and co-opted them as part of the gene expression machinery. All trypanosome species analysed so far contain at least one putative functional TE family of the ingi clade (Tbingi, Tvingi, Tcoingi, L1Tco, L1Tc) that may have the capability to be mobilized, but all members of the ingi clade are degenerate and non-functional in the *Leishmania* species sequenced to date. Two questions were considered important to address in the analysis of TEs in these *Phytomonas* isolates due to their relatively close phylogenetic position to *Leishmania* spp.: when, in the course of trypanosomatid evolution, did domestication and expansion of SIDER occur? and when was the loss of TE functionality from the ingi clade?

**Figure 2:**
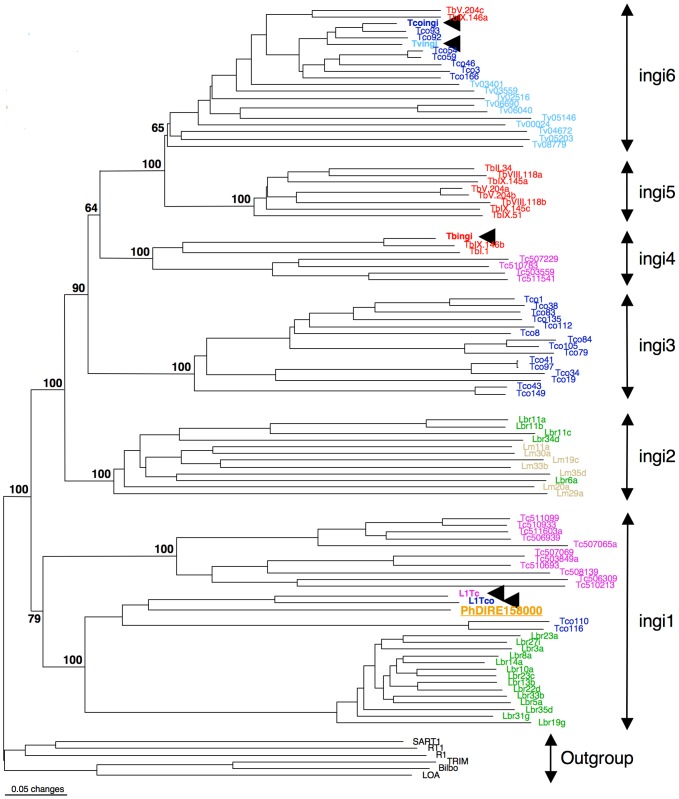
Phylogenetic tree of the reverse transcriptase domain of retroposons belonging to the ingi (from the Kiswahili root adjective meaning ‘many’) clade. The potentially active transposable element (TE) are indicated by an arrowhead. The other elements are DIRE from *T. brucei* (Tb), *T. congolense* (Tco), *T. vivax* (Tv), *T. cruzi* (Tc), *L. major* (Lm), *L. braziliensis* (Lbr) or *Phytomonas* (Ph). This consensus tree was generated with the neighbor-joining method and rooted with the RT domain of retroposons belonging to other clades. All numbers next to each node indicate bootstrap values as percentage out of 100 replicates corresponding to the tree generated with the neighbor-joining method. The ingi subfamilies nomenclature was defined before in [62].

As observed for *Leishmania* spp., both *Phytomonas* genomes are missing potentially active ingi-like TEs, but contain a few non-functional TEs of the retroposon ingi clade. Two types of TEs belonging to the retroposon ingi clade (PhDIRE, for Phytomonas Degenerated Ingi-Related Element, and PhSIDER, Table S7) were identified, with no evidence of functional elements, since all are likely to be inactivated by the accumulation of deletions, point mutations and/or frame shifts. PhDIRE belongs to the ingi1 subclade, considered as an early diverging ingi subfamily also present in *Leishmania* spp., *T. cruzi* and *T. congolense* [66], as shown by phylogenetic reconstruction (Figure 2) and analysis of the conserved motif upstream of the retroposons. PhSIDERs are short elements that were probably derived from PhDIRE by deletion, as previously proposed for other potentially active ingi-like TEs [62], [65], [66], [68] (see Text S1 for details). No sequences related to other trypanosomatid TEs were detected in the *Phytomonas* genomes (details in Text S1).

The EM1 genome was found to contain 41 DIREs, similar to all other trypanosomes and *Leishmania* spp. (*L. major*: 52 and *L. braziliensis*: 65) (Table S7), however the seven SIDER copies was low in comparison to *Leishmania* spp. that carry around 2000 copies. Thus, the enormous expansion and domestication of SIDER in *Leishmania* spp. [65] is not observed in these *Phytomonas* isolates, and exaptation of SIDER was likely a *Leishmania*-specific event in the trypanosomatid lineage.

The HART1 genome is depleted of TEs. Forty-eight retroposons were identified in the EM1 genome, while two PhDIREs were found in the HART1 genome, a 24-fold difference (Table S7). Indeed, both the un-annotated contigs and the non-assembled reads showed very low coverage of PhDIRE/PhSIDER in HART1, confirming the low number of retroposons in this *Phytomonas* isolate.

### High gene content and synteny conservation between EM1 and HART1

**Figure 3:**
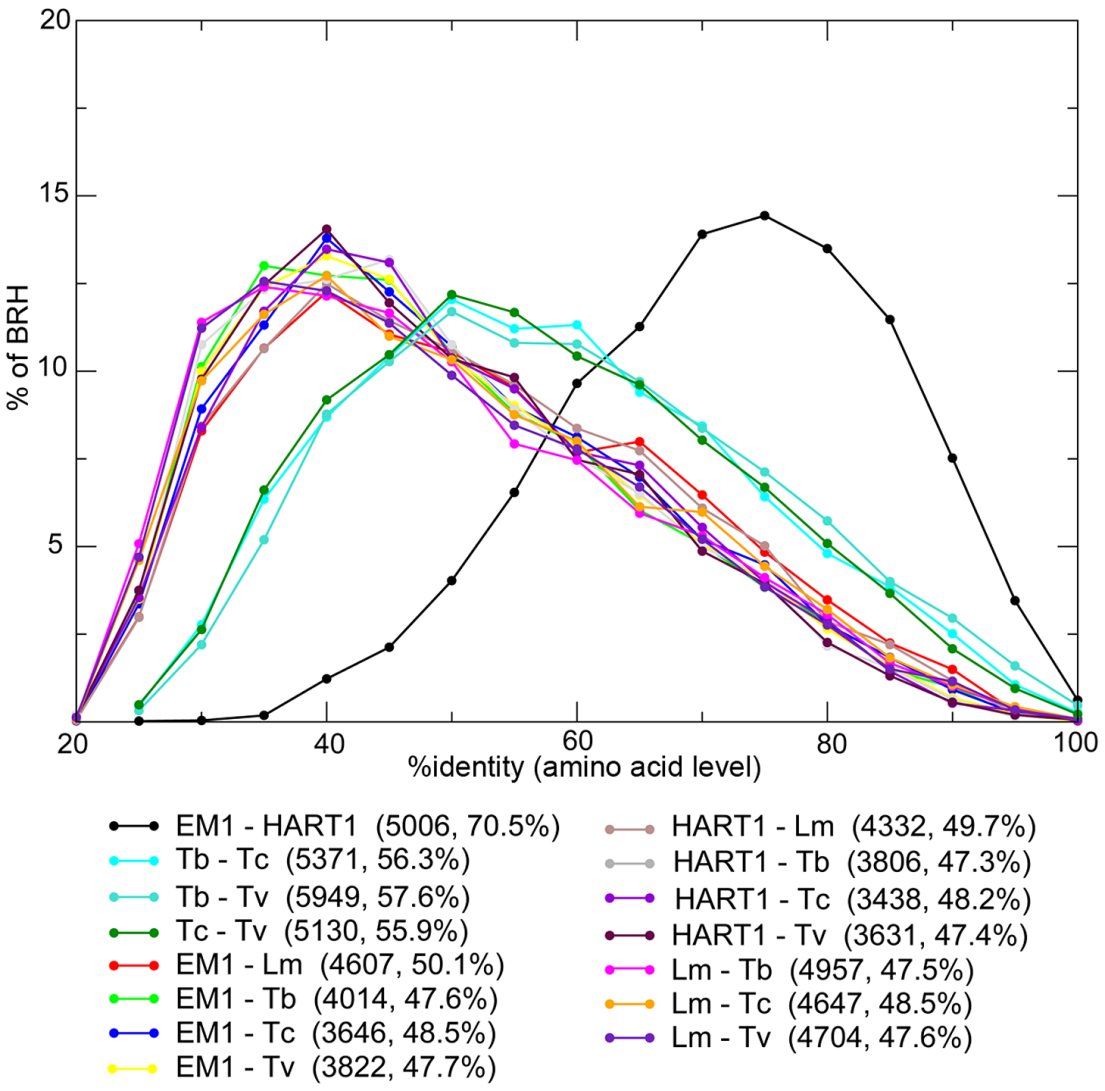
Distribution of the percentage of identity between orthologous proteins for different pairs of organisms. *Phytomonas* EM1 (EM1), *Phytomonas* HART1 (HART1), *L. major* (Lm), *T. brucei* (Tb), *T. cruzi* (Tc) and *T. vivax* (Tv). For each pair of species, the number of Best Reciprocal Hits (BRH) and their average % identity (comparison done at amino acid level) are displayed between parentheses.

The majority of *Phytomonas* genes are shared between both isolates, as shown by independent approaches used for ortholog detection (see Materials and Methods). The combination of both Best Reciprocal Hits (BRH) and orthoMCL strategies identified 5,210 (82%) genes from EM1 with orthologs in HART1, and 5,108 (79%) genes from HART1 with counterparts in EM1, similar in gene size (Figure S9 A). The *Phytomonas* EM1 and HART1 orthologs were more closely related to each other than to their trypanosome orthologs with an average percentage of identity of 70.5% (Figure 3). The small nucleolar RNA (snoRNA) repertoires of HART1 and EM1 also showed higher similarity to each other than to *T. brucei* or *L. major* (Table S8).

The genes for which no orthologs could be detected by this preliminary approach are excellent candidates for understanding *Phytomonas* spp. behaviors. After eliminating genes for which orthologs were not detected because of annotation or assembly issues, as well as suspected annotation artifacts, 13 genes remained in EM1 and 4 in HART1 that could be confidently considered as lacking an ortholog in the other isolate (Figure S10 and Table S9, see Materials and Methods for details). The vast majority of *Phytomonas* genes are shared between both isolates, highlighting the high level of conservation of the gene repertoire between these two trypanosomatids.

**Figure 4:**
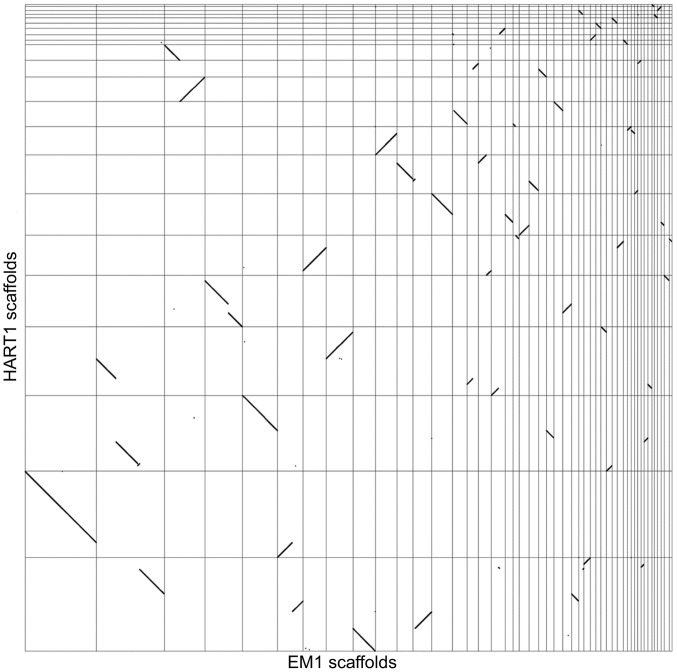
Synteny between *Phytomonas* EM1 and HART1 genomes. Dot plot representation of the 5,006 BRH between EM1 and HART1. Each dot represents a pair of genes (BRH), with on the *x* axis the position of the EM1 gene on the EM1 assembly (from left to right: scaffold 1–36, 38, 39, 42–46, 49, 52, 54, 55 and 57), and on the *y* axis the position of the HART1 gene on the HART1 assembly (from bottom to top: scaffold 1–13,15–19, 22–24 and 26).

We analyzed synteny between EM1 and HART1 using dot-plots (Figure 4). Synteny was conserved between EM1 and HART1, with most of the synteny breaks corresponding to scaffold boundaries in one of the two isolates. Only five *bona fide* synteny breaks with HART1 were found in the EM1 assembly, and 10 in the HART1 assembly. The syntenic blocks are large (average of 60 genes, median of 35 genes) and usually include several hypothetical PTUs (average 20 ORFs, median of 10) (Figure S11). There is good conservation between PTUs in EM1 and HART1, with at least one boundary in common between EM1 and HART1 for all PTUs (Figure S12A and Figure S13). Significantly, synteny breaks tend to correspond to the boundaries between putative PTUs (Figure S12B), and intergenic distances are well conserved (Figure S9B). To identify putative insertions in one isolate compared to the other, we searched for gene number differences between successive pairs of BRH in syntenic PTUs (Materials and Methods). After filtering possible annotation artifacts (genes missed, splits/fusions, etc) and genes with strong sequence similarity elsewhere in the genome (Table S10), we retained ten genes in EM1 absent at the syntenic position in HART1, including three already identified as lacking a HART1 ortholog. Furthermore, three genes in HART1 lack a syntenic equivalent in EM1, with two already identified as having no ortholog in EM1 (Table S9). The two strategies did not identify the same sets of genes because of slight differences in the very conservative quality controls applied (see Material and Methods). Significantly, ten and three genes in EM1 and HART1 respectively, displayed weak hits in the syntenic region, suggesting that they have diverged in the other isolate; ten and two genes had no evidence for sequence homology, and could thus correspond to insertions or complete deletions. Combining the two approaches, 20 genes from EM1 were confidently determined to be absent from HART1 and 5 genes from HART1 were found to be absent from EM1 (Table S9). Since we could only compare assembled and annotated genes with confidence, these numbers may be underestimates of the true number of non-conserved genes between both isolates, but they are representative of the overall level of synteny and gene repertoire conservation between these two phylogenetically remotely related *Phytomonas* isolates (Table S9).

### Comparison of *Phytomonas* with other trypanosomatids

OrthoMCL comparisons [69] were performed between *Phytomonas* EM1 and HART1, and four other trypanosomatids: *L. major* [29], *T. brucei* [28], *T. cruzi* [30] and *Trypanosoma vivax* [70] (Materials and Methods). This predicted 22,706 clusters of orthologous genes. Their conservation profiles (i.e. the list of species in which they are found) are shown in Table 1. A core of 2,869 genes was conserved between all six species (Table 1). Indeed, expert examination of this group of genes showed that 80.6% of the identified protein kinases shared by both *Phytomonas* isolates are also present in *T. brucei* and *L. major*. This subgroup contained major regulators, including up to 11 cdc2-related kinases (CRKs), WEE1, aurora kinase AUK1, glycogen synthase kinase 3 (GSK3) and casein kinases CK1 and CK2, expected to be present in all eukaryotes (Table S3C). Putative amino acid transporters conserved in all four mammalian parasites were also identified in these *Phytomonas* isolates. Interestingly, both isolates contained the same repertoire of amino acid transporters (AAPs), but with differing copy numbers (Table S3E; details in Text S1). Several genes with similarity to calmodulin and genes annotated as calmodulin-like in *T. cruzi* [71] were also present in both *Phytomonas* genomes.

**Table 1:** Gene conservation among Kinetoplastidae.

Conservation profile	Number of genes	Conservation profile	Number of genes	Conservation profile	Number of genes
Tv	4039 (4042*)	Lm,Tb,Tc	57	EM1,HART1,Tb,Tc	7
Tb	3610 (3612*)	Tc,Tv	48	HART1,Lm,Tc	6
Tc	3084 (3093*)	EM1,HART1,Tb,Tc,Tv	47	EM1,Tc	5
EM1,HART1,Lm,Tb,Tc,Tv	**2869**	EM1,Lm	46	HART1,Tc	4
Lm	2628 (2705*)	EM1,Lm,Tb,Tv	41	EM1,Lm,Tb	4
HART1	1459 (715*)	HART1,Lm,Tb,Tv	35	HART1,Tb,Tv	3
EM1	794 (226*)	EM1,HART1,Lm,Tc,Tv	31	HART1,Tb,Tc,Tv	3
Tb,Tc,Tv	738	HART1,Lm	25	HART1,Tb	3
EM1,HART1	671	EM1,HART1,Lm,Tb	24	HART1,Lm,Tv	3
Lm,Tb,Tc,Tv	479	EM1,Lm,Tb,Tc	23	EM1,Tb,Tv	3
EM1,HART1,Lm,Tb,Tv	437	EM1,Lm,Tc	18	HART1,Tv	2
EM1,HART1,Lm	246	Lm,Tc,Tv	16	HART1,Lm,Tb	2
EM1,Lm,Tb,Tc,Tv	235	Lm,Tv	13	EM1,Lm,Tc,Tv	2
HART1,Lm,Tb,Tc,Tv	205	EM1,HART1,Tc	12	EM1,HART1,Tb	2
Tb,Tv	178	EM1,HART1,Lm,Tv	12	EM1,Tv	1
Lm,Tc	122	Lm,Tb	11	EM1,Tc,Tv	1
Tb,Tc	117	HART1,Lm,Tc,Tv	10	EM1,Tb,Tc	1
EM1,HART1,Lm,Tb,Tc	101	EM1,HART1,Tb,Tv	9	EM1,Lm,Tv	1
Lm,Tb,Tv	77	HART1,Lm,Tb,Tc	7	EM1,HART1,Tv	1
EM1,HART1,Lm,Tc	71	EM1,Tb,Tc,Tv	7		

Orthologs genes were identified between *Phytomonas* EM1 and HART1 and 4 other trypanosomes: *T. brucei* (Tb), *T. vivax* (Tv), *T. cruzi* (Tc) and *L. major* (Lm). The results of the pairwise alignments between all protein sequences of the 6 genomes were analysed using orthoMCL, as described in Materials and Methods. The same analysis was performed keeping only EM1 and HART1 genes with strong support (*).

Manual inspection of *Phytomonas* gene families highlighted many examples of gene conservation within these plant parasites. Four conserved *Phytomonas* EM1 and HART1 kinases were absent in both *T. brucei* and *L. major*: These *Phytomonas*-specific kinases were one calcium/calmodulin regulated kinase-like, one UNC-51-like kinase, and two unique kinases that do not fall into any defined kinase group (Table S3C; details in Text S1), suggesting that these enzymes could be important for infection of, or survival in, plants. Conservation of the phosphatase complements was also observed in these two isolates; only slight differences were detected between both tyrosine and serine/threonine-specific complements (Table S11; details in Text S1).

The *Phytomonas* isolates have more genes in common with *Leishmania* than with the three *Trypanosoma* spp.: 317 orthoMCL clusters are shared between at least one *Phytomonas* isolate and *L. major* but none of the *Trypanosoma* spp., and only 111 clusters are common to at least one *Phytomonas* isolate and one *Trypanosoma* spp. but not *L. major*. The number of BRH, as well as their percentage of identity, was also significantly higher between *Phytomonas* and *Leishmania* than between *Phytomonas* and trypanosomes (Figure 3). However, the presence of two types of clusters conserved only in *Trypanosoma* or *Leishmania* suggests independent secondary losses from an ancestral organism with a substantially larger gene complement.

Significant synteny was observed between *Phytomonas* and *Leishmania* (Figure S14), as well as between *Phytomonas* and trypanosomes (Figure S15, Figure S16 and Figure S17). As expected from the closer phylogenetic relationship of *Phytomonas* with *Leishmania* (Figure 1B) [37], [38], [42], more syntenic breaks were observed between the *Phytomonas* isolates and trypanosomes than *Leishmania* (Figure S12B). Syntenic blocks usually include several PTUs (Figure S13). We compared the number of synteny breaks that occur at PTU boundaries with what would be expected by chance (Materials and Methods): for all pairs of species, the synteny breaks tended to coincide with PTU boundaries (Figure S12B). The high synteny conservation between trypanosomatids might thus be the result of a selective pressure against intra-PTU rearrangements.

### Proteins involved in kDNA replication, kRNA editing, modification and translation

The topological complexity of the kDNA network has fascinated replication specialists for decades. The process is not fully understood, but many of the players have been identified. In the model flagellate *T. brucei*, the machinery is extremely complex, requiring the combined activity of several mitochondrial DNA polymerases, ligases, endonucleases, helicases and topoisomerases [72]. Using a database of 26 genes encoding the kDNA replication machinery of *T. brucei*, all orthologs have been identified in the *Phytomonas* EM1 and HART1 isolates.

The transcripts of many maxicircle genes undergo RNA editing in order to be translatable on mitochondrial ribosomes. Editing and processing of these mRNAs require the participation of several dozen proteins. A list of 28 *T. brucei* orthologs that are confirmed components of the RNA editing core complex or predicted to interact transiently with the complex [73] revealed that both EM1 and HART1 have the same composition, with substantial similarity to *T. brucei*. With the exception of KREP4, KREP5 and the oligoU-binding protein that have likely been lost or divergent as in *L. major*, all of the remaining orthologs are present. In both *Phytomonas* isolates, KREPB7 is duplicated. The available data is compatible with the existence of another complex involved in RNA editing, mitochondrial RNA binding complex 1 (MRB1) being composed of transiently interacting sub-complexes, with up to 32 components [74]. While only recently identified, MRB1 and associated proteins are conserved, as EM1 and HART1 contain all of its known orthologs.

Trypanosomatid flagellates are well known for their uniquely complex kDNA and kRNA. All in all, the gene order, editing patterns, as well as proteins that participate in the metabolism of these organellar nucleic acids, mostly identified in model species *T. brucei*, *L. tarentolae* and/or *C. fasciculata*, are conserved in these *Phytomonas* isolates.

### Phosphorylation, calcium uptake and transporters in *Phytomonas* spp.: Examples of genome contraction in both EM1 and HART1 isolates

Analysis of the *Phytomonas* genome sequences provided a global view of the metabolic potential of plant trypanosomatids. Comparison of the gene repertoires from both isolates to other sequenced trypanosomatids revealed a simplified genome, coding for a minimal system with a clear lack of complexity for each isolate. Indeed, both EM1 and HART1 genomes presented diminutive gene sets when compared to *T. cruzi*, *T. brucei* and *L. major* (Table 2, for more details see Table S3), retaining only the most essential functions for the parasite, and often including a considerable fraction of genes that could serve the hosts. Furthermore, both gene repertoires are reduced as a result of both the loss of entire gene families and the reduction of the numbers of paralogs within gene families.

**Table 2:** Gene repertoires in *Phytomonas* EM1and HART1 isolates.

				*Phytomonas*	
Expert annotation	Tb	Tc	Lm	EM1	HART1
**Calcium transporters**					
Calcium Pumps and Channels	10	19	8	10	10
Calcium Binding Proteins	16	24	12	8	8
V-ATPase subunits	15	27	17	14	14
Ca signaling	13	27	14	14	14
Phosphate	6	8	4	3	3
**Metabolism**					
amino acid metabolism	50	120	72	39	36
carbohydrate metabolism	54	84	58	44	53
glycolysis	18	12	11	7	7
glycosilation	10	26	14	10	10
phospholipids metabolism	18	26	20	14	14
lipid metabolism	33	62	44	36	28
ascorbate biosynthesis	11	21	15	10	10
folate metabolism	2	8	6	4	4
isoprenoid metabolisme	10	19	9	12	11
oxidant stress	19	25	23	14	13
PEX	11	16	11	10	10
Polyamine	3	6	4	3	3
PPP	8	16	8	7	7
purine and pyrimidine metabolisme	21	44	22	20	20
energetic metabolisme	114	127	100	57	56
RNAi	3	0	1	0	0
fatty acid metabolism	38	63	50	27	21
**Phosphatome**					
PTP family	24*	30*	30*	22	23
STP family	54*	56*	58*	45	45
**Trafficking proteins**					
epsin-like and dynamins	5*	4*	2*	2	2
Clathrins	2*	5*	2*	2*	2
Adaptins	12*	29*	13	12	12
COPs	17*	28*	17*	17	16
Retromers	5*	8	5*	5	5
Tethers	30*	55*	29*	30	29
ESCRTs	16*	29*	16*	16	16
SNAREs	26*	48*	26*	25	23
LPG+GPI biosynthesis	13*	96*	29*	15	17
**Kinases**					
AGC	10	11	15	11	11
CAMK	15*	23	15*	13	13
CK1	8	11	7	7	6
CMGC	40	76	44	37	36
Other/AUR	3	3	3	3	3
Other/CAMKK	4*	8	4*	3	3
Other/CK2	2	3	2	2	2
Other (NEK)	20*	27	23*	17	17
Other (PEK)	2	5	3	3	3
Other (PLK)	2	5	2	3	3
Other (TLK)	2	2	1	1	1
Other/ULK	2	3	2	2	2
Other (VPS15)	1	1	1	1	1
Other (WEE)	1	2	2	1	1
Other/kinase accessory proteins	6	2	3	3	3
STE	25	41	33	24	25
Cyclins	10	13	11	7	9
Unique	24*	39	42*	22	22
**Transporters**					
amino acid transportes	78	26	24	15	16
sugar transporters	22	4	4	1	1
ABC transporter families	22*	28*	ND (42*)	24	23

The members of selected *Phytomonas* EM1 and HART1 gene families were identified using specific gene sequence as probes, as described in Materials and Methods. *T. brucei* (Tb), *T. cruzi* (Tc) and/or *L. major* (Lm) gene copy number was obtained, when possible, from literature and/or human curation (*). Otherwise, gene copy number was computed based on OrthoMCL v5 (details in Materials and Methods). ND, not determined.

The protein kinase contents of the *Phytomonas* isolates provide a good example of genome contraction in these plant parasites: eukaryotic protein kinase (ePKs) genes were identified in both isolates (160 and 161 in EM1 and HART1, respectively), but in smaller numbers than in the TriTryp kinomes (Table 2) [31], [75]. Twenty four protein kinases, conserved in *T. brucei* and *L. major*, were not present in either of the *Phytomonas* draft kinomes. (Table S3C). Furthermore, nine *T. brucei*-only kinases and 24 *L. major*-only kinases were also absent from both *Phytomonas* draft kinomes. Even though it is possible that fewer ePKs are required for infection of plants compared to mammals, the similar number of ePKs in the pathogenic isolate HART1 was somewhat unexpected, as it could be considered that additional protein kinases might be required to coordinate virulence factor expression.

The less investigated partners of the phosphorylation-dephosphorylation regulatory cascades are the protein phosphatases, organized into four major groups, depending on substrate preferences and catalytic signature motifs. Three of these groups corresponds to Ser/Thr specific phosphatases (STP): metallo-dependent protein phosphatases (PPM), phosphoprotein phosphatases (PPP) and aspartate based phosphatases with a DxDxT/V motif. The fourth group corresponds to the protein tyrosine phosphatases (PTP) [76]. The completion of the genome sequences of *L. major*, *T. brucei* and *T. cruzi* [31] has permitted a deeper analysis of the protein phosphatases, showing that the main protein phosphatase groups (Tyr, Ser/Thr and dual specific protein phosphatases) are present in these parasite genomes, as in higher eukaryotes [77].

The *Phytomonas* phosphatome provides another illustration of the genome reduction observed in these parasites. Comparing the two plant trypanosomes' phosphatomes to the TriTryp phosphatome [78], the main differences were found in the PTP complements: the eukaryotic-like PTPs were absent from both EM1 and HART1 phosphatomes, and no orthologs of PTENs and CDC14s [76] have been identified (Table S11A). PTENs and CDC14s (dual specific phosphatase group) are present in the phosphatomes of all three other kinetoplastids, where they can be grouped into two distinct families, the eukaryotic-like and kinetoplastid-like PTENs, depending on their sequence homology to other eukaryotic PTENs. One kinetoplastid-like PTEN enzyme has been found in the three kinetoplastids *T. cruzi*, *T. brucei* and *Leishmania* [79]. While four eukaryotic-like PTENs have been identified in *T. cruzi*, only one enzyme was found in *L. major*. Interestingly, no *T. brucei* ortholog was identified, thus suggesting a possible role of these enzymes in intracellular parasitism.

**Figure 5:**
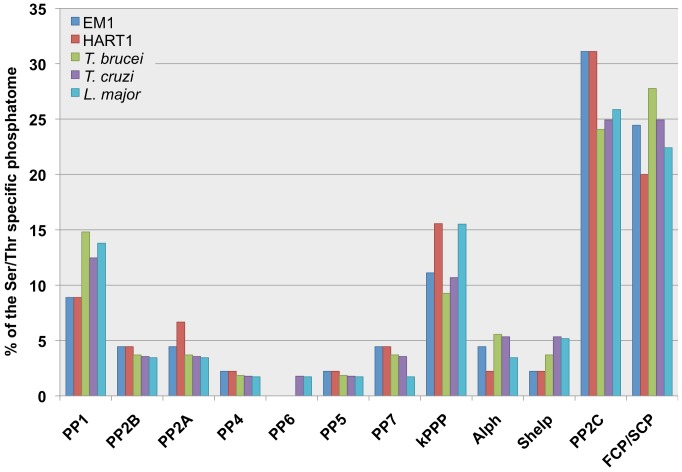
Comparison of the plant trypanosomes' and the TriTryp Serine/Threonine protein phosphatase (STP) complements. The bar graphs show the different STP genes distribution (%) in the Serine/Threonine protein phosphatomes of *Phytomonas* EM1 (EM1, 45 genes); *Phytomonas* HART1 (HART1, 45 genes); *Trypanosoma brucei* (*T. brucei*, 54 genes), *Trypanosoma cruzi* (*T. cruzi*, 56 genes); *Leishmania major* (*L. major*, 58 genes). The abbreviations for the STP families: Protein Phosphatase type 1 (PP1), Protein Phosphatase type 2B/calcineurin (PP2B), members of Protein Phosphatase type 2 group (PP2A, PP4, PP6), Protein Phosphatase type 5 (PP5), Protein phosphatase type 7/PPEF (protein phosphatases with EF-hand/PP7), kinetoplastid specific STPs (kSTPs), ApaH-like phosphatases (Alphs), Shewanella-like phosphatases (Shelps), Protein Phosphatase Mg^2+^- or Mn^2+^-dependent family members (PP2C) and TFIIF (transcription initiation factor IIF)-associating component of CTD phosphatase/small CTD phosphatase (FCP/SCP). The numbers of STPs are shown in Table S13B. The data of TriTryp phosphatomes was used from [78] to construct the bar graphs.

When we compared the STP complements of the *Phytomonas* isolates, we detected a 20% decrease in the total number of phosphatases as compared to the TriTryps, mainly due to the reduced number of type 1 protein phosphatases. The number of PP1s has been augmented in the genomes of *T. brucei*, *T. cruzi* and *L. major* by a gene duplication process (8/7/8) [78]. Still, the functions associated to these apparently higher number of resembling genes have not been characterized. Both in EM1 and HART1, four genes encoding PP1 catalytic subunits were identified, a similar number to those described in other eukaryote PP1 complements. We have also found a two-fold reduction in the number of the bacterial-like phosphatases, Alphs and Shelps [80] in the plant trypanosomatids compared to the TriTryp phosphatomes (Figure 5, Table S11B).

The reduction in the number of members of ABC transporters (Table S12) and amino acid transporter families in these *Phytomonas* isolates represents another relevant example of genome retrenchment. A unique family of amino acid transporter (AAP) genes from members of the trypanosomatid family (25 in *Leishmania*, 17 in *T. brucei* and 19 in *T. cruzi*) has been identified, based on the existence of amino acid permease pfam domains [81], [82]. This trypanosomatid-specific group of amino acid transporters corresponds to a distinct clade within the amino acid/auxin permease (AAAP) super family [83], [84]. The analysis of these gene families revealed 15 and 16 AAP genes in EM1 and HART1 respectively, fewer than in the mammalian trypanosomatid genomes (Table 2 and Figure S18, details in Table S3E and Text S1).

Eukaryotic cells regulate their cytosolic calcium concentration using numerous channels and transporters located in the mitochondria, the plasma membrane and the endoplasmic reticulum. Additionally, calcium binds to an extensive collection of signaling and regulatory proteins in these eukaryotic cells. In trypanosomatids, acidic organelles known as acidocalcisomes, which have been identified in *Phytomonas françai* [85], act as the major stock of the intracellular calcium, and are implicated in processes such as calcium homeostasis, osmoregulation and polyphosphate metabolism [71]. Hence, both *Phytomonas* EM1/HART1 genomes were investigated for the presence of orthologs to trypanosomatid genes known to be involved in calcium and polyphosphate metabolism.

The trypanosomatid genome projects revealed a vast diversity of Ca^2+^-binding proteins (as an example for *T. cruzi* see Table S3A), many of which are not characterized and have little or no homology with non-kinetoplastid proteins. Regulation of cytosolic Ca^2+^ concentration in *Phytomonas* isolates EM1 and HART1 appears similar to that of other trypanosomatids. Yet, several differences allow to clearly distinguish these organisms (Table S3A). Though the inositol phosphate/diacylglycerol pathway is present in pathogenic trypanosomatids, no evidence of either a phospholipase C, or a protein kinase C was found in these *Phytomonas* isolates. However, there are orthologs to the putative InsP_3_ receptor in both *Phytomonas* EM1 and HART1 isolates. Another interesting difference is the lack of *Phytomonas* counterparts to calreticulin, a Ca^2+^ storage protein located in the endoplasmic reticulum of *T. cruzi* [86], and the recently characterized polyphosphate kinase (vacuolar transporter chaperone 4) of yeast, pathogenic trypanosomatids, and Apicomplexan.

### The membrane trafficking system and the predicted cell surface proteome

**Figure 6:**
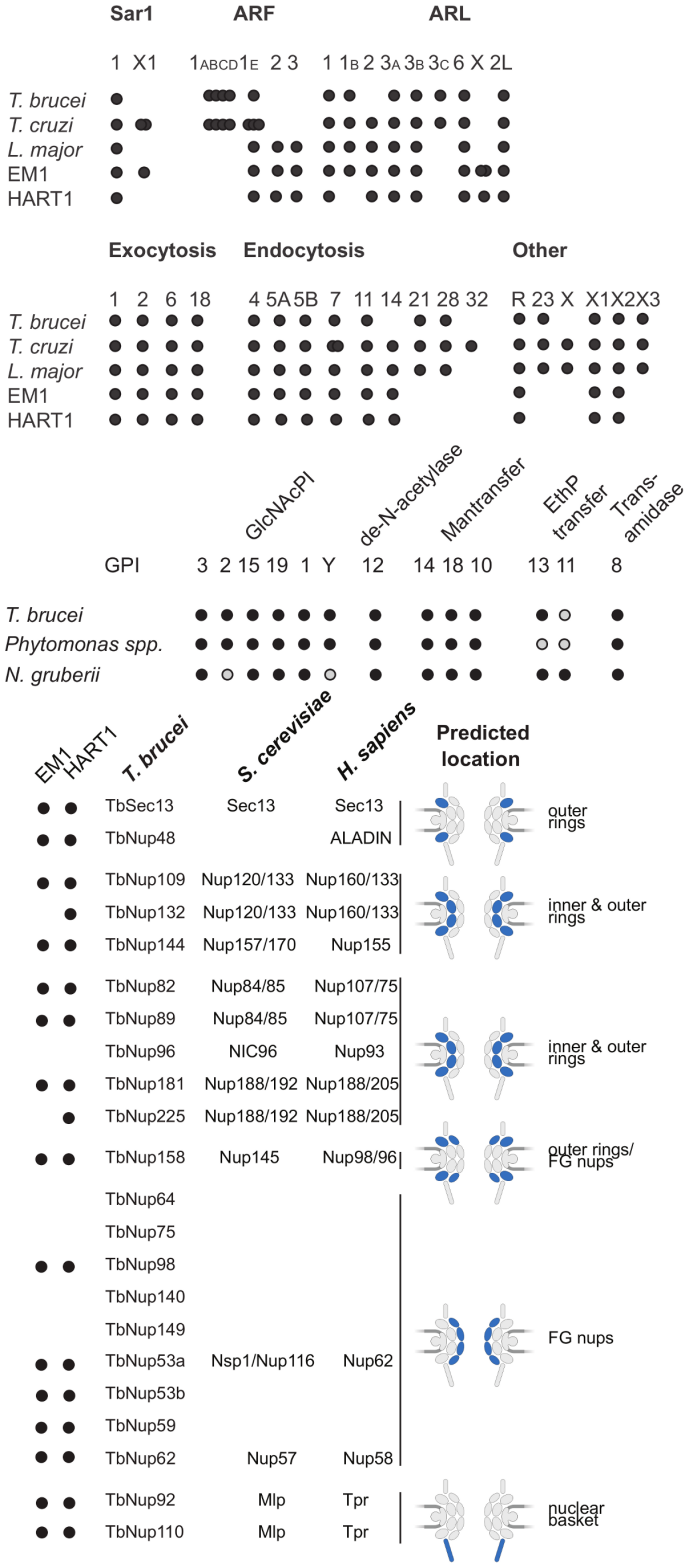
ARF, Rab NUP and GPI pathways in *Phytomonas* EM1 and HART1. Schematic summaries indicating the presence or absence of components of the ARF, Rab NUP and GPI Pathways in *Phytomonas*, the TriTryps and selected comparitor taxa. The overlapping dots correspond to paralogs: *T. cruzi* ARF 1ABCD, 4; *T. brucei* ARF 1ABCD, 4; *T. cruzi* ARF 1E, 3; *T. cruzi* SAR X1, 2; *Phytomonas* EM1 ARL X, 2.

To predict both the level of intracellular organellar complexity and the surface composition of *Phytomonas*, the open reading frame complement of HART1 and EM1 were scanned for around 300 genes involved in membrane trafficking. Both isolates of *Phytomonas* share essentially identical membrane transport systems, with only one clear example of specialization (Table S3D and Figure S19). Overall, the endomembrane systems are the simplest yet described amongst trypanosomatids; for example the Rab GTPase repertoire, a primary determinant of specificity and organelle identity [87], retains the basic core exocytic and endocytic functions and the trypanosome-specific Rab-like X1 and X2 [88] (Figure 6). However, the system is substantially simpler, with only 12 Rab/Rab-like proteins compared to 16 in *T. brucei* or 17 in *L. major* [28], [89]. Given that the losses here are Rab21, 28 and 32, this reduction represents sculpting of the system by secondary loss from the common ancestor and hence is an adaptive streamlining [90]. This simplification is also seen in the secondary loss of the AP4 adaptin sorting complex from both *Phytomonas* genomes (Table S3D), and in a rather simpler ARF GTPase family compared with other trypanosomatids. Further, these data likely suggest a simplified late endocytic system, to which Rab21, Rab28 and AP4 are all assigned. Overall the view is of a minimal endomembrane system, which conserves the major complexes and pathways, indicating retention of all major organelles, but with an apparent lack of complexity or innovation; adaptation has been via minimization rather than invention.

As befits the position of *Phytomonas* as basal within the trypanosomatid lineage, the surface appears to be rather similar to *Leishmania* spp., and there is no evidence for mucin-like or variant surface glycoprotein-related protein coding genes, or a dominant, highly expressed, surface antigen as no predicted GPI-anchored protein was encoded by transcripts in the most abundant RNAseq percentiles. The surface system includes full glycosylphosphatidylinositol (GPI) anchor and glycolipid biosynthetic pathways, the enzymatic apparatus for synthesis of a lipophosphoglycan (LPG)-like molecule and evidence for the GPI-anchored gp63 protein (Table S3D, Figure S19).

### Metabolism in *Phytomonas* EM1 and HART1

**Figure 7:**
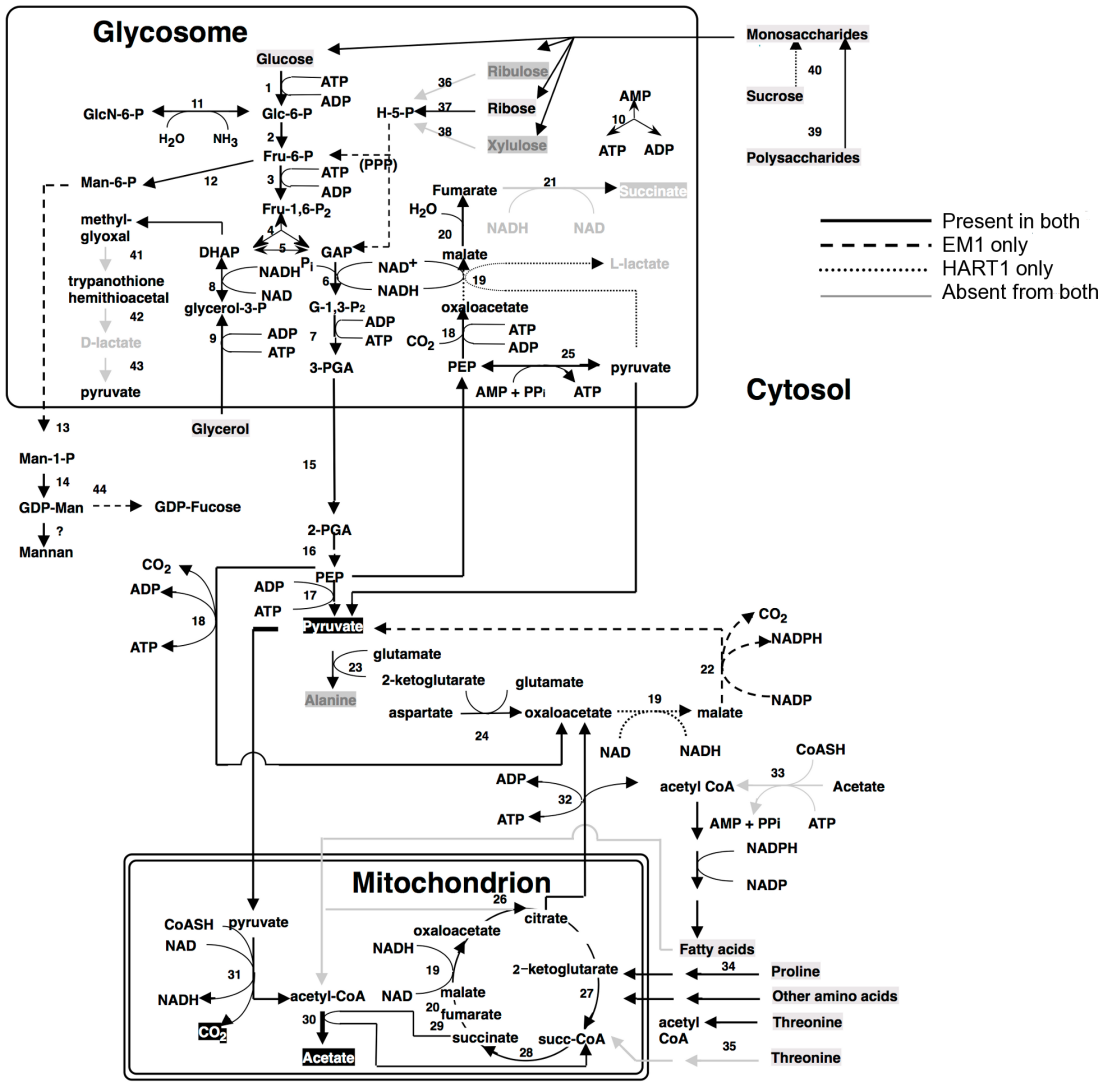
Core metabolism pathways in *Phytomonas* EM1 and HART1, as compared to that of *Leishmania major*. Boxed metabolites are nutrients (in gray) or end-products (in black). PPP, pentose-phosphate pathway. Enzymes: 1, hexokinase; 2, phosphoglucose isomerase; 3, phosphofructokinase; 4, fructosebisphosphate aldolase; 5, triosephosphate isomerase; 6, glyceraldehyde-3-phosphate dehydrogenase; 7, glycosomal phosphoglycerate kinase; 8, glycerol-3-phosphate dehydrogenase; 9 glycerol kinase; 10, glycosomal adenylate kinase; 11, glucosamine-6-phosphate deaminase; 12, mannose-6-phosphate isomerase; 13, phosphomannomutase; 14, GDP-mannose pyrophosphorylase; 15, phosphoglycerate mutase; 16, enolase; 17, pyruvate kinase; 18, phosphoenolpyruvate carboxykinase; 19, malate dehydrogenase; 20, fumarate hydratase; 21, NADH-dependent fumarate reductase; 22, malic enzyme; 23, alanine aminotransferase; 24, aspartate aminotransferase; 25, pyruvate phosphate dikinase; 26, citrate synthase; 27, 2-ketoglutarate dehydrogenase; 28, succinyl-CoA ligase; 29, succinate dehydrogenase; 30, acetate: succinate CoA transferase; 31, pyruvate dehydrogenase; 32, citrate lyase; 33, acetyl-CoA synthetase; 34, proline oxidation pathway; 35, threonine oxidation pathway; 36, ribulokinase; 37, ribokinase;, 38, xylulokinase; 39, glucoamylase; 40, invertase; 41, glyoxalase I; 42, glyoxalase II; 43, D-lactate dehydrogenase.

**Figure 8:**
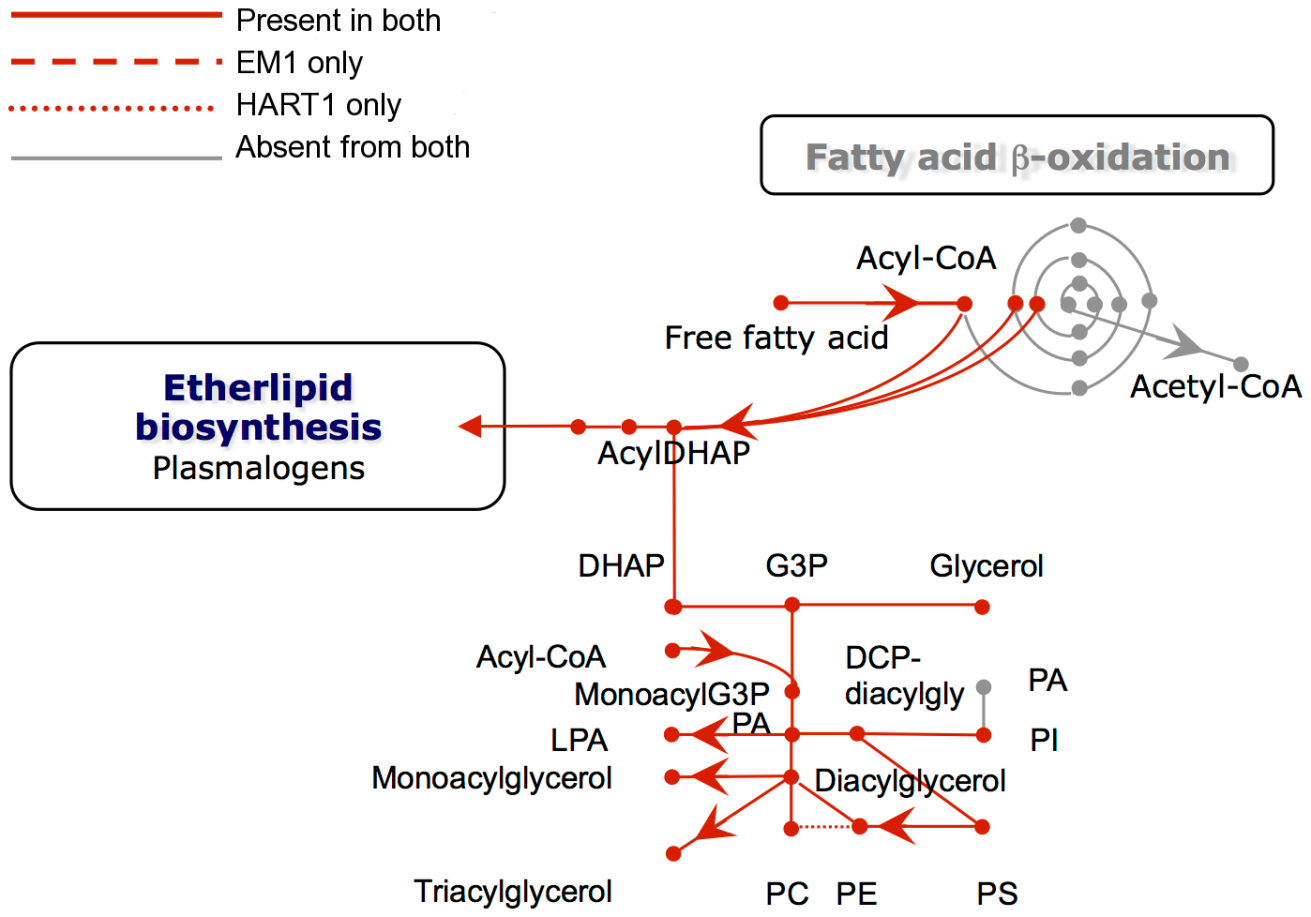
Phospholipid metabolism in *Phytomonas* EM1 and HART1. Reactions involved in the interconversion of fatty acids, ether lipids and phospholipids are shown. CoA, Coenzyme A; DHAP, dihydroxyacetone phosphate; G3P, glycerol 3-phosphate; PA, phosphatidic acid; LPA, lysophosphatidic acid; PC, phosphatidyl choline; PE, phosphatidyl ethanolamine; PS, phosphatidyl serine; Pi inorganic phosphate.

Analysis of the genomes of these two plant trypanosomes provided a global view of the metabolic capacity of *Phytomonas*. As a consequence of an almost complete absence of tandemly-linked duplicated genes, most of the metabolic genes in *Phytomonas* were identified as one haploid copy (Figure 7, Figure 8, Figure S20 and Figure S21; for details see Table S3B).

As part of its carbohydrate metabolism (Figure 7; details in Table S3B), *Phytomonas* not only utilize the plant's sucrose but also its polysaccharide stores as major energy substrates, as confirmed by the identification of genes coding for glucoamylase, alpha-glucosidase and, only in the HART1 isolate, many copies of invertase (beta-fructofuranosidase) homologs (Table S3B).

The presence of an alpha, alpha-trehalose phosphorylase in both isolates suggested that *Phytomonas* is also capable of using the abundant plant disaccharide trehalose for its carbohydrate needs. The presence of this bacterial-type enzyme illustrates that the adaptation of the plant parasite to their sojourn in their specific hosts may have been facilitated by HGT events. In agreement with previous studies on the carbohydrate metabolism of *Phytomonas* [91], [92], genome analysis revealed the presence of a complete set of glycolytic enzymes, the majority of which seem to be sequestered inside glycosomes, similar to other trypanosomatids. The existence of glycosomes in *Phytomonas*, previously demonstrated, was now confirmed by the presence of peroxisomal targeting signals at either the C- or N-termini of the encoded glycolytic enzymes as well as by the identification of a number of genes for peroxisome biogenesis proteins or so-called peroxins.

Besides the horizontal alpha,alpha-trehalose phosphorylase transfer event described here, other HGT events were previously described for other *Phytomonas* isolates. A zinc-containing alcohol dehydrogenase from a trypanosomatid isolated from the lactiferous plant *Euphorbia characias*, previously identified as an isopropanol dehydrogenase of bacterial origin, was also acquired by an event of lateral gene transfer from a strictly aerobic bacterium to an ancestral trypanosomatid [93]. The addition of this gene could explain a selective advantage for a plant colonizing-flagellate living in the phloemic or lactiferous tubes of infected plants, supported by the fact that this enzyme was only identified in all plant trypanosomes analyzed thus far, while absent from the rest of the trypanosomatid family. This zinc-containing alcohol dehydrogenase, together with a glycosomal malate dehydrogenase (Table S3), allowed us to assume that EM1 and HART1 would be able to produce small amounts of lactate, as observed for other *Phytomonas* isolates [94].

Almost nothing is known about the amino acid metabolism in *Phytomonas*. Amino acid metabolism of *Phytomonas* resembles that of the other trypanosomatids. The so-called non-essential amino acids can either be degraded and utilized as energy sources, or be formed from other metabolites. However, *Phytomonas* lacks the capacity to oxidize aromatic amino acids and is predicted to require an external supply of most of the essential amino acids. The absence of a fatty acid beta-oxidation pathway and of ETF predicts that *Phytomonas* is unable to oxidize both long chain and side chain amino acids (Results in Figure S21, for details see Table S3).

An arginine kinase was detected as a single copy gene in both isolates. This enzyme may have been acquired by horizontal gene transfer from the arthropod vector during evolution, as previously shown for *Phytomonas* Jma [95]. The genomes revealed that overall the interconversion and breakdown of amino acids is very similar to what has been described for the other trypanosomatids. However, while amino acids serve as the most important source of energy for the other trypanosomatids inside their insect vector, this cannot be the case in *Phytomonas* because of its limited mitochondrial capabilities [91]. Owing to the fact that their insect vector(s) feed exclusively on plant juices that are rich in carbohydrates, the switch from plant to insect host would probably not require a metabolic switch from carbohydrate to amino acid metabolism as occurs in the mammalian trypanosomes. The absence of such a switch may have allowed the irreversible loss of a number of mitochondrial functions such as a respiratory chain required for beta oxidation of fatty acids and the complete oxidation of amino acids. Indeed, no genes coding for any of the mitochondrial cytochromes could be found.

The enzymes of the hexose monophosphate pathway, as well as the ones involved in gluconeogenesis are present in *Phytomonas*, even though no evidence for the synthesis of glycogen has been detected. Few genes were found for the formation of storage polysaccharides. However, several mannosyl transferases, possibly involved in the synthesis of mannan polysaccharides, were detected, suggesting that mannans rather than glycogen may serve as a polysaccharide store.

Protein glycosylation differs in the two *Phytomonas* isolates (Figure S20, Table S3B). The genes required for the incorporation of glucose, mannose, galactose, N-acetylglucosamine, glucuronic acid, xylose and fucose into glycoproteins, but not for sialic acid, were identified in the genome of the EM1 isolate. The HART1 isolate seems to lack the genes necessary for the incorporation of N-acetylglucosamine and fucose.

With respect to lipid metabolism, fatty acyl dehydrogenase or, oxidase, multifunctional enzyme and thiolase were absent in both parasite isolates, indicating that *Phytomonas* is not capable of oxidizing any fatty acids via the beta oxidation pathway. On the other hand, *Phytomonas* should be capable of fatty acid biosynthesis, since the genes coding for the responsible enzymes have been identified in both parasite genomes (Type II fatty acid synthesis in mitochondrion, and Type I fatty acid synthesis absent but synthesis taking place by a set of elongases) (Figure 8).

Oxidant stress protection in trypanosomatids is based on trypanothione, an adduct of one spermidine and two molecules of glutathione [96]. Thus the *Phytomonas* proteome was searched for the presence of enzymes involved in this metabolism. *Phytomonas* has a trypanothione reductase as well as a homolog of glutathionylspermidine synthase, or trypanothione synthase, as well as the enzymes thioredoxin (tryparedoxin), several thioredoxin (tryparedoxin) peroxidases, peroxiredoxin, and trypanothione peroxidase. Several mitochondrial and cytosolic superoxide dismutases and an iron/ascorbate oxidoreductase, but no catalase, were identified. The reducing equivalents in the form of NADPH are provided by the enzymes NADP-dependent isocitrate dehydrogenase in the mitochondrion and by the hexose-monophosphate pathway enzymes glucose-6-phosphate dehydrogenase and 6-phosphogluconate dehydrogenase. A plant-like ascorbate peroxidase, as described for *T. cruzi* and *Leishmania*, was not detected (Table S3B).

*Phytomonas* lacks the capacity for RNAi, since the argonaut AGO1 (Tb10.406.0020) and the two dicer proteins DCL1 (Tb927.8.2370) and DCL2 (Tb927.3.1230) present in both *T. brucei* and in *L. brasiliensis* but not in *T. cruzi* and *L. major*, two organisms that lack RNAi, were also absent in both EM1 and HART1 genomes (see Table S3B). In fact, the lack of these gene products agrees with the presence of a double stranded RNA virus reported in the phloem-restricted isolates [97] that could serve as an indication for the absence of defense mechanisms against invasion by foreign RNA. Similar viruses have been reported for *Leishmania* spp. as well [98], [99].

### Analysis of the *Phytomonas* HART1 and EM1 secretome

Virtually no information is available about the existence of effectors of pathogenicity in *Phytomonas* spp. and their possible role in the interaction with the host. We investigated the secretome of *Phytomonas* EM1 and HART1 isolates for potential virulence factors, by selecting those sequences having a secretion signal peptide, no transmembrane domains and no glycophosphotidylinositol (GPI) anchors. We detected 282 putative secreted proteins in both HART1 and EM1 (Table S13). Among these proteins, only 43 proteins in HART1 and 44 in EM1 had a PFAM domain annotation. The secretome was classified into molecular function and biological process using the Gene Ontology annotation (Figure S22). However, we noted the presence of numerous false positives in the set of predicted secreted proteins. This is due to the high divergence between the trypanosomatid sequences and the one used by SignalP for learning, mostly from fungi, animals, plants or bacteria origin.

In the set of putative secreted proteins, we looked for proteins involved in plant carbohydrate degradation. One protein having a glycoside hydrolase family 31 domain was present in both HART1 and EM1 isolates, but the EST data did not show any expression of the two corresponding genes. We also found a secreted protein in HART1 (GSHART1T00001406001) coding for glycosyl hydrolase family 32 that corresponded to one of the beta-fructofuranosidases (see Metabolism of HART1 and EM1 section); other beta-fructofuranosidases harbored a signal peptide and GPI anchor. We did not identify any secreted proteins that were supported by expression data and likely to be involved in plant cell wall degradation. This finding is consistent with the fact that *Phytomonas* is directly injected in the host phloem by an insect vector, thus it does not need to degrade the plant cell wall to penetrate into the host and gain access to the phloem sap.

We screened for secreted proteins having a proteolytic activity that may lead to degradation of host proteins. Three genes were found in EM1 coding for an S24 serine peptidase, an M3A metallo-peptidase and an A1 aspartyl protease (AP); one AP was also found in the secretome of HART1. Cathepsin D-like A1 family AP genes have not been found in other known trypanosomatid genomes such as *Leishmania* and *Trypanosoma*. However, APs are known to be secreted and involved in the virulence of several pathogenic fungi. In the case of the fungal animal pathogen, *Candida albicans*, ten APs that contribute to the dissemination of the pathogen in mice are present [100]. Fourteen APs are also present in the genome of the ascomycete plant pathogen *Botritys cinerea*, including BCap8, which was found to constitute up to 23% of the total secreted proteins [101].

Since secreted *Leishmania* proteins with proteolytic activities may contribute to pathogenesis [102], [103], we looked for other AP coding genes in the HART1 and EM1 genome. EM1 did not have any extra APs, while HART1 harbors a cluster of five APs located in scaffold 1 (Table S3). These five tandem genes, absent in the syntenic region of EM1, were not detected by the “synteny” approach because of the stringency of filtering (see Material and Methods). The “true” first methionine of each protein of the cluster was located in intercontig gaps. When extending the N-terminal region of each of these proteins, a signal peptide could only be detected for the most extended gene (GSHART1T00000177001). For the four remaining APs, the N-terminal extension was not long enough to detect a probable signal peptide, and none of the five APs harbored a GPI anchor.

**Figure 9:**
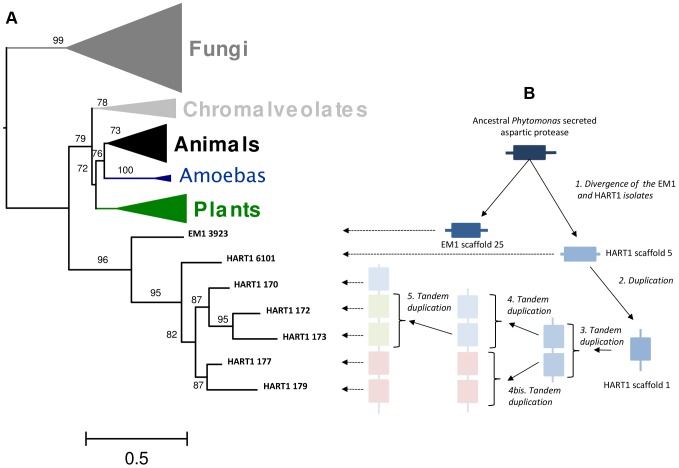
Duplication of ancestral *Phytomonas* secreted aspartyl protease sequences and maximum-likelyhood plylogenetic tree of aspartyl proteases. A. 89 amino acids ML phylogenetic unrooted tree built on the cured alignment of 64 aspartic proteases. The branch supports are approximate likehood-ratio test (aLRT) results. Clades corresponding to animals, fungi, chromalveolates, amoebas and plants are collapsed. The leaf labels EM1 3923, HART1 6101, HART1 170, HART1 172, HART1 173, HART1 177, HART1 179 correspond respectively to genes GSHART1T00006101001, GSHART1T00000170001, GSHART1T00000172001, GSHART1T00000173001, GSHART1T00000177001, GSHART1T00000179001. B Representation of the duplication and tandem duplications that created the cluster of five APs in the HART1 scaffold 1.

The phylogenetic analysis (Figure 9A) revealed that these *Phytomonas* APs evolved from a common gene that branched deeply in the tree with high aLRT support (aLRT support = 96). This result suggested the existence of an ancestral AP gene in the trypanosomatid lineages that may have been lost in *Leishmania* and *Trypanosoma*. The integration of the APs genomic positions on scaffold 1 and the topology of the HART1 clade allowed the reconstruction of the events that led to the creation of a pathogenicity gene cluster in HART1 (Figure 9B). HART1 and EM1 had initially one copy of the gene coding for a secreted AP. Then, the HART1 gene duplicated once from scaffold 5 to scaffold 1. The cluster of five genes was created in the scaffold 1 of HART1 by four successive tandem duplications. The presence of a signal peptide in the AP from EM1, the AP in scaffold 5 and one AP in the cluster of scaffold 1 let us speculate about the presence of a signal peptide in the other four APs, but their sequences were too short to detect it. The scaffold gaps between the five AP genes may correspond to repeated elements that may have mediated the AP tandem gene duplication. The EST data provided evidence for the expression of the five AP genes which comprise the AP cluster in the *Phytomonas* HART1 isolate (Figure S23), suggesting that, similarly to the function of the AP family in the fungi *Candida* and *Bothrytis* [101], [104], the *Phytomonas* HART1 AP gene cluster could be involved in virulence, an example of convergent evolution between distant organisms.

### Pathogen versus non-symptomatic genomes: Examples and possible biological implications

The genus *Phytomonas* encompasses flagellates that differ substantially in their pathogenic potential. Despite most genes being shared between EM1 and HART1 isolates with respect to both gene count and content, several differences are still present (Table S3, Table S9). Among the members of the *Phytomonas* EM1 and HART1 kinase repertoires, only two specific genes were identified: a CMGC/DYRK EM1-specific kinase, absent from the HART1 genome, and an AGC/RSK only present in the pathogenic HART1 isolate. Both specific kinases have no orthologs in *T. brucei* or *L. major*. Since the function of these kinases has not been studied in any trypanosomatid, and little is known about protein kinase signaling pathways in the TriTryps, the biological implications are not clear at present. While two CDC25 phosphatases were also identified in EM1, no orthologs were found in HART1, as in the case of *T. brucei*. The CDC25/CDD25-like phosphatases were identified in *Leishmania* spp. and *T. cruzi*, suggesting distinct roles for the protein phosphatases only present in the two intracellular trypanosomatids.

*Phytomonas* is adapted specifically to infect and live in plants, where an abundant and diverse supply of carbohydrates is available for the parasite. Surprisingly, genomes of both EM1 and HART1 isolates contained only one sugar transporter, an ORF encoding a GT2 homolog (Table S3E). The presence of only a single sugar transporter is intriguing. It would suggest that both EM1GT2 and HART1GT2 have a broader substrate specificity than the mammalian trypanosome GT2, and would be in agreement with a much more simplified metabolic life cycle.

The pathogenic HART1 isolate seems to be specialized in metabolizing sucrose, as is suggested by the presence of many copies of an invertase (fructofuranosidase) homolog only detected in this strain. The main difference between the pathogenic and asymptomatic *Phytomonas* isolates resides in their specific location inside the host: EM1 multiplies in latex tubes, while HART1 colonizes the phloem sap inside the sieve tubes. It is not yet clear whether this difference in habitat is related to the presence, or not, of multiple invertase genes. The presence of an alpha, alpha-trehalose phosphorylase in *Phytomonas* may be the explanation of why this plant parasite can survive in the insect hemolymph by using trehalose, a disaccharide of glucose, as an energy substrate, rather than amino acids, as is the case in midgut-dwelling trypanosomatids of hematophagous insects. Trehalose, originally regarded as a sugar characteristic of certain lower plants, is also a major blood sugar of insects [105]. Since the phosphorylase is present in all trypanosomatids for which the genome has been sequenced so far, it is unlikely that the enzyme would be involved in the parasite's energy metabolism when dwelling in the plant host. It is more likely that it fulfills a major role in the passage of trypanosomatids through their insect vector rather than to their survival in the widely different types of mammalian and plant hosts, where only some plants have high concentrations of trehalose.

Both isolates seem to differ in the make-up of their surface glycoproteins (examples in the Text S1 and Table S3D). Most significantly, HART1 and EM1 have radically distinct gp63 repertoires, with only two genes detected in EM1 but over 20 in HART1. These are clearly derived from a single common precursor, with multiple expansions in HART1, and suggesting a more complex surface for HART1 than EM1 that potentially facilitated adaptation to a greater range of conditions, host species or tissue spaces.

Perhaps the pathogenic effects of HART1 are primarily due to their location in the sap, containing the products of photosynthesis and essential for plant growth. The death of palms, coffee trees and *Alpinia* may be the result of competition for essential metabolites that are more efficiently scavenged by *Phytomonas*. Biological inoculation experiments using the isolate EM1 in palms would address this hypothesis. The specific relationships among *Phytomonas*, its vector, and the host make this experiment hard to endeavour. The intraphloemic trypanosomatids associated with wilts form a very distinct group, especially for their cultivation [15]. Parasites could not be isolated without the help of feeder cells in the cultures, while the cultures of latex isolates like EM1 or fruit isolates were obtained in an axenic medium. Further comparative analysis of the two *Phytomonas* genomes may reveal the source of these differences.

### *Phytomonas* genome: A minimized gene repertoire as a hint for survival in the plant host

The *Phytomonas* genomes consist essentially of single copy genes for the bulk of their metabolic enzymes, whereas *Leishmania* and *Trypanosoma* possess numerous duplicated genes or large gene families. While such gene duplications may have helped some trypanosomatids to adapt to multiple, widely different hosts, i.e. poikilothermic insects and warm-blooded mammals, their absence in the two *Phytomonas* genomes analyzed here suggests that plant trypanosomatids have been confronted much less with strikingly different metabolic environments and temperatures, and have hence lost or never needed these additional paralogs.

The unlimited availability of sugars in both plant and insect hosts is a situation that normally leads to suppression of mitochondrial activities, such as cyanide-sensitive respiration and oxidative phosphorylation. Eventually this may result in an irreversible loss of the genes coding for all of the above functions. The irreversible suppression observed in *Phytomonas* resembles the adaptation of some African trypanosomes to a permanent stay in the bloodstream of their mammalian hosts, without the possibility for cyclic transmission via insects. This also leads to the loss of mitochondrial genes and results in the appearance of dyskinetoplastic or akinetoplastic trypanosomes, unable to survive in the tsetse fly. Interestingly, *Phytomonas* spp. possess orthologs to the mitochondrial calcium uniporter recently described [106] suggesting that, as bloodstream forms of *T. brucei*, they also utilize the mitochondrial ATPase in reverse to maintain a membrane potential that drives Ca^2+^ uptake through the uniporter. Some *Phytomonas* genes likely gained via HGT may have permitted increased flexibility of genome expression, enabling the successful adaptation of *Phytomonas* spp.

Significant genome reduction has been identified in microbial lineages living in selective environments. Selection plays a key role during the initial phases of such adaptation removing “accessory” genes [107]. High gene density bear witness of genome contraction in several obligate intracellular parasites. In the case of microsporidia, genome-size variation resulted from varying frequencies of repeat elements without affecting gene density. Furthermore, *Phytomonas* shows important host dependency pictured by considerable gene losses. These adaptations combined with genome compaction led to gene size reduction and simplification of certain cellular processes [108].

Also phytoplasmas, specialized bacteria living as obligate parasites of plant phloem tissue and transmitting insects [109], have suffered extreme genome shrinkage, which resulted in a gene repertoire that is specific for survival in plant hosts [110]. In the case of phytoplasmas, this adaptation was made possible thanks to the presence of repeated DNAs, which allowed survival in different environments. Here also adaptation is particularly important, as their host environments, including phloem tissues of plants, and guts, salivary glands, and other organs and tissues of the insect host, are extremely variable [110].

*Phytomonas* spp. are highly specialized trypanosomes, with central differences in life history and infection strategy compared to eukaryotic plant pathogens like fungi and oomycetes. Leaf, fruit and stem are some of the surfaces colonized by plant pathogens. Wind-blown rain, fog and any plant visitor are some of the mechanisms by which phytopathogens like filamentous fungi and oomycetes are disseminated to the host plants. Still, these phytopathogens should penetrate by themselves in order to colonize and circulate inside the host. In this process, several biological mechanisms are triggered to colonize and propagate into the host, by the use of enzymes (cutinase, cellulase, pectinase), hormones, toxins and frequently by the interaction with metabolites produced by the plant in response to the invasion [111].

Contrary to these phytopathogens, plant trypanosomatids do not need to degrade cell walls to settle inside the plant since they are deposited into very specialized tissues or cells in the host thanks to insects that acts as their natural carriers. Yet, the discovery of a *Phytomonas* HART1 AP gene cluster, known to be secreted and involved in the virulence of several pathogenic fungi [100], [101] but missing in animal parasitic trypanosomids and the non pathogenous EM1 isolate, could be described as a good example of convergent evolution between these distant phytopathogen organisms.

The genome completion is the first step towards development of effective chemical control agents against *Phytomonas* spp., which is not only of economic interest, but may have relevance for other Trypanosomatidae pathogenic to humans and animals, since they share similar metabolic routes and many other biological mechanisms [112], [113]. Comparative studies between plant, human and animal pathogenic trypanosomatids as well as free living species will assist in the identification of gene cohorts specifically linked to various pathogenesis mechanisms. These comparisons will also contribute towards better and safer control methods for trypanosomatid diseases of animals, plants and humans and provide better insights into the evolution of parasitic and pathogenic mechanisms.

## Materials and Methods

### Genome sequencing and assembly

The sequencing strategy used for both *Phytomonas* genomes corresponds to a mix of three technologies: 454/Roche for most coverage; Solexa/Illumina for automatic corrections of low-quality regions (especially around homopolymers); and classical Sanger sequencing at low coverage with large-insert clones (10 kb-insert containing plasmids and fosmids) to organize the contigs into scaffolds. The assembly method is described in detail in Text S1.

### Chromosome read depth analysis

Illumina reads were mapped to the corresponding *Phytomonas* genome using SOAP version 1.10 [114], under the guidance of a custom perl script. The number of bases mapping to each position in each scaffold was recorded, and used to determine the total number of read bases mapping to each scaffold and the median read depth for each scaffold. Observing that a majority of the scaffolds displayed similar median read depths, and interpreting this as a nominal ‘ploidy’, a within-genome normalisation was performed by setting the average of the read depth of the four longest ‘euploidic’ scaffolds to 2. The read depth for each scaffold was subsequently normalized to this value. Results of the scaffolds “somy” are shown in Table S1.

### Genome annotation

Protein-coding genes are predicted by combining *ab initio* gene model predictions (already trained on manually annotated genes) and homology searches, using collections of expressed sequences - full length cDNAs, ESTs or massive-scale mRNA sequences from the same or closely related organisms – proteins or other genomic sequences. Details on the pipeline are given in the Text S1. Moreover, tRNA-Scan [115] was used to detect tRNAs in both *Phytomonas* assembled sequences.

After a final integration of all gene evidence using GAZE [116], the final proteome was delivered with computed annotation data, such as ortholog and paralog associations, functional domains and ontology relationships.

### Identification of candidate horizontal gene transfers

*Phytomonas* proteins were used against the protein nr database (blastx, [117]), with the parameters “-f 100 -X 100 -e 0.00001 -E 2 -W 5”, and the best hits were retained using the following criteria: only BLAST scores greater than 90% of the best score outside kinetoplastids (so that horizontal gene transfers shared between kinetoplastids could be detected) and above 100 were retained. Then, the proteins with all their best hits in bacteria or archaea were retained as candidates to have arisen from bacterial/archeal horizontal gene transfers. All the candidates were then manually inspected, which provided 87 final candidate HGT genes, 80 of which have orthologs in other trypanosomatids, and 8 have no orthologs in *Leishmania* spp. nor *Trypanosoma* spp. and might thus be specific of *Phytomonas* (Table S4).

### Detection of orthologs between EM1, HART1 and other trypanosomatids

We identified orthologous genes between *Phytomonas* EM1 and HART1, and 4 other trypanosomatids: *L. major* [29], *T. brucei* [28], *T. cruzi* [30] and *T. vivax* [70] (Tritryp release 2.1). Each pair of annotated genes was aligned with the Smith-Waterman algorithm, and alignments with a score higher than 300 (BLOSUM62, gapo = 10, gape = 1) were retained. Orthologs were defined as best reciprocal hits (BRH), i.e. two genes, A from genome GA and B from genome GB, were considered orthologs if B is the best match for gene A in GB and A is the best match for B in GA. Indeed, 5006 gene pairs (representing 77.6% for HART1 and 78.4% for EM1 genes), similar in gene size and intergenic length, were detected using this approach (Figure S9A and B). The number of BRH for each comparison and their average and median %id are displayed in Figure 3; the distribution of %id for these BRH between different pairs of species designated both *Phytomonas* isolates as being much closer to each other than to other trypanosomes (average %id of 70% for EM1 and HART1; 56 to 57.6% between the different pairs of trypanosomes).

The results of the pairwise alignments between all protein sequences of the 6 genomes were then inputted to the orthoMCL software V1.4 [69], in order to assemble clusters of orthologous genes between both *Phytomonas* EM1 and HART1, and other trypanosomatids. This approach was complementary to what was observed by the BRH strategy, since it permitted us to ascertain orthologs for multigenic families, not seen by the BRH strategy alone. This procedure provided 7,694 clusters of orthologs genes, gathering 5,188 EM1 and 4,643 HART1 genes in clusters containing genes from both isolates (regardless of the presence or absence of genes from other trypanosomes) (Table 1).

We also ran orthoMCL on the subset of genes from EM1 and HART1 that have strong support (i.e that are overlapping uniprot genewise hits, or cDNA reads as well as *ab initio* predictions): EM1 contains 5,237 such genes, and HART1 5,247 genes (Table 1).

### Identification of genes absent from one isolate compared to the other

#### 1/“homology” approach

After combining BRH and orthoMCL approaches, 1,171 EM1 genes remained with no ortholog detected in HART1, and 1,343 HART1 genes remained with no ortholog detected in EM1. But the fact that no orthologous gene could be detected was likely due to detection issues rather than differences between the isolates. In order to filter out those detection issues, we first aligned the protein sequences against the genome, with the same pipeline as the one used for aligning uniprot proteins, *i.e.* using BLAT [118] followed by genewise [119]; 504 genes from EM1 and 602 genes from HART1 displayed a genomic match in the other isolate, which correspond to missed genes or wrong annotations (Figure S10). The genes that displayed no match on the genome were subsequently aligned against the reads using tblastn [120] with an e-value cutoff of 10e^−04^. Genes matching on at least 5 reads, and covered on > = 25% of their length with an average %id > = 35% were retained: 247 genes from EM1 and 359 genes from HART1 are present in the reads but not in the assembly. Among the remaining genes, a substantial proportion was suspected to correspond to annotation artefacts. They were filtered out by retaining only genes overlapping uniprot genewise hits or cDNA reads and *ab initio* predictions, or genes sharing homology with other trypanosomatids (*i.e.* present in orthoMCL clusters). 82 genes remained in EM1 and 83 genes in HART1. We then investigated the syntenic regions of those genes and discarded the genes for which we could not find a syntenic region (genes upstream and downstream were not in the same operon), and for which there were intercontig gaps in the intergenic region; such genes were likely not assembled but possibly present in the genome (and not detected by the mapping on the reads because of the stringency of the criteria). Finally, manual curation of the remaining candidates allowed elimination of doubtful candidates (with doubtful structures or some homology with the syntenic region). This procedure provided 13 EM1 genes with no ortholog in HART1 and 4 HART1 genes with no ortholog in EM1 (Figure S10).

#### 2/“synteny” approach

We also investigated genes from each isolate that had no counterpart in the syntenic region of the other isolate. We first identified pairs of successive BRH between EM1 and HART1 that were in the same operon and harbored different numbers of genes between them. They were filtered to eliminate structural differences of annotation (splits/fusions) between the two isolates: if the two orthologous genes of at least one BRH pair surrounding the region differed in length by more than 500 bp, the region was discarded. Discrepancies likely due to missed genes in the annotation were also filtered out: we only retained syntenic regions with lengths differing by more than 1000 bp between the two *Phytomonas* isolates. Regions containing intercontig gaps were also discarded. Finally, all candidates were inspected manually, and aligned using blastn [121] to the genome of the other isolate in order to sort out cases where the gene was absent from the whole genome and cases where the gene was absent in synteny but present somewhere else in the genome. The approach was sensitive enough to detect gene order changes (gene inversions) as well as translocations: the 7 EM1 genes and 5 HART1 genes that were detected in another position on the genome of the other isolate are described in Table S10. We detected 10 genes in EM1 that are neither present in the syntenic position in HART1 nor anywhere else (3 of which were already identified as having no ortholog), and 3 genes in HART1 that are not present in the syntenic position in EM1 nor anywhere else (2 of which were already identified as having no ortholog): Table S9. The “synteny” approach retained genes with weak homology with other genes anywhere on the genome (because they share a common domain for instance) that had been discarded by the alignment on the genome/reads in the “orthology” approach. On the other hand, some of the genes detected by the “orthology” approach were not detected by the “synteny” approach because the very stringent gene structure filter from the “synteny” approach discarded some genes identified by the “orthology” approach.

Combining the two complementary approaches, 20 genes from EM1 and 13 genes from HART1 are identified. Since the two approaches are not capturing the same genes, we suspect that more genes specific of each isolate have been missed in the automatic detection process, but our aim was to be conservative and keep only cases with strong support. Refining the selection would require extensive manual curation.

### Coincidence of synteny breaks with operon boundaries

An in-house perl script was used to draw the dot plots and build syntenic blocks between species. The clustering was performed by single linkage clustering using the euclidian distance between genes. Those distances were calculated with the gene index in each scaffold rather than the genomic position. The minimal distance between two orthologous genes was set to 10 on both counterparts and we only retained clusters that were composed of at least 5 pairs of paralogous genes. The boundaries of the syntenic clusters were then filtered in order to eliminate those occuring at the end of scaffolds and corresponding to “assembly breaks” rather than synteny breaks. As a consequence, for genomes with a more fragmented assembly, the number of synteny breaks detected is lower because some real syntenic breaks occur at scaffolds boundaries and are discarded. This is the case for *T. cruzi* (41 scaffolds) that appears to have less synteny breaks with *Phytomonas* compared to *T. vivax* and *T. brucei* (11 chromosomes). We performed a simulation to distribute randomly the same number of synteny breaks as observed for each scaffold (1000 iterations) and counted the number of randomly distributed synteny breaks that coincided with operon boundaries. In all cases, the observed number of synteny breaks at operon boundaries was significantly higher than expected randomly (Figure S12B).

### Search for specific gene families in *Phytomonas*

Both genomic EM1 and HART1 assemblies were queried using sequence probes encompassing selected Interpro domains, by a series of reciprocal sequence comparisons using the BLAST server, accessed through the SeqTryplant Genome Browser or directly on a secure web site. Likewise, the reads not included in the assembly as well as the contigs smaller than 5 kb and so excluded from the assembled sequence, were scanned with the same probes. The results obtained were subsequently examined by the experts of the *Phytomonas* consortia in order to validate the gene models. Details on the probes and procedure used for each gene family can be found in the Text S1 file.

### Comparison of trypanosome gene repertoires

The same gene probes used to search for gene families in both *Phytomonas* genomes were later employed to query the TriTrypDB 4.0 Released, in order to obtain the corresponding genes in the *T. brucei*, *T. cruzi* and *L. major* genome annotations. Later on, these sequences were applied to query the OrthoMCL DB (version 5), and copy number, as automatically defined by the OrthoMCL approach was reported. Moreover, *T. brucei*, *T. cruzi* and/or *Leishmania* spp. gene copy number for members of certain families (e.g. kinases and transporters) was obtained from the literature or human expertise when available (Table 2 and Table S3).

### Analysis of the *Phytomonas* secretome

Proteins with a signal peptide were detected with SignalP version 3.0 [122], transmembrane domains were detected with TMHMM 2.0 [123] and GPI anchors with KOHGPI version 1.5 (http://gpi.unibe.ch/). Proteins harboring a signal peptide, not containing transmembrane domains nor GPI anchors were considered to be secreted by *Phytomonas*, and their annotation was performed using BLASTp against the non-redundant NCBI database, Interproscan and Gene Ontology. We retrieved aspartic proteases from others clades (amoebae, plants, chromalveolates, fungi and animals) using the *Phytomonas* aspartic proteases amino acid sequences as queries with BLASTp on the NCBI nr database [121]. Phylogenetic analysis was executed on the Phylogeny.fr platform [124] as described in [125], with the parameters “minimum length of a block after gap cleaning: 5, no gap positions were allowed in the final alignment, all segments with contiguous non conserved positions bigger than 8 were rejected, minimum number of sequences for a flank position: 85%” for Gblocks v0.91b [126].

## Supporting Information

Figure S1 Chromosome copy number variation in *Phytomonas* genomes. Read depth was scaled to give a value of 2 for disomic scaffolds. Median read depth over all scaffolds in the genome is indicated in brackets. (A) EM1 (30); (B) HART1 (50).
(TIF)

Figure S2 Distribution of allele frequencies of heterozygous single nucleotide polymorphisms (SNPs) across *Phytomonas* EM1 and HART1 genomes. Y-axis corresponds to allele count; X-axis shows allele frequencies of heterozygous SNPs. A. *Phytomonas* EM1 whole genome; B. *Phytomonas* HART1 whole genome.
(TIF)

Figure S3 Distribution of allele frequencies according to inferred ploidy for *Phytomonas* EM1 scaffolds. A, EM1 chromosomes with 2 copies (74 scaffolds and 3,608 SNPs); B, EM1 chromosomes with 3 copies (14 scaffolds and 816 SNPs); C, EM1 chromosomes with 4 copies (3 scaffolds and 84 SNPs); D, EM1 chromosomes with 5 copies (1 scaffold and 26 SNPs) and E, EM1 chromosomes with 6 copies (2 scaffolds and 116 SNPs).
(TIF)

Figure S4 Distribution of allele frequencies according to inferred ploidy for *Phytomonas* HART1 scaffolds. A, HART1 chromosomes with 2 copies (56 scaffolds and 8,774 SNPs); B, HART1 chromosomes with 3 copies (15 scaffolds and 828 SNPs) and C, HART1 chromosomes with 4 copies (2 scaffolds and 264 SNPs).
(TIF)

Figure S5 Distribution of read depth along *Phytomonas* EM1 disomic and tetrasomic scaffolds. Y-axis corresponds to read depth; X-axis shows scaffold length plotted on a log scale. A. *Phytomonas* EM1 Scaffold_1 (disomic); B. *Phytomonas* EM1 Scaffold_24 (tetrasomic).
(TIF)

Figure S6 Identification of polycistronic gene clusters (PTUs) in *Phytomonas*. A. Strategy used for PTUs detection in *Phytomonas* EM1 and HART1 genomes (details in Text S1). B. Statistics on *Phytomonas* PTUs.
(TIF)

Figure S7 Conservation of tRNA synteny within kinetoplastid genomes. A. Conserved clusters of tRNAs found in HART1 (scaffold 1) and the corresponding scaffolds from EM1. ‘-’ represents tRNA genes absent from one scaffold. B. Partial synteny of tRNA genes between HART1 (scaffold 4) and EM1 (scaffold 45). C. Synteny of tRNA genes associated transcriptionally with other small-RNA genes, U3 and 7SL, in *Leishmania major*. ‘?’ represents a hypothetical RNA pol III promoter for the downstream 7SL RNA gene. The figure is not drawn to scale.
(TIF)

Figure S8 *Phytomonas* HART1 maxicircle.
(PDF)

Figure S9 Gene size and intergenic length in *Phytomonas* EM1 and HART1. Correlation of gene size (A; from 5,006 pairs of BRH) and intergenic length (B; from 3,504 orthologous intergenic regions intra operons - pairs of adjacent orthologous genes - between *Phytomonas* EM1 and HART1.
(TIF)

Figure S10 Flowchart of the strategy followed to purify the list of *Phytomonas* genes with no ortholog in the other isolate. A: EM1 genes with no ortholog in HART1; B: HART1 genes with no ortholog in EM1.
(TIF)

Figure S11 Synteny between *Phytomonas* EM1 and HART1. Dot plot representation, with PTUs colored. A. EM1 PTUs colored, B. HART1 PTUs colored. Different colors in the diagonal lines mean that the synteny blocks contain several PTUs.
(TIF)

Figure S12 Relationships between *Phytomonas* EM1 and HART1 PTU's genes. A. Orthologous relationships between *Phytomonas* EM1 and HART1 genes. PTUs are represented by different arbitrary colors so that PTU boundaries can be visualized. Relationships between orthologous genes are painted with the color of EM1 PTUs in order to facilitate the visualization of operon boundaries conservation B. Synteny breaks between *Phytomonas* EM1 and HART1 and human trypanosomes. Pairwaise comparison between EM1 and HART1 isolates and kinetoplasitds *T. brucei* (Tb), *T. cruzi* (Tc), *T. vivax* (Tv) and *L. major* (Lm). P-val: P-value (probability of an observed result arising by chance).
(TIF)

Figure S13 Comparison of PTUs and synteny blocks: example of *Phytomonas* EM1 scaffolds 1 and 2. For each scaffold, the first line shows the PTUs in different arbitrary colors (changes in colors correspond to PTU boundaries) and the next 5 lines represent the syntenic blocks with 5 other species (each syntenic block is represented by a different arbitrary color: changes in colors correspond to synteny breaks): *Phytomonas* HART1 (HART1), *L. major* (Lm), *T. brucei* (Tb), *T. cruzi* (Tc), *T. vivax* (Tv).
(TIF)

Figure S14 Synteny between *Phytomonas* EM1/HART1 and *Leishmania major* (Lm). Dot plot representation of BRH between EM1 and Lm (A; 4,607 genes), and HART1 and Lm (B; 4,322 genes). Each dot represents a pair of genes (BRH), with the position of the EM1/HART1 gene on the EM1/HART1 assembly on the x axis, and the position of the Lm gene on the Lm assembly on the y axis. Genes (dots) are colored according to the EM1/HART1 PTU they belong to.
(TIF)

Figure S15 Synteny between *Phytomonas* EM1/HART1 and *Trypanosoma brucei* (Tb). Dot plot representation of BRH between EM1 and Tb (A; 4,014 genes) and HART1 and Tb (B; 3,806 genes). Each dot represents a pair of genes (BRH), with on the x axis the position of the EM1/HART1 gene on the EM1/HART1 assembly, and on the y axis the position of the Tb gene on the Tb assembly. Genes (dots) are colored according to the EM1/HART1 PTU they belong to.
(TIF)

Figure S16 Synteny between *Phytomonas* EM1/HART1 and *Trypanosoma cru*zi (Tc). Dot plot representation of BRH between EM1 and Tc (A; 3,646 genes) and HART1 and Tc (B; 3,438 genes). Each dot represents a pair of genes (BRH), with on the x axis the position of the EM1/HART1 gene on the EM1/HART1 assembly, and on the y axis the position of the Tc gene on the Tc assembly. Genes (dots) are colored according to the EM1/HART1 PTU they belong to.
(TIF)

Figure S17 Synteny between *Phytomonas* EM1/HART1 and *Trypanosoma vivax* (Tv). Dot plot representation of BRH between EM1 and Tv (A; 3,822 genes) and HART1 and Tv (B; 3,631 genes). Each dot represents a pair of genes (BRH), with on the x axis the position of the EM1/HART1 gene on the EM1/HART1 assembly, and on the y axis the position of the Tv gene on the Tv assembly. Genes (dots) are colored according to the EM1/HART1 PTU they belong to.
(TIF)

Figure S18 Phylogenetic analysis of global lysine transporters. Radial phylogenetic tree of amino acid transporter proteins, including AAPs from *Phytomonas* EM1 and HART1, and mammalian trypanosomatids. Trypanosomatid transporter sequences with the indicated ID numbers were taken from GeneDB (http://www.genedb.org). Colors indicate different genera; *Leishmania* in blue, Trypanosomes in red and *Phytomonas* in black.
(TIF)

Figure S19 Phylogenetic reconstruction of gp63 families in *Phytomonas*. The predicted protein sequences of gp63 orthologs were retrieved from the EM1 and HART1 databases using BLAST, and analysed using MrBayes and PhyML.
(PDF)

Figure S20 Protein glycosylation in *Phytomonas*. Steps in the formation of the activated sugar residues for the glycosylation of proteins. Abbreviations: Glc, glucose; GlcN, glucosamine; GlcNAc, N-acetyl glucosamine; UDP, uridylyldiphosphate; Gal, galactose; Fru, fructose; Man, mannose; GDP, guanidyldiphosphate; Glr, glucuronic acid. Enzymes: 1, Glucokinase/hexokinase; 2,glucosamine-6-phosphate deaminase; 3, phosphoglucosamine mutase; 4 and 4a, bifunctional enzyme: glucosamine-1-phosphate acetyltransferase/UDP-N-acetylglucosamine pyrophosphorylase; 5, phosphoglucomutase; 6, UDP-galactose/glucose pyrophosphorylase (2.7.7.64); 7, UDP-glucose 4-epimerase; 8, Mannos-6-phosphate isomerase; 9, phosphomannomutase; 10, mannose-1-phosphate guanyltransferase/GDP-D-mannose pyrophosphorylase; 11, GDP-mannose 4,6-dehydratase; 12, GDP-L-fucose synthase; 13, UDP-glucose 6-dehydrogenase; 14, UDP-glucuronic acid decarboxylase; 15, galactokinase; 16, fucose kinase; 17, Fucose-1-phosphate guanylyltransferase.
(TIF)

Figure S21 Amino acid and dithiol metabolism in *Phytomonas* EM1 and HART1 isolates.
(TIF)

Figure S22 Gene Ontology (GO) Classification of the putative secretome of *Phytomonas* HART1 and EM1. The y-axis indicates the number of putative secreted protein sequences found under each GO term; x-axis corresponds to the GO classification of the molecular function (panel A) and GO classification of the biological process (panel B). AKI corresponds to *Phytomonas* HART1, AKH corresponds to *Phytomonas* EM1.
(TIF)

Figure S23 Genome browser view of the aspartic protease cluster. The upper part of the figure is a view of the EM1 genomic region lacking the cluster. The lower part represents the HART1 genomic region of the aspartyl protease cluster. Dotted lines indicate the synteny of the two regions.
(TIF)

Table S1 Scaffold somy calls in *Phytomonas* EM1 and Hart1 isolates. Median read depth coverage was computed for each scaffold across the whole EM1 and HART1 assemblies, and normalized by setting the average of the read depth to 2 (details of the procedure used can be found in Materials and Methods). Scaffolds bigger than 100 kb (scaffolds above the red line) highlighted in yellow are supernumerary.
(DOC)

Table S2 Resources used for *Phytomonas* EM1 and HART1 genome annotation.
(DOC)

Table S3 Expert curation of Phytomonas EM1 and HART1 gene families. *Phytomonas* EM1 and HART1 assemblies were queried using sequence probes encompasing selected Interpro domains, either a kinetoplastida counterpart (in bold), or an ortholog from other species (*). Afterwards, the results obtained were inspected by the *Phytomonas* consortia experts for confirmation. Details on the probes and procedure used for each gene family can be found in the Text S1 file. A, Ca2+ exchange; B, Metabolism; C, Protein kinases; D Intracellular trafficking factors; E, Transporters. Both yellow cells (corresponding to non-annotated regions with a match to the gene probe) and pink cells (unassembled regions - reads or contigs smaller than 500 bp - matching a gene probe) are highlighted. Notes: no orthoMCL, no orthoMCL cluster found; not identified, genes not found in *Phytomonas* EM1 and/or HART1 isolate. #, absent or existing but not used. a one single gene (split in the annotation). ## rBLASTs to Tritryps DB only.
(XLS)

Table S4 Horizontal gene transfer candidates specific of *Phytomonas* EM1 and HART1. *T. brucei* (Tb); *T. cruzi* (Tc), *L. major* (Lm). *, described in [52].
(XLS)

Table S5 *Phytomonas* EM1 and HART1 tRNA genes. tRNA genes were predicted in both *Phytomonas* isolates using tRNA-Scan. Green highlight indicates tRNA genes unique to *Phytomonas*. Yellow highlight indicates tRNA genes absent from all kinetoplastids. Orange highlight indicates tRNA genes found in other kinetoplastids but absent from *Phytomonas*.
(XLS)

Table S6 Percent identity at the nucleotide or protein level between maxicircle genes, calculated for alignments with gaps removed. vs. = versus; F = forward; R = reverse. *, percent identity for the gene calculated at the protein level; **, 60.8% at the nucleotide level, MURF5 gene was not included in the average percent calculation; ***, vs. *Leishmania amazonensis*.
(XLS)

Table S7 Transposable elements in EM1 and HART1 *Phytomonas* genomes. a, number of amino acids contained in the multifunctional protein encoded by the consensus sequence of autonomous and active retroposons; b, autonomous retroposons (“Auto”) potentially code for a protein responsible for their retrotransposition. Retroposons are considered active when bioinformatics analyses suggest recent retrotransposition events for most of the elements in the family; c, copy number per haploid genome.; d, not determined due to the high sequence heterogeneity; e, non-coding retroposons; f, the copy number of each retroposon in the 41.8 Mb dataset (the size of the haploid genome is not known); g, the copy number of each retroposon in the 47.7 Mb dataset (the size of the haploid genome is not known); h, number of copies in the assembled sequences.
(DOC)

Table S8 Conservation and taxonomic distribution of *Phytomonas* isolate snoRNAs.
(DOC)

Table S9 Genes absent from *Phytomonas* EM1 or HART1 genomes. *Trypanosoma brucei* (Tb), *Trypanosoma cruzi* (Tc), *Trypanosoma vivax* (Tv), *Leishmania major* (Lm).
(XLS)

Table S10 Genes from EM1/Hart1 absent in the syntenic region of *Phytomonas* HART1/EM1, but present elsewhere on the other isolate's genome.
(XLS)

Table S11 Protein Tyrosine Phosphatase (PTP) and Serine/Threonine specific protein phosphatase (STP) families in *Phytomonas* EM1 and HART1, compared to the TriTryp phosphatomes. A, Protein Tyrosine Phosphatase (PTP) families in *Phytomonas* EM1 and HART1; B, Serine/Threonine specific protein phosphatases (STP) in *Phytomonas* EM1 and HART1. *Trypanosoma brucei* (*T. brucei*), *Trypanosoma cruzi* (*T. cruzi*), *Leishmania major* (*L. major*), *Phytomonas* HART1 (HART1) and *Phytomonas* EM1 (EM1).
(XLS)

Table S12 ABC transporters in *Phytomonas* EM1 and HART1.
(XLS)

Table S13 *Phytomonas* EM1 and HART1 secretome.
(XLS)

Text S1 Supplementary information. A detailed description of methods used for the sequencing and annotation of *Phytomonas* EM1 and HART1 genomes and the manual inspection of selected *Phytomonas* gene families.
(DOC)
