# Supplementary figures and images for: The Streamlined Genome of Phytomonas spp. Relative to Human Pathogenic Kinetoplastids Reveals a Parasite Tailored for Plants
Source: PLoS Genet. 2014 Feb 6;10(2):e1004007. doi: 10.1371/journal.pgen.1004007 (PMC3916237; doi:10.1371/journal.pgen.1004007)

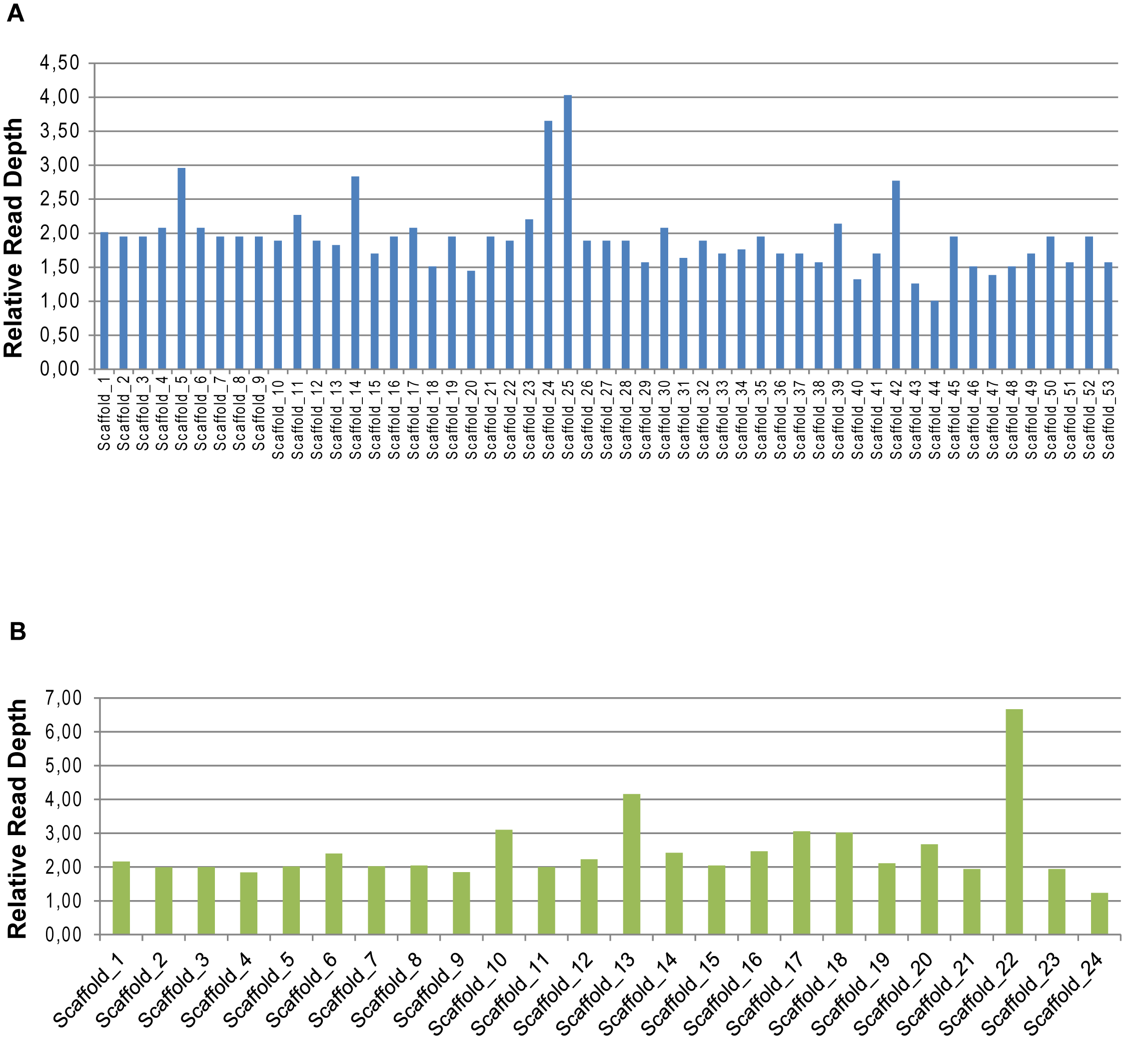

Supplement: Figure S1 — Chromosome copy number variation in Phytomonas genomes. Read depth was scaled to give a value of 2 for disomic scaffolds. Median read depth over all scaffolds in the genome is indicated in brackets. (A) EM1 (30); (B) HART1 (50). (TIF) [file pgen.1004007.s001.tif]

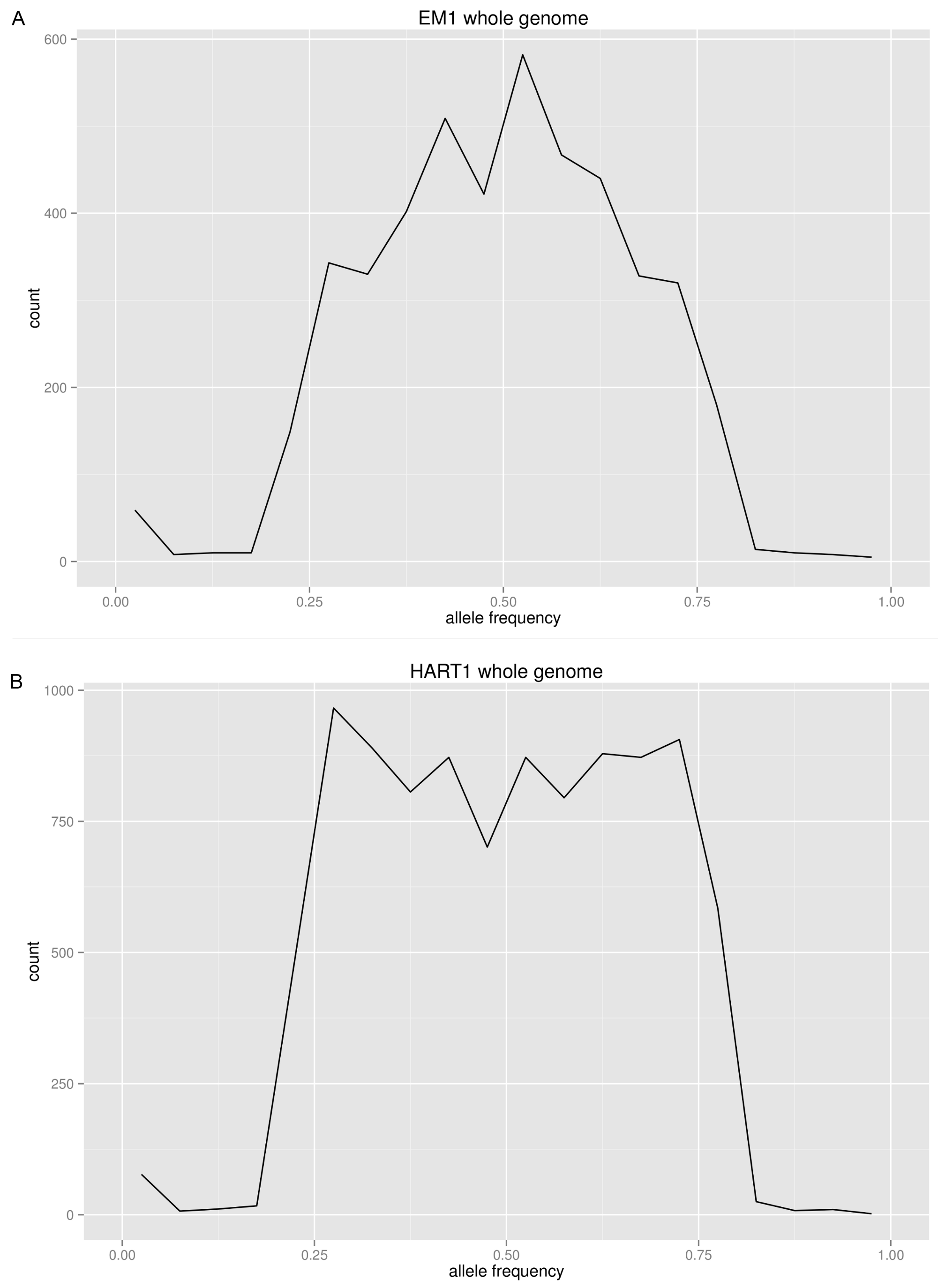

Supplement: Figure S2 — Distribution of allele frequencies of heterozygous single nucleotide polymorphisms (SNPs) across Phytomonas EM1 and HART1 genomes. Y-axis corresponds to allele count; X-axis shows allele frequencies of heterozygous SNPs. A. Phytomonas EM1 whole genome; B. Phytomonas HART1 whole genome. (TIF) [file pgen.1004007.s002.tif]

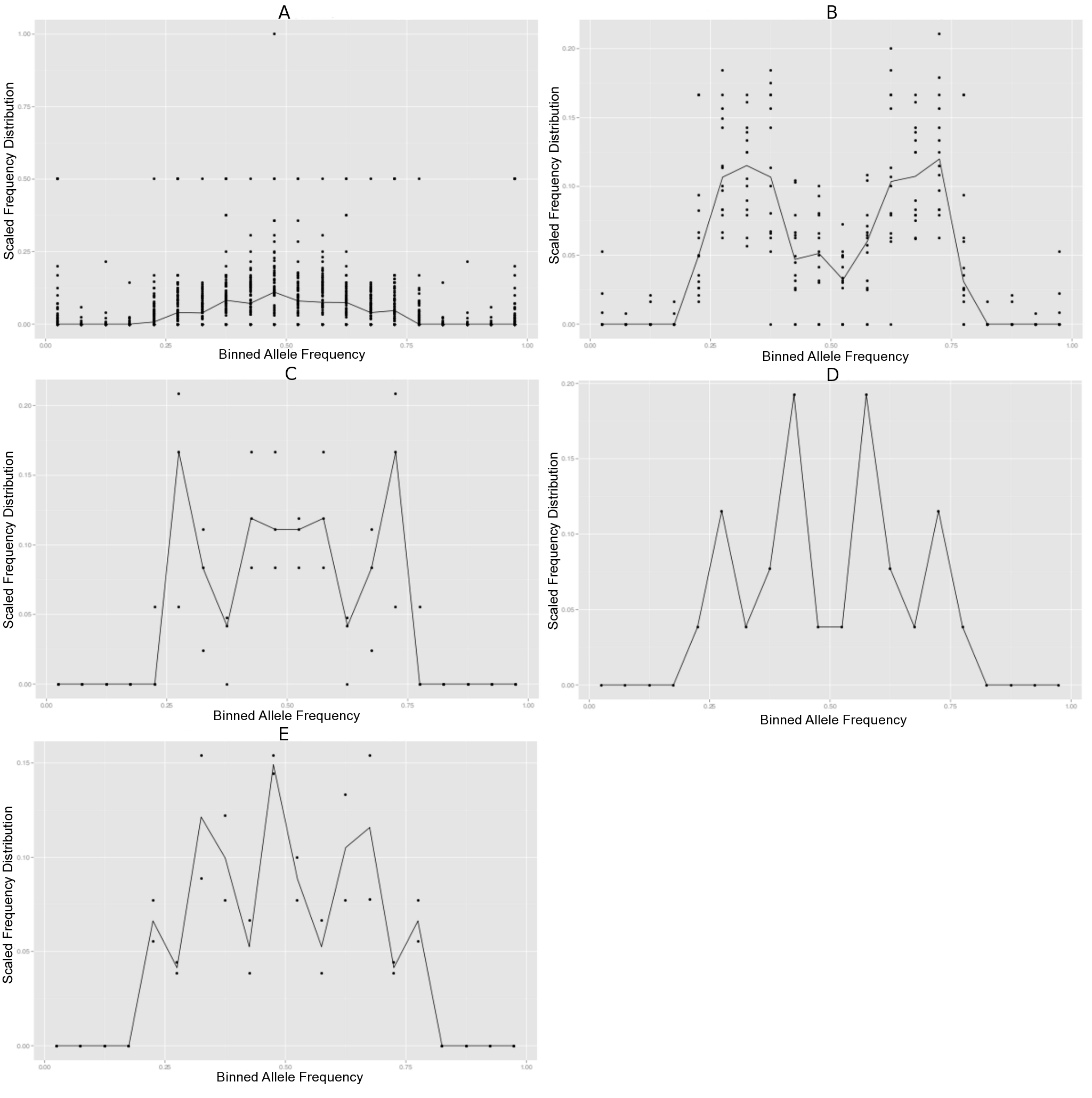

Supplement: Figure S3 — Distribution of allele frequencies according to inferred ploidy for Phytomonas EM1 scaffolds. A, EM1 chromosomes with 2 copies (74 scaffolds and 3,608 SNPs); B, EM1 chromosomes with 3 copies (14 scaffolds and 816 SNPs); C, EM1 chromosomes with 4 copies (3 scaffolds and 84 SNPs); D, EM1 chromosomes with 5 copies (1 scaffold and 26 SNPs) and E, EM1 chromosomes with 6 copies (2 scaffolds and 116 SNPs). (TIF) [file pgen.1004007.s003.tif]

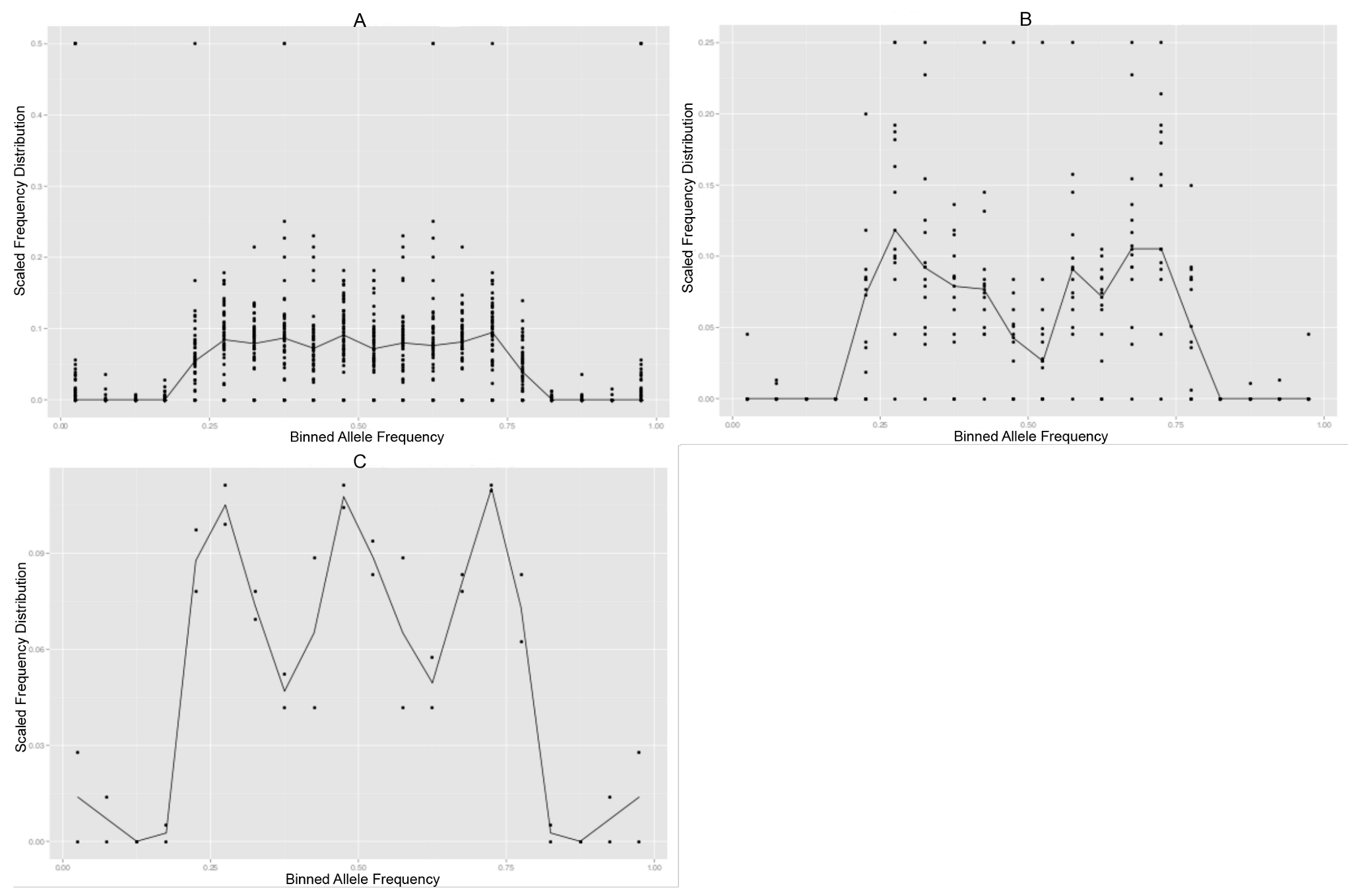

Supplement: Figure S4 — Distribution of allele frequencies according to inferred ploidy for Phytomonas HART1 scaffolds. A, HART1 chromosomes with 2 copies (56 scaffolds and 8,774 SNPs); B, HART1 chromosomes with 3 copies (15 scaffolds and 828 SNPs) and C, HART1 chromosomes with 4 copies (2 scaffolds and 264 SNPs). (TIF) [file pgen.1004007.s004.tif]

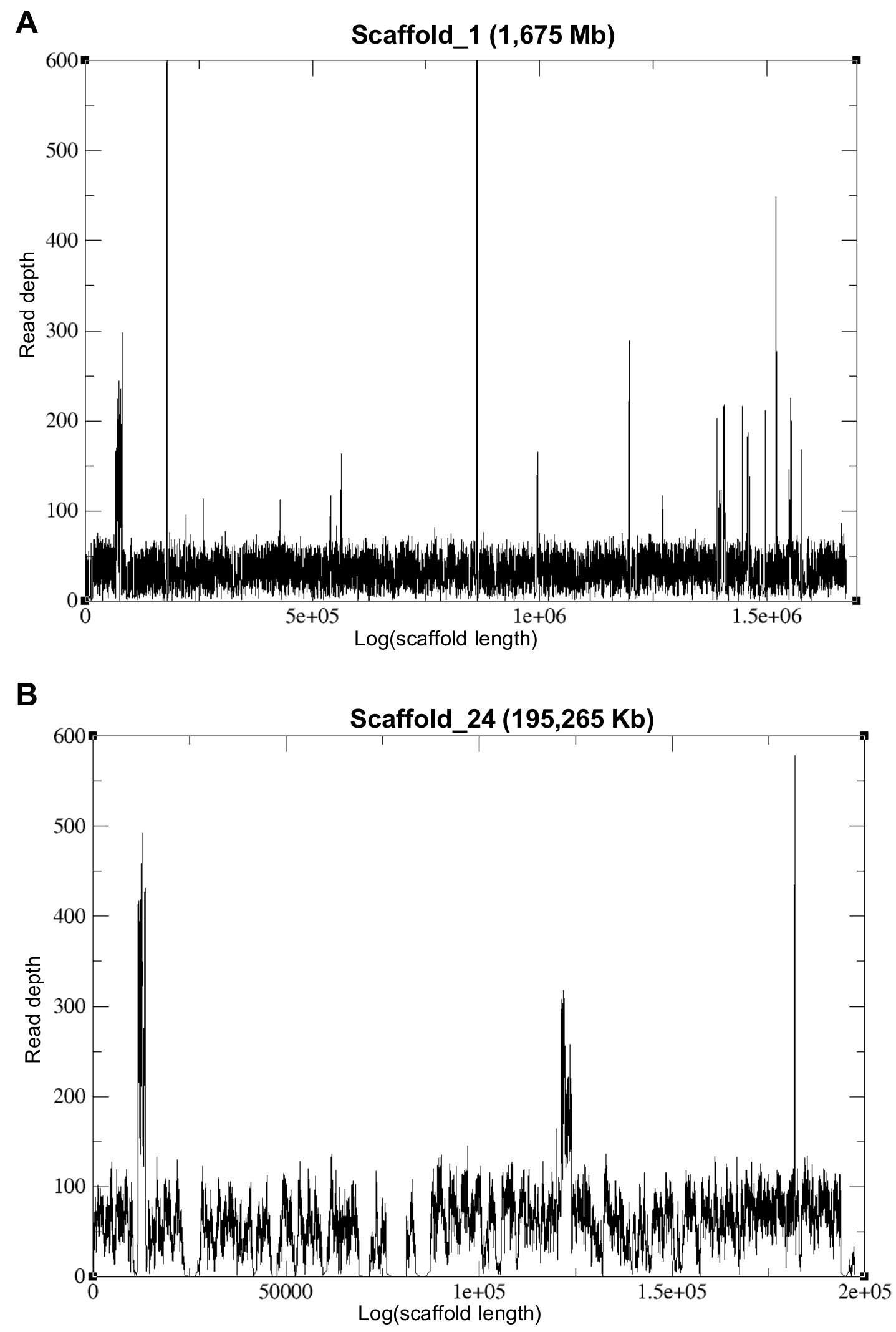

Supplement: Figure S5 — Distribution of read depth along Phytomonas EM1 disomic and tetrasomic scaffolds. Y-axis corresponds to read depth; X-axis shows scaffold length plotted on a log scale. A. Phytomonas EM1 Scaffold_1 (disomic); B. Phytomonas EM1 Scaffold_24 (tetrasomic). (TIF) [file pgen.1004007.s005.tif]

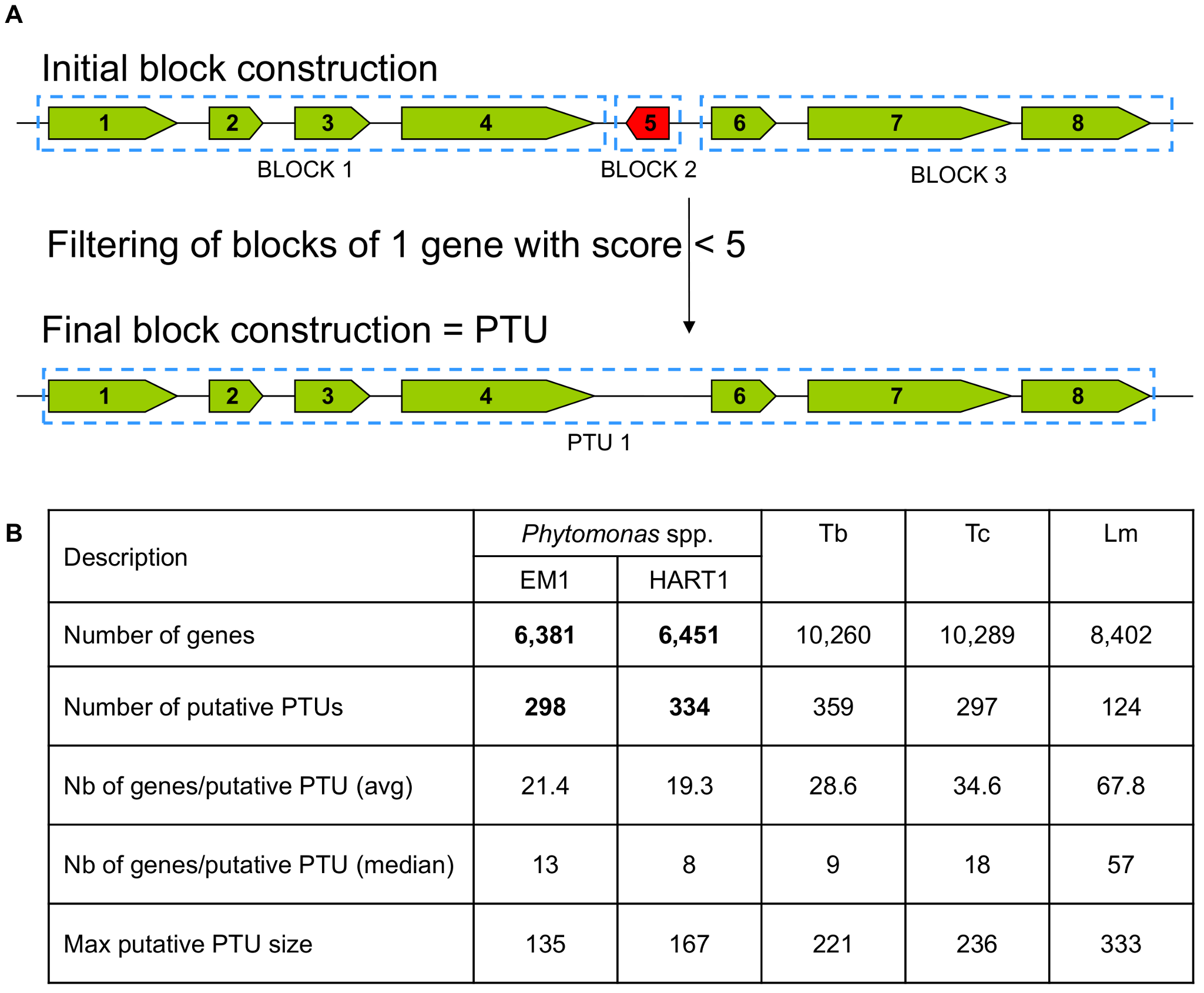

Supplement: Figure S6 — Identification of polycistronic gene clusters (PTUs) in Phytomonas. A. Strategy used for PTUs detection in Phytomonas EM1 and HART1 genomes (details in Text S1). B. Statistics on Phytomonas PTUs. (TIF) [file pgen.1004007.s006.tif]

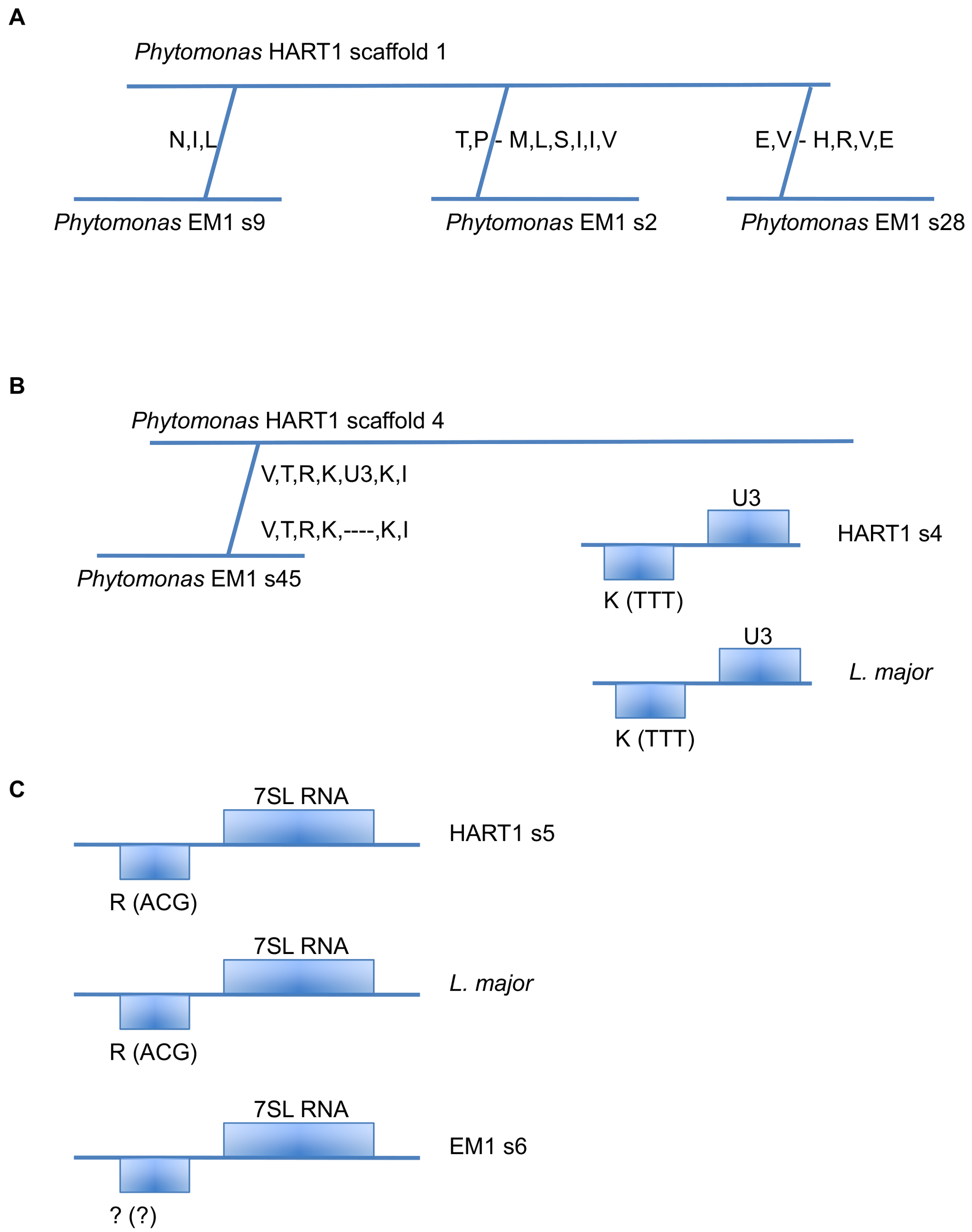

Supplement: Figure S7 — Conservation of tRNA synteny within kinetoplastid genomes. A. Conserved clusters of tRNAs found in HART1 (scaffold 1) and the corresponding scaffolds from EM1. ‘-’ represents tRNA genes absent from one scaffold. B. Partial synteny of tRNA genes between HART1 (scaffold 4) and EM1 (scaffold 45). C. Synteny of tRNA genes associated transcriptionally with other small-RNA genes, U3 and 7SL, in Leishmania major. ‘?’ represents a hypothetical RNA pol III promoter for the downstream 7SL RNA gene. The figure is not drawn to scale. (TIF) [file pgen.1004007.s007.tif]

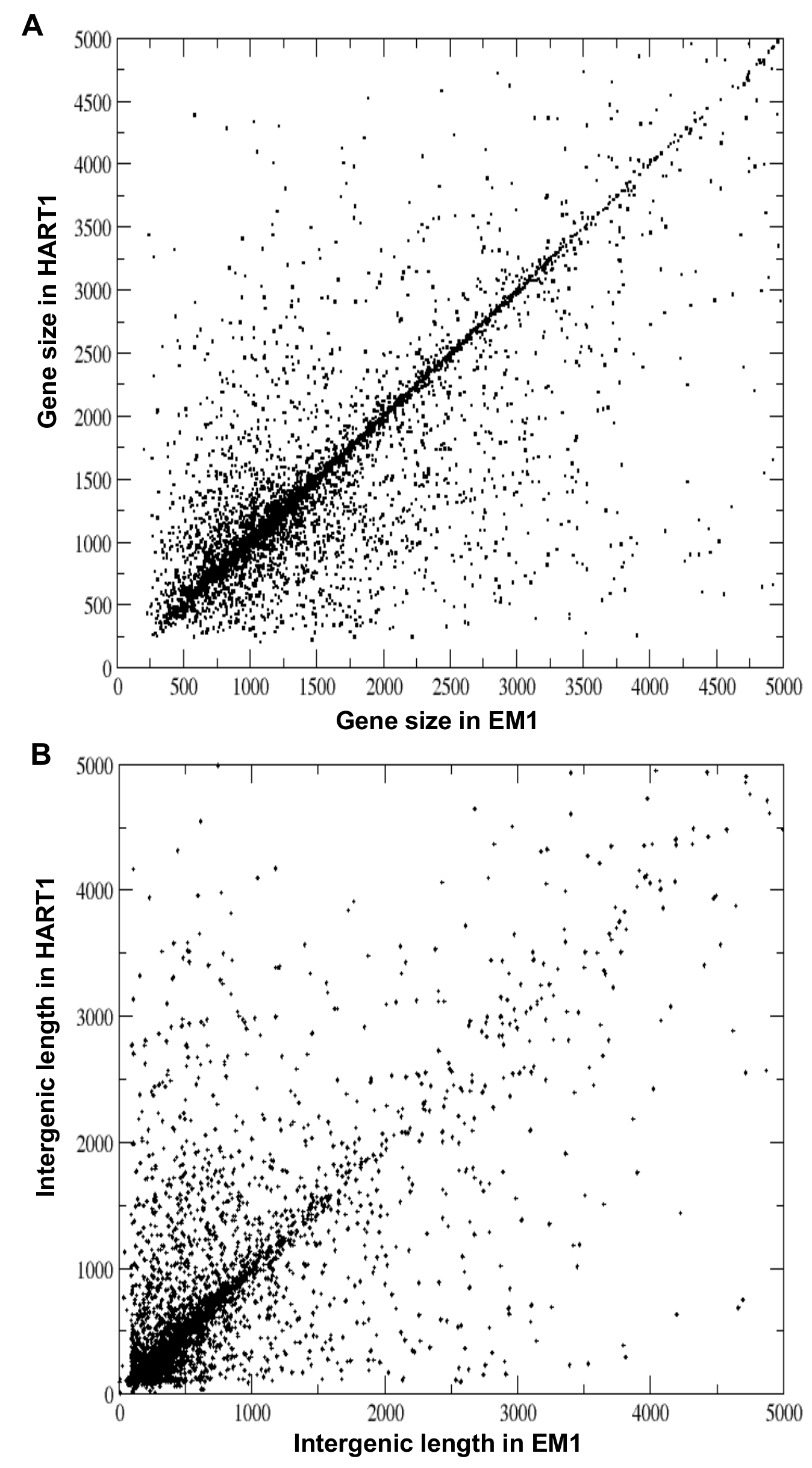

Supplement: Figure S9 — Gene size and intergenic length in Phytomonas EM1 and HART1. Correlation of gene size (A; from 5,006 pairs of BRH) and intergenic length (B; from 3,504 orthologous intergenic regions intra operons - pairs of adjacent orthologous genes - between Phytomonas EM1 and HART1. (TIF) [file pgen.1004007.s009.tif]

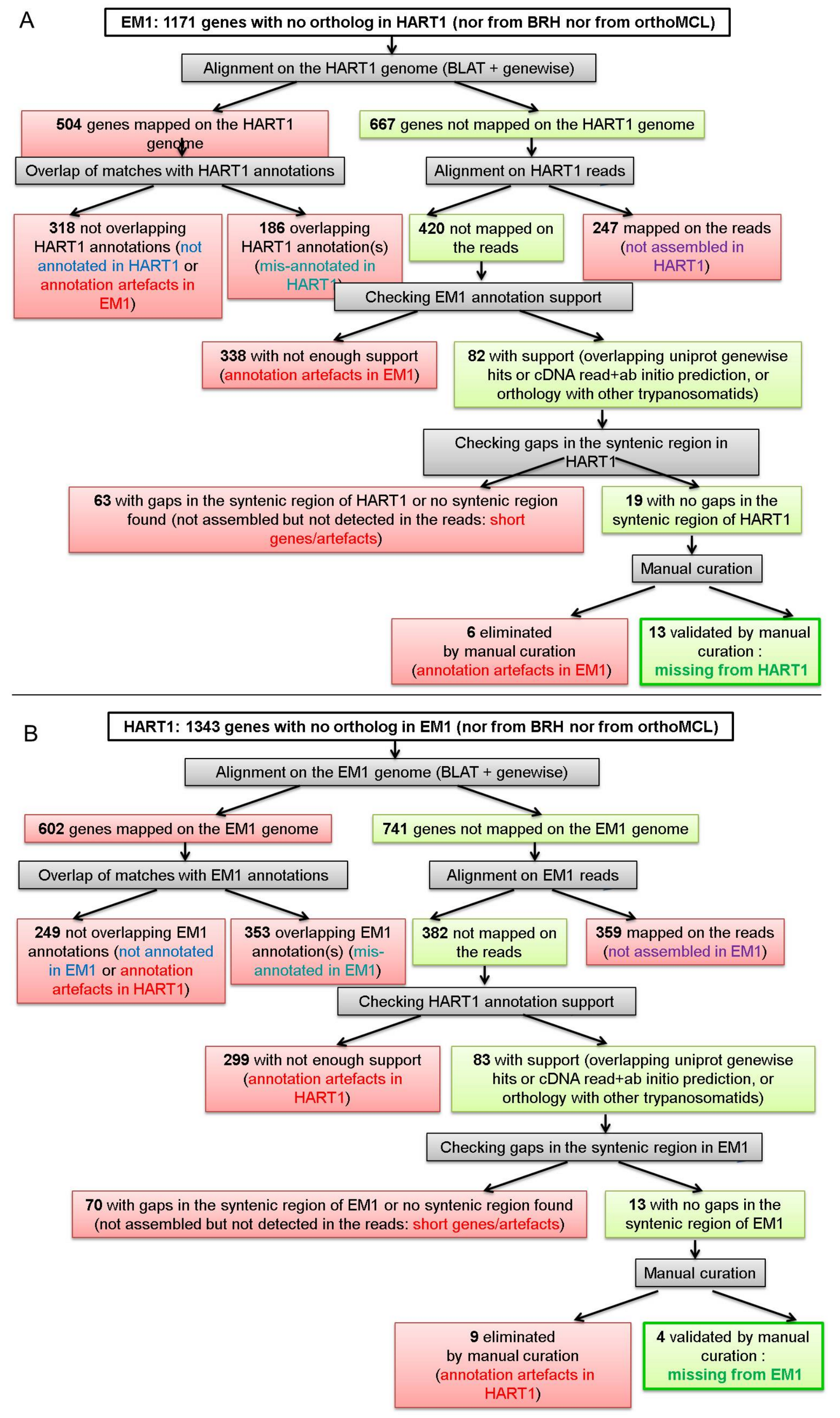

Supplement: Figure S10 — Flowchart of the strategy followed to purify the list of Phytomonas genes with no ortholog in the other isolate. A: EM1 genes with no ortholog in HART1; B: HART1 genes with no ortholog in EM1. (TIF) [file pgen.1004007.s010.tif]

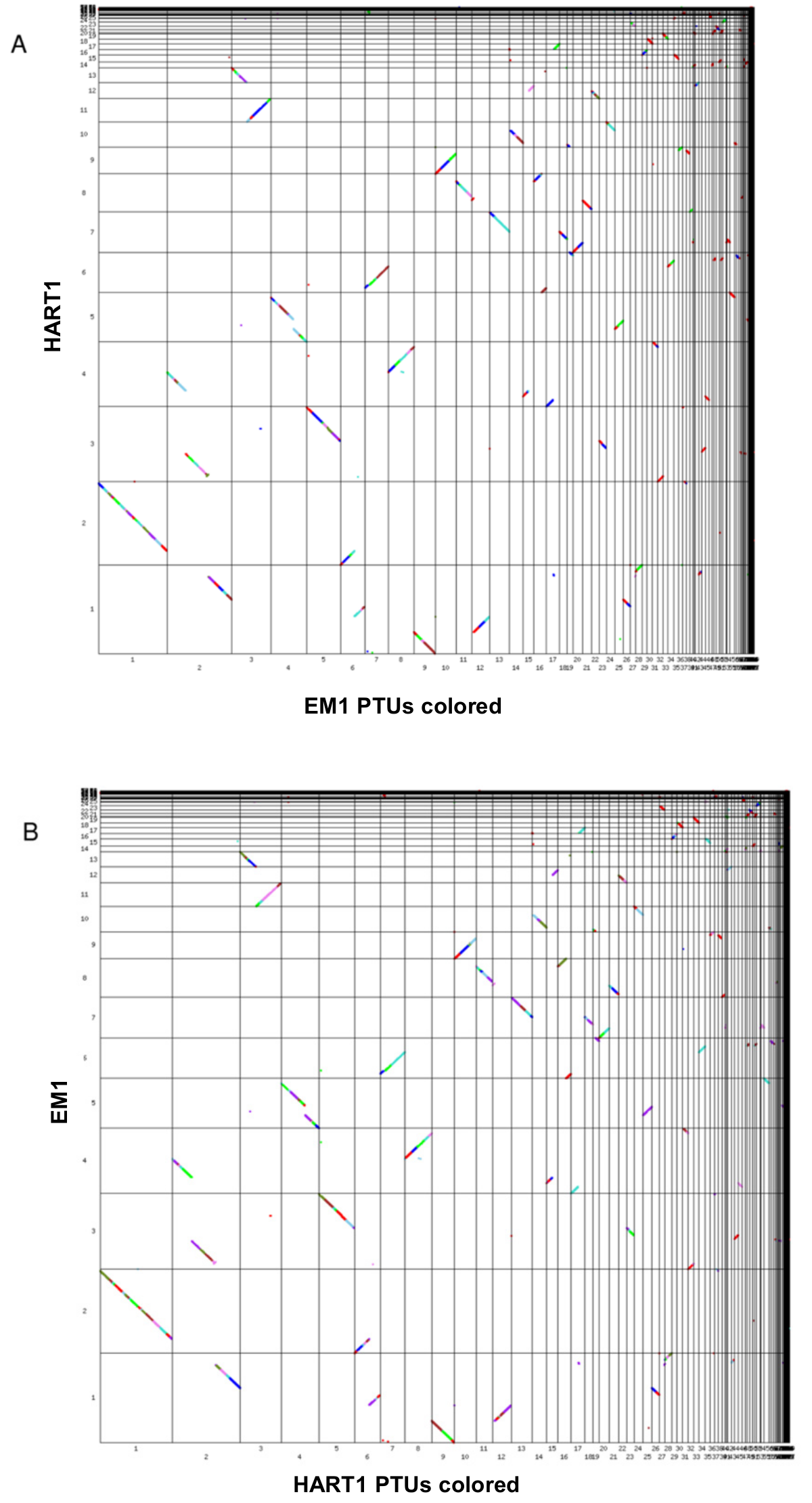

Supplement: Figure S11 — Synteny between Phytomonas EM1 and HART1. Dot plot representation, with PTUs colored. A. EM1 PTUs colored, B. HART1 PTUs colored. Different colors in the diagonal lines mean that the synteny blocks contain several PTUs. (TIF) [file pgen.1004007.s011.tif]

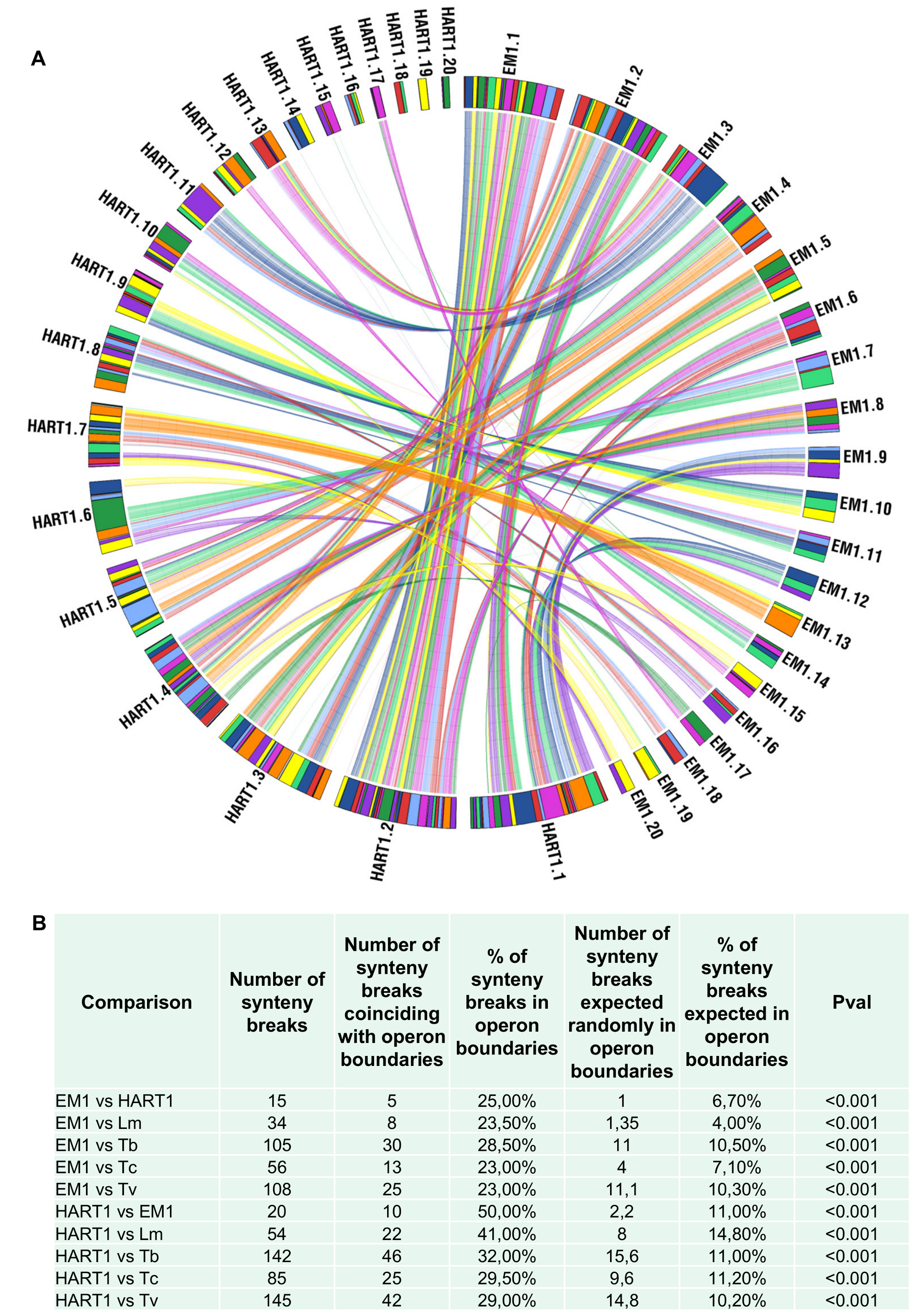

Supplement: Figure S12 — Relationships between Phytomonas EM1 and HART1 PTU's genes. A. Orthologous relationships between Phytomonas EM1 and HART1 genes. PTUs are represented by different arbitrary colors so that PTU boundaries can be visualized. Relationships between orthologous genes are painted with the color of EM1 PTUs in order to facilitate the visualization of operon boundaries conservation B. Synteny breaks between Phytomonas EM1 and HART1 and human trypanosomes. Pairwaise comparison between EM1 and HART1 isolates and kinetoplasitds T. brucei (Tb), T. cruzi (Tc), T. vivax (Tv) and L. major (Lm). P-val: P-value (probability of an observed result arising by chance). (TIF) [file pgen.1004007.s012.tif]

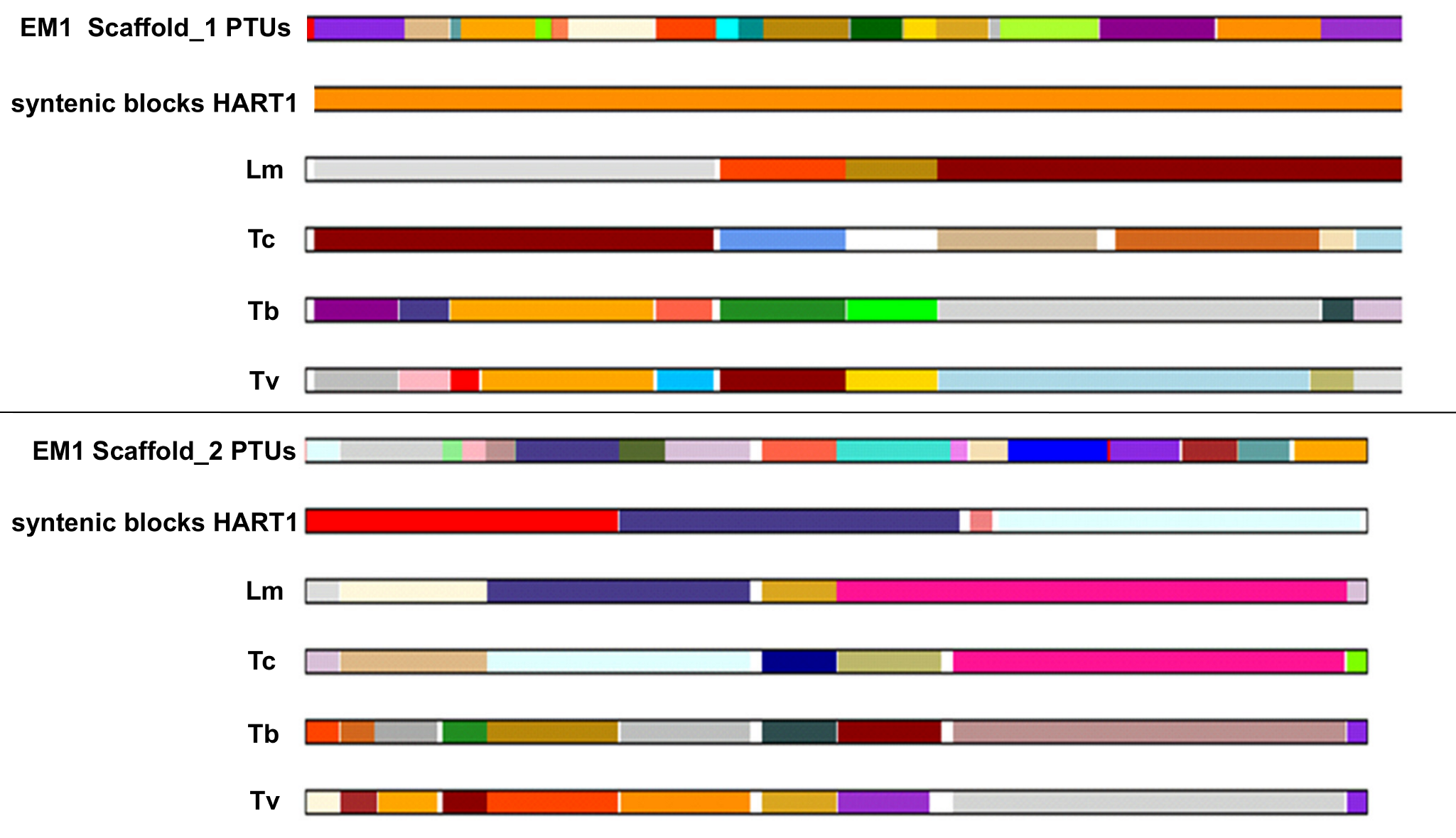

Supplement: Figure S13 — Comparison of PTUs and synteny blocks: example of Phytomonas EM1 scaffolds 1 and 2. For each scaffold, the first line shows the PTUs in different arbitrary colors (changes in colors correspond to PTU boundaries) and the next 5 lines represent the syntenic blocks with 5 other species (each syntenic block is represented by a different arbitrary color: changes in colors correspond to synteny breaks): Phytomonas HART1 (HART1), L. major (Lm), T. brucei (Tb), T. cruzi (Tc), T. vivax (Tv). (TIF) [file pgen.1004007.s013.tif]

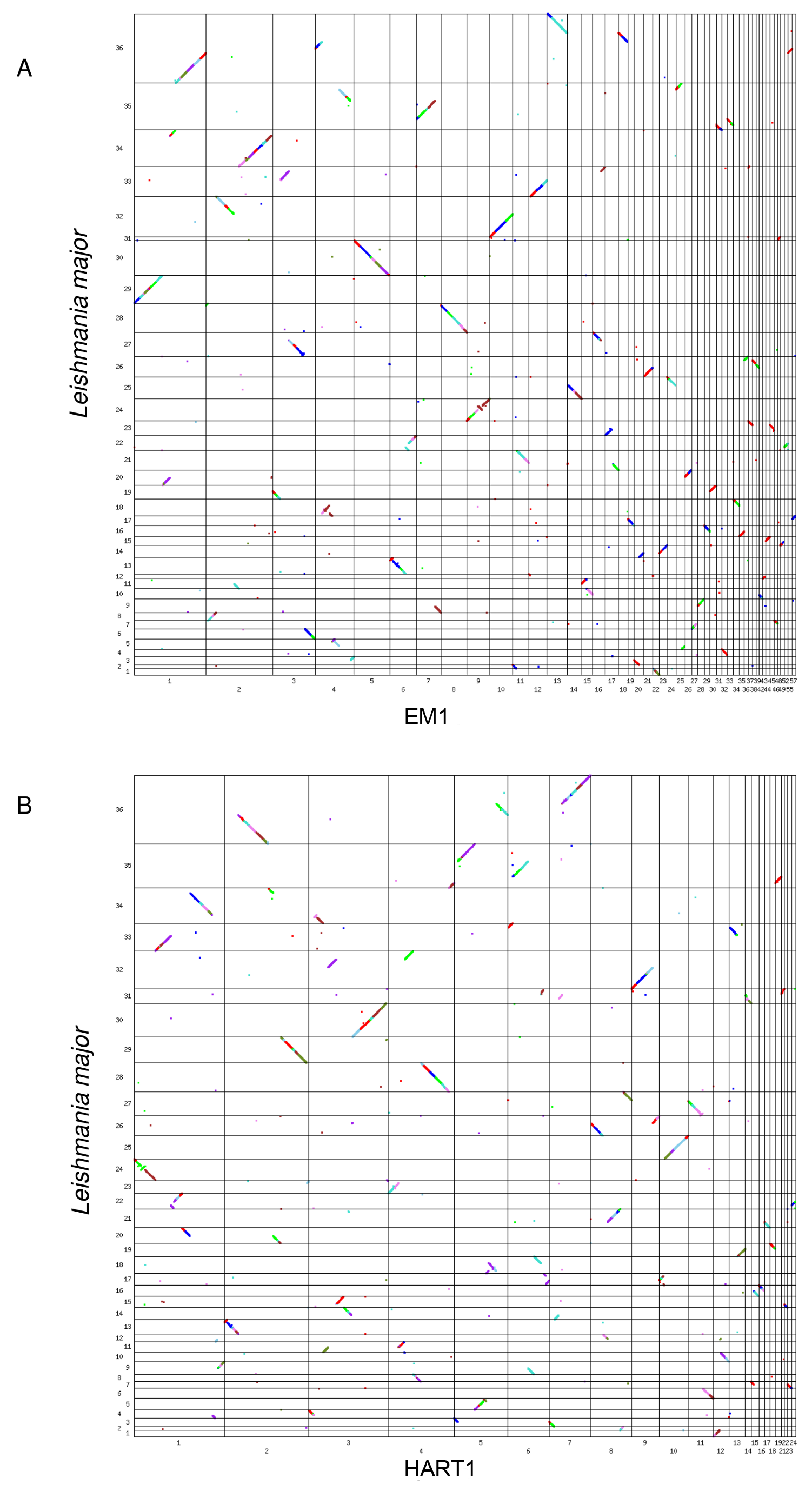

Supplement: Figure S14 — Synteny between Phytomonas EM1/HART1 and Leishmania major (Lm). Dot plot representation of BRH between EM1 and Lm (A; 4,607 genes), and HART1 and Lm (B; 4,322 genes). Each dot represents a pair of genes (BRH), with the position of the EM1/HART1 gene on the EM1/HART1 assembly on the x axis, and the position of the Lm gene on the Lm assembly on the y axis. Genes (dots) are colored according to the EM1/HART1 PTU they belong to. (TIF) [file pgen.1004007.s014.tif]

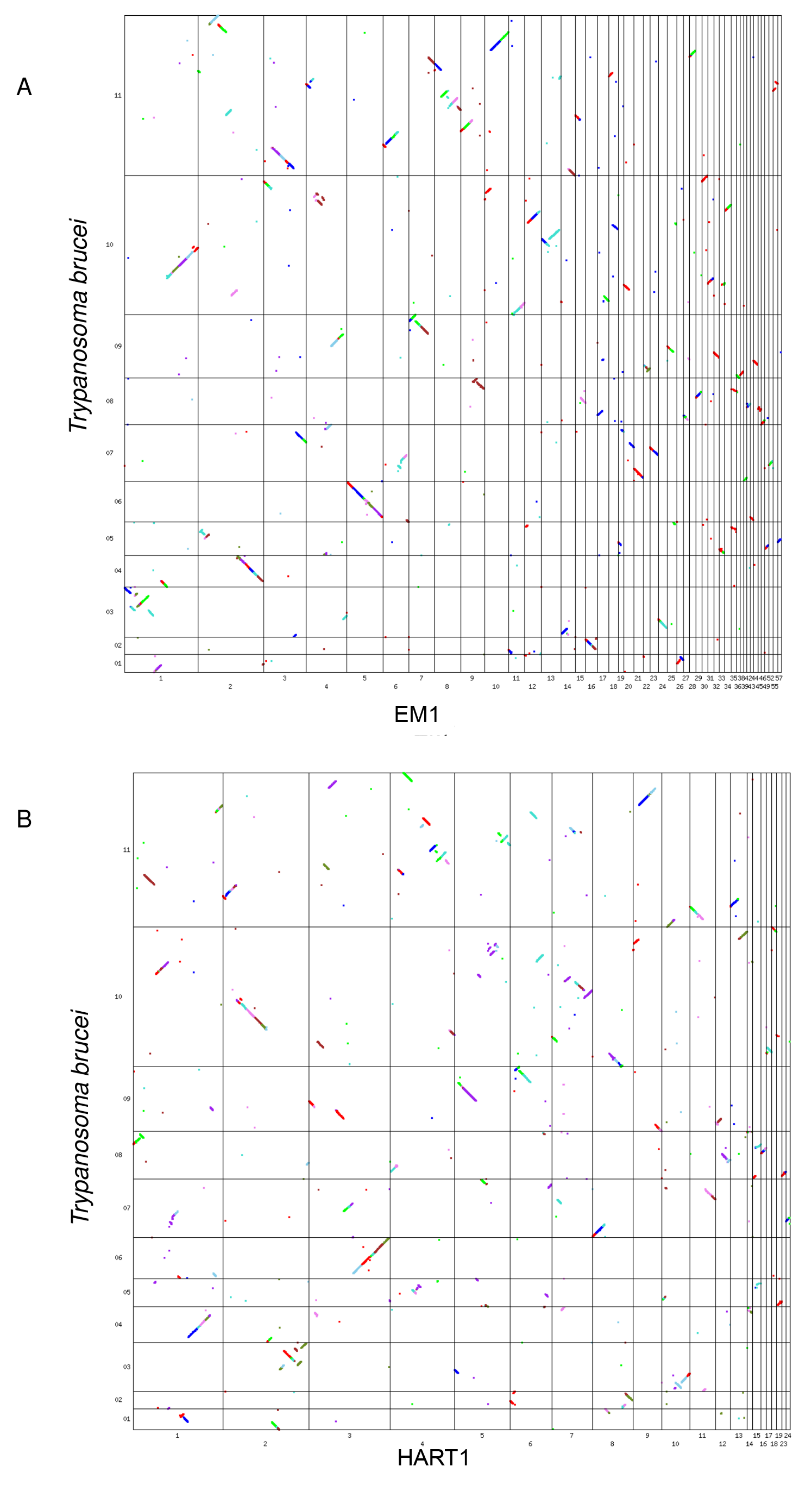

Supplement: Figure S15 — Synteny between Phytomonas EM1/HART1 and Trypanosoma brucei (Tb). Dot plot representation of BRH between EM1 and Tb (A; 4,014 genes) and HART1 and Tb (B; 3,806 genes). Each dot represents a pair of genes (BRH), with on the x axis the position of the EM1/HART1 gene on the EM1/HART1 assembly, and on the y axis the position of the Tb gene on the Tb assembly. Genes (dots) are colored according to the EM1/HART1 PTU they belong to. (TIF) [file pgen.1004007.s015.tif]

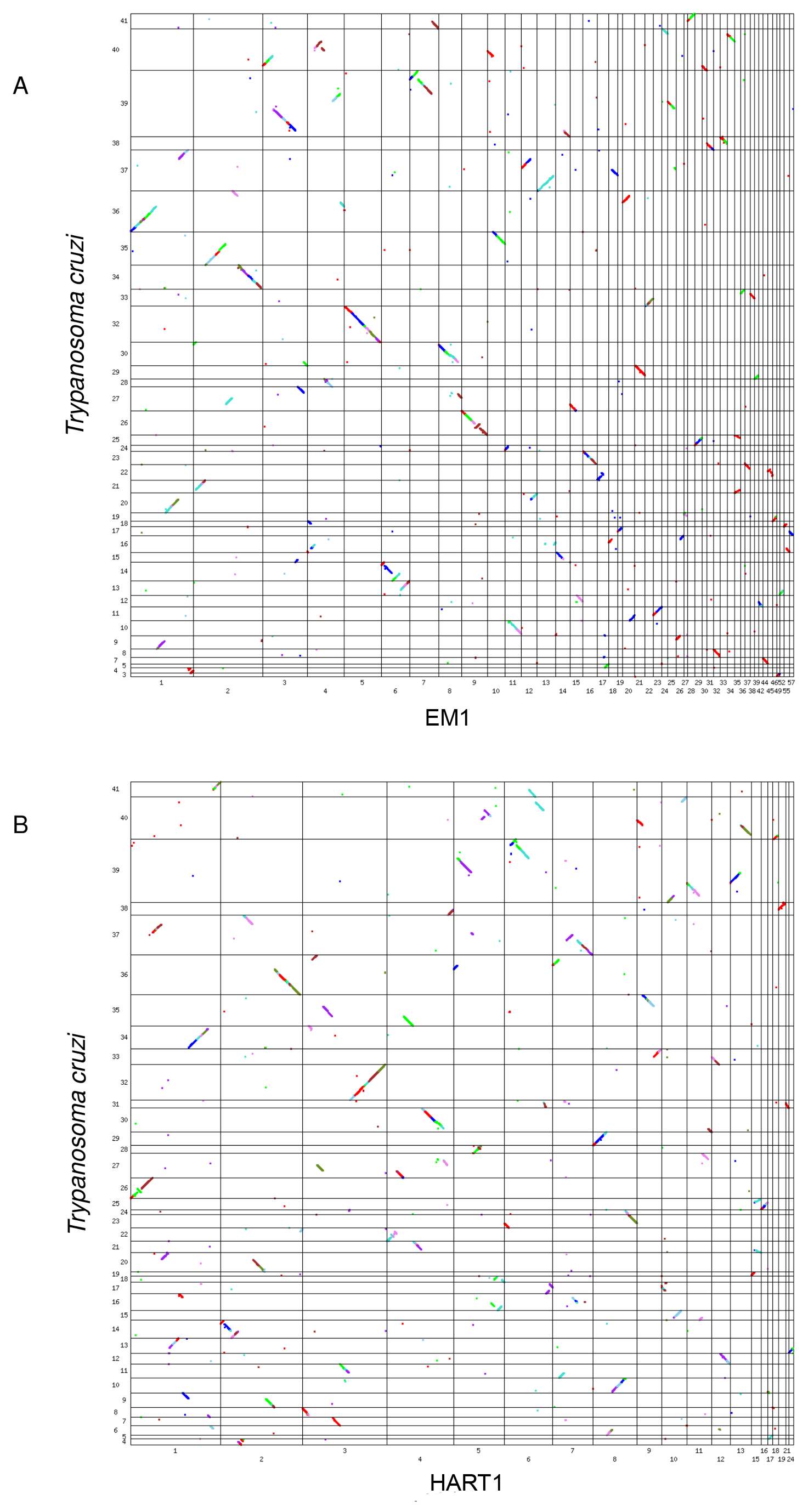

Supplement: Figure S16 — Synteny between Phytomonas EM1/HART1 and Trypanosoma cruzi (Tc). Dot plot representation of BRH between EM1 and Tc (A; 3,646 genes) and HART1 and Tc (B; 3,438 genes). Each dot represents a pair of genes (BRH), with on the x axis the position of the EM1/HART1 gene on the EM1/HART1 assembly, and on the y axis the position of the Tc gene on the Tc assembly. Genes (dots) are colored according to the EM1/HART1 PTU they belong to. (TIF) [file pgen.1004007.s016.tif]

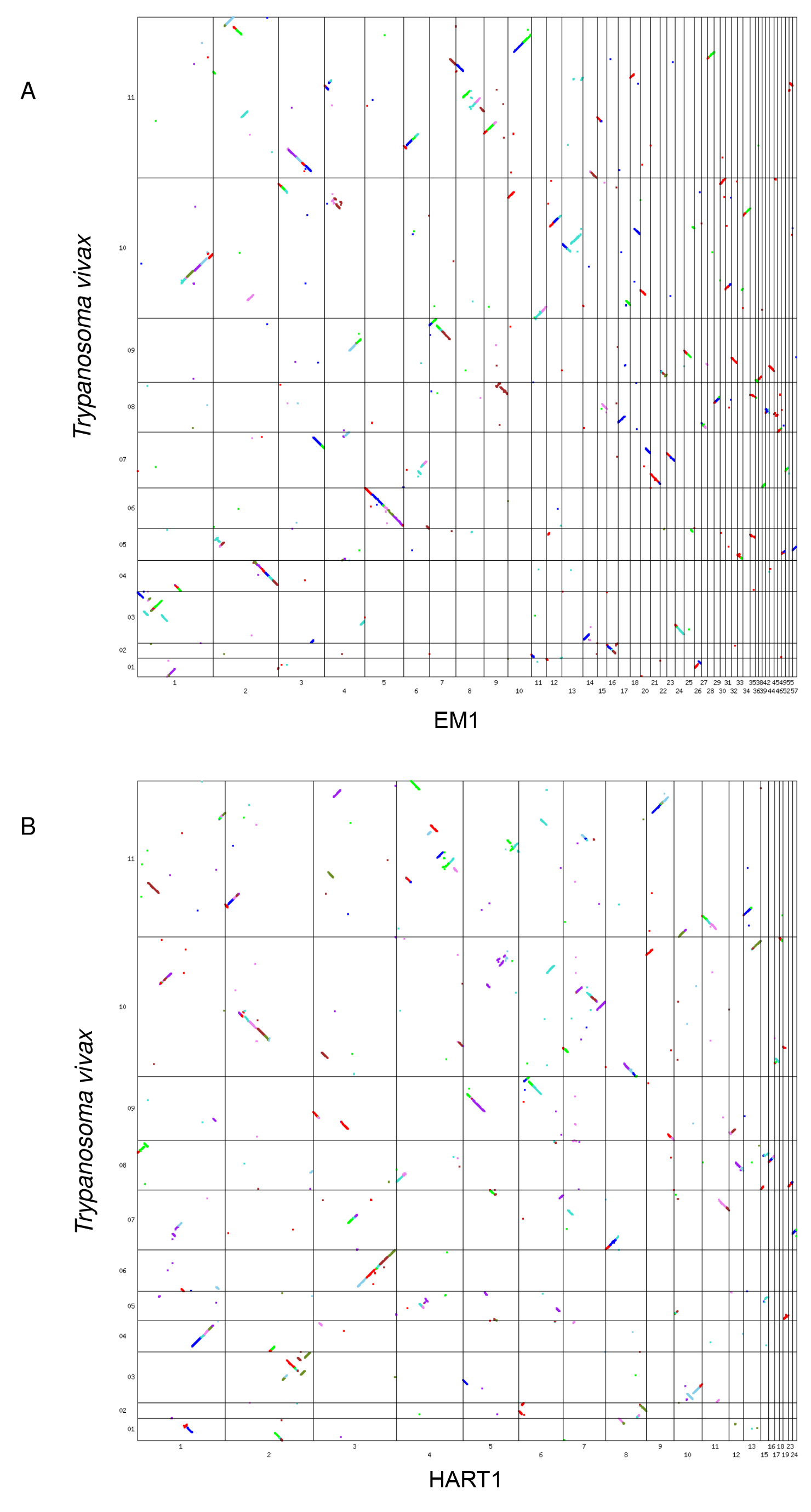

Supplement: Figure S17 — Synteny between Phytomonas EM1/HART1 and Trypanosoma vivax (Tv). Dot plot representation of BRH between EM1 and Tv (A; 3,822 genes) and HART1 and Tv (B; 3,631 genes). Each dot represents a pair of genes (BRH), with on the x axis the position of the EM1/HART1 gene on the EM1/HART1 assembly, and on the y axis the position of the Tv gene on the Tv assembly. Genes (dots) are colored according to the EM1/HART1 PTU they belong to. (TIF) [file pgen.1004007.s017.tif]

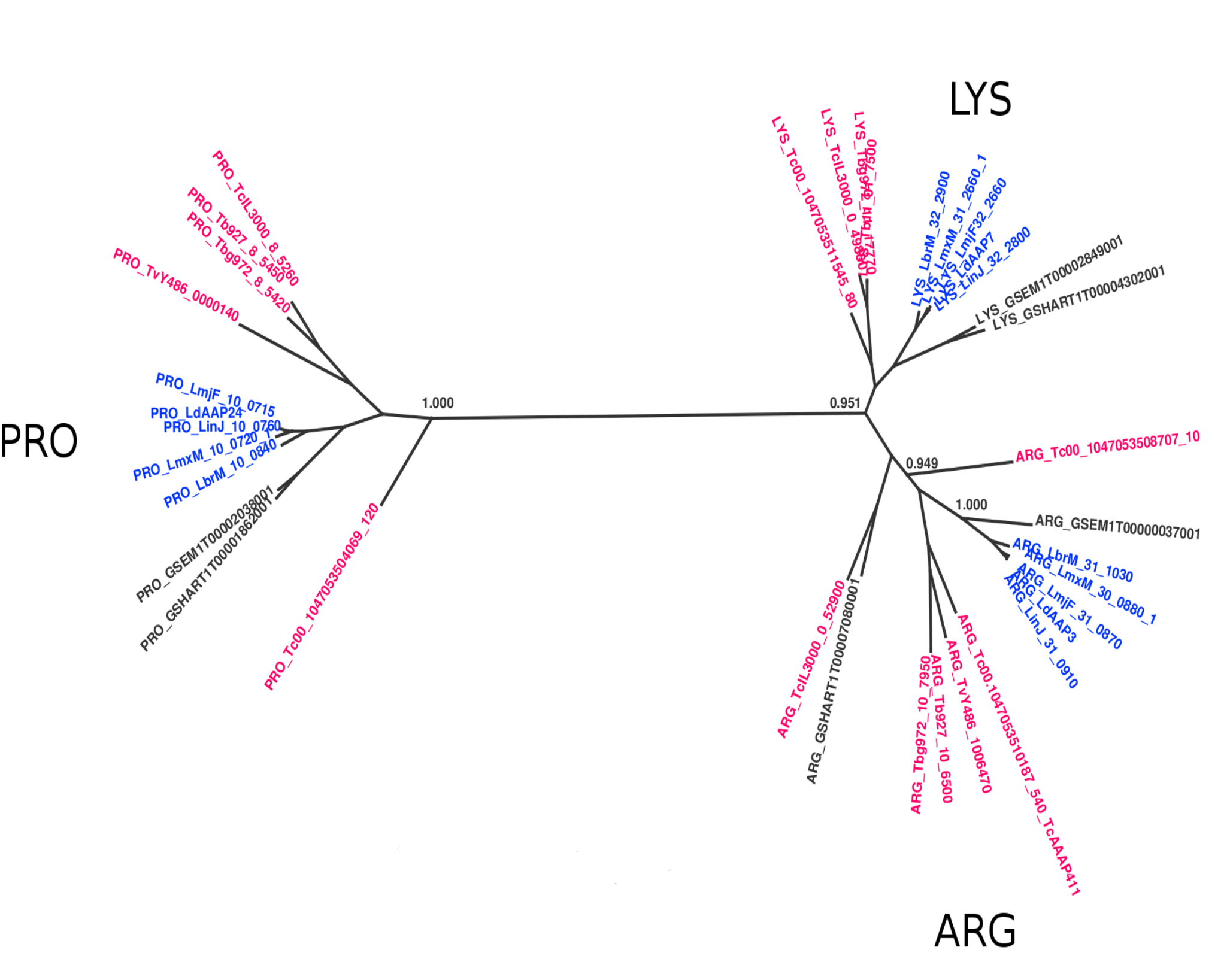

Supplement: Figure S18 — Phylogenetic analysis of global lysine transporters. Radial phylogenetic tree of amino acid transporter proteins, including AAPs from Phytomonas EM1 and HART1, and mammalian trypanosomatids. Trypanosomatid transporter sequences with the indicated ID numbers were taken from GeneDB (http://www.genedb.org). Colors indicate different genera; Leishmania in blue, Trypanosomes in red and Phytomonas in black. (TIF) [file pgen.1004007.s018.tif]

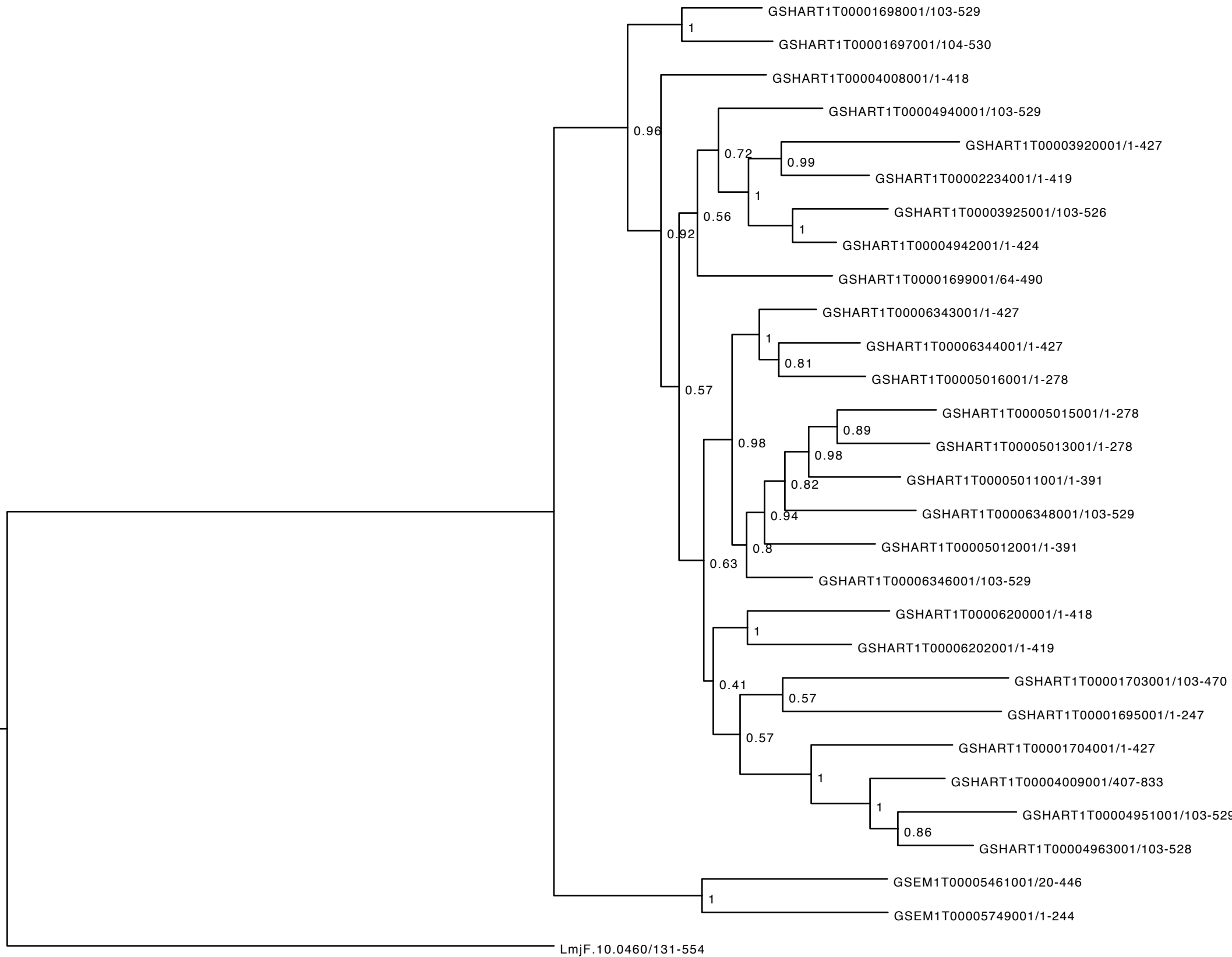

LmjF.10.0460/131-554

0.3

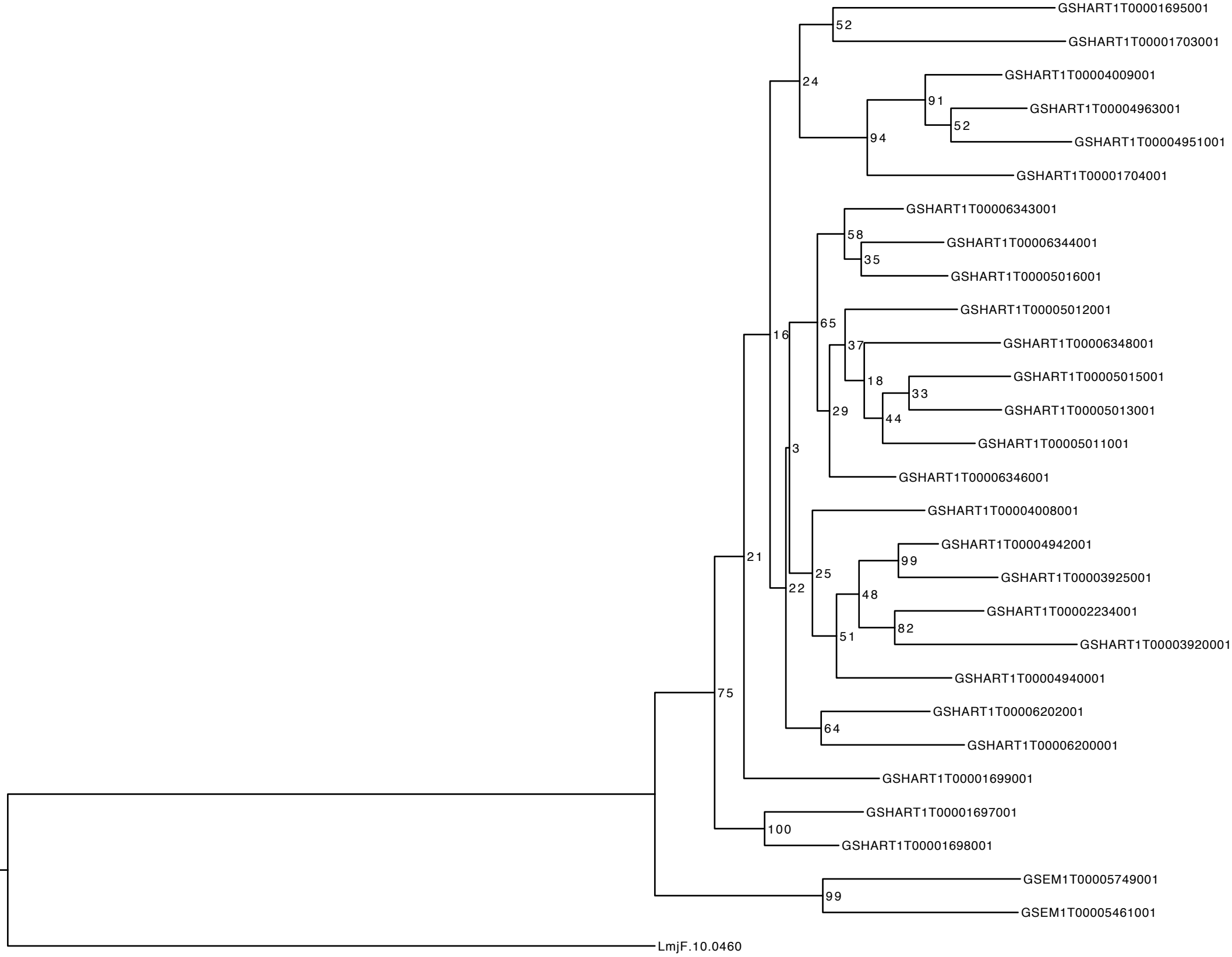

0.3

Supplement: Figure S19 — Phylogenetic reconstruction of gp63 families in Phytomonas. The predicted protein sequences of gp63 orthologs were retrieved from the EM1 and HART1 databases using BLAST, and analysed using MrBayes and PhyML. (PDF) [file pgen.1004007.s019.pdf]

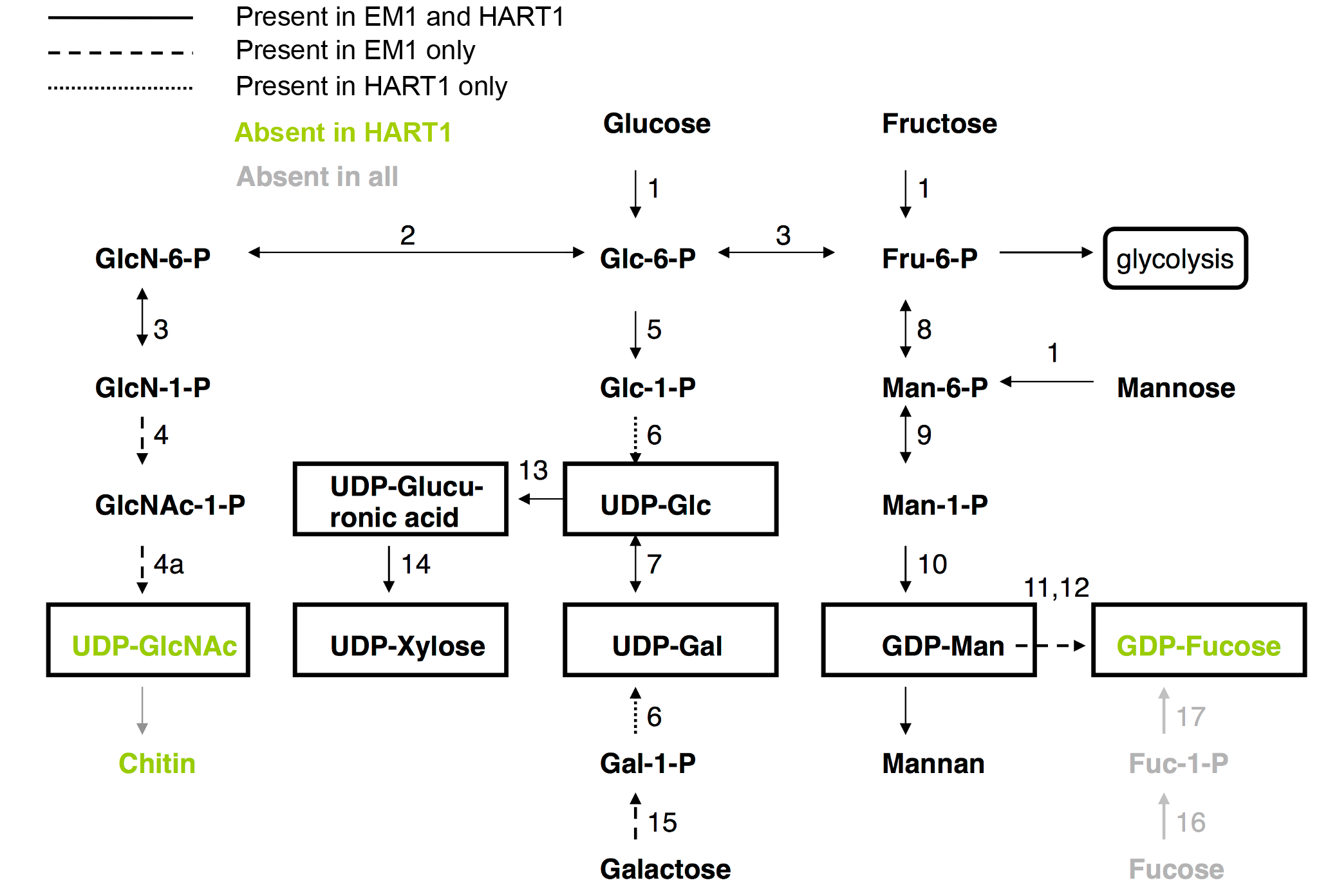

Supplement: Figure S20 — Protein glycosylation in Phytomonas. Steps in the formation of the activated sugar residues for the glycosylation of proteins. Abbreviations: Glc, glucose; GlcN, glucosamine; GlcNAc, N-acetyl glucosamine; UDP, uridylyldiphosphate; Gal, galactose; Fru, fructose; Man, mannose; GDP, guanidyldiphosphate; Glr, glucuronic acid. Enzymes: 1, Glucokinase/hexokinase; 2,glucosamine-6-phosphate deaminase; 3, phosphoglucosamine mutase; 4 and 4a, bifunctional enzyme: glucosamine-1-phosphate acetyltransferase/UDP-N-acetylglucosamine pyrophosphorylase; 5, phosphoglucomutase; 6, UDP-galactose/glucose pyrophosphorylase (2.7.7.64); 7, UDP-glucose 4-epimerase; 8, Mannos-6-phosphate isomerase; 9, phosphomannomutase; 10, mannose-1-phosphate guanyltransferase/GDP-D-mannose pyrophosphorylase; 11, GDP-mannose 4,6-dehydratase; 12, GDP-L-fucose synthase; 13, UDP-glucose 6-dehydrogenase; 14, UDP-glucuronic acid decarboxylase; 15, galactokinase; 16, fucose kinase; 17, Fucose-1-phosphate guanylyltransferase. (TIF) [file pgen.1004007.s020.tif]

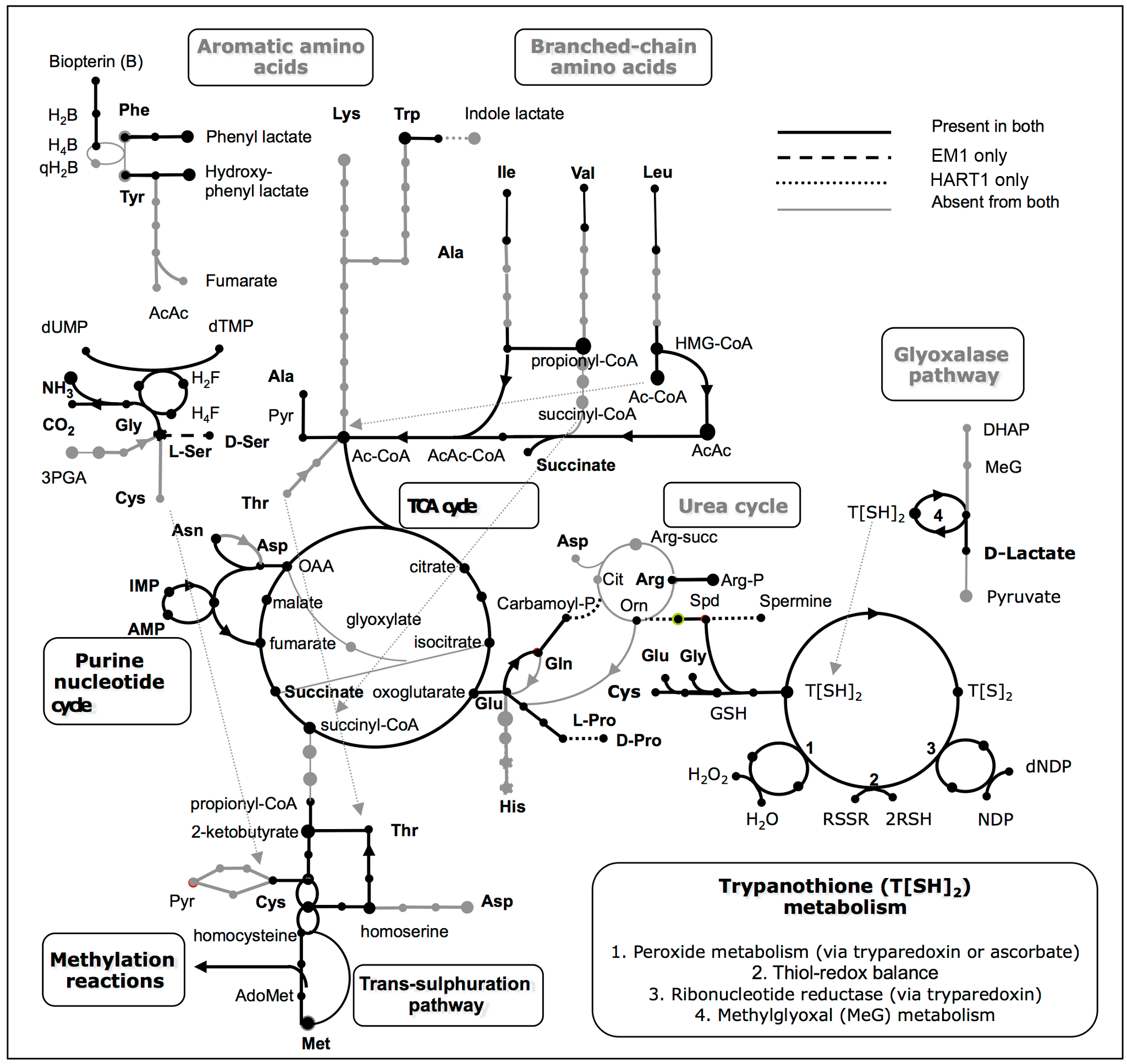

Supplement: Figure S21 — Amino acid and dithiol metabolism in Phytomonas EM1 and HART1 isolates. (TIF) [file pgen.1004007.s021.tif]

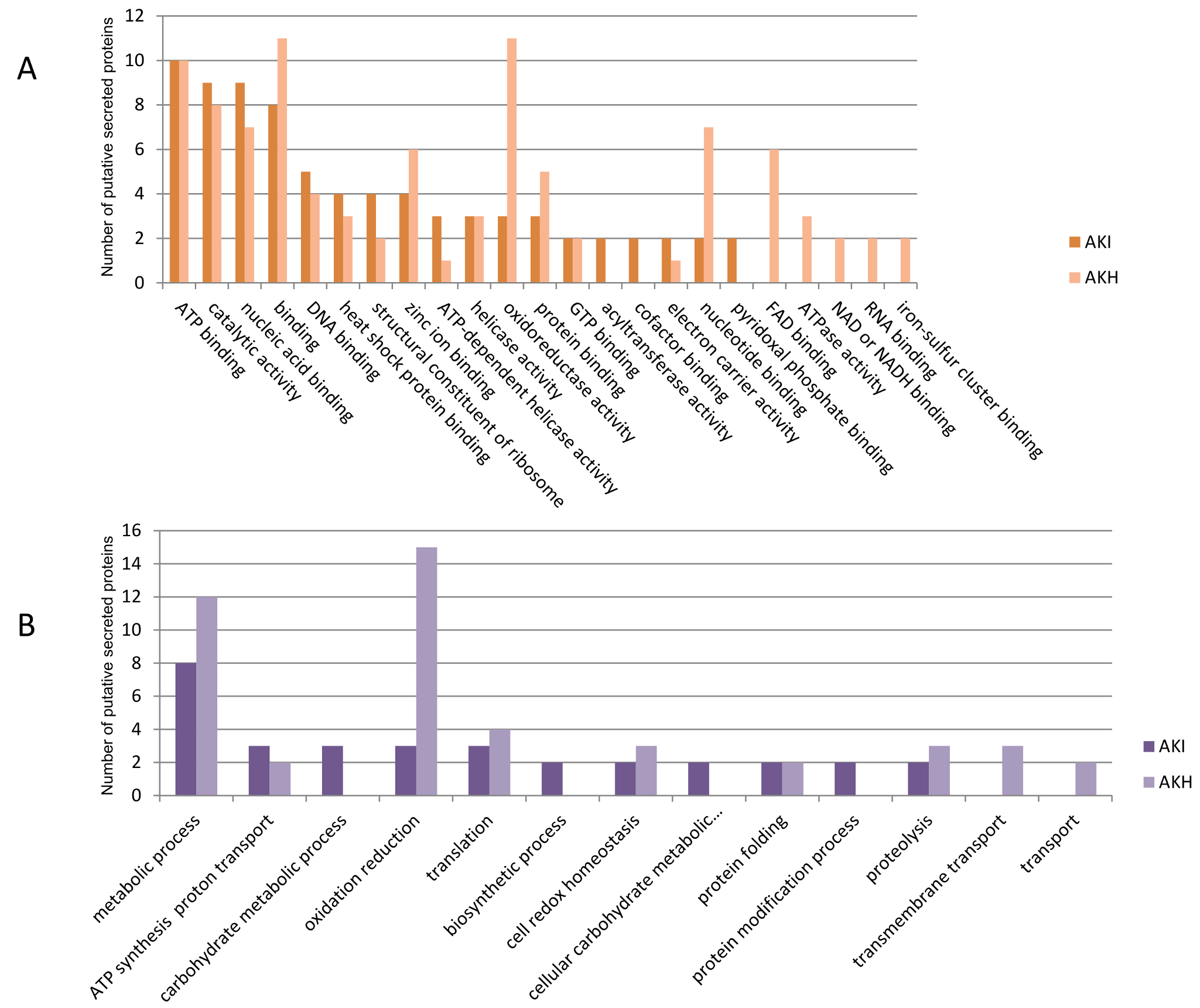

Supplement: Figure S22 — Gene Ontology (GO) Classification of the putative secretome of Phytomonas HART1 and EM1. The y-axis indicates the number of putative secreted protein sequences found under each GO term; x-axis corresponds to the GO classification of the molecular function (panel A) and GO classification of the biological process (panel B). AKI corresponds to Phytomonas HART1, AKH corresponds to Phytomonas EM1. (TIF) [file pgen.1004007.s022.tif]

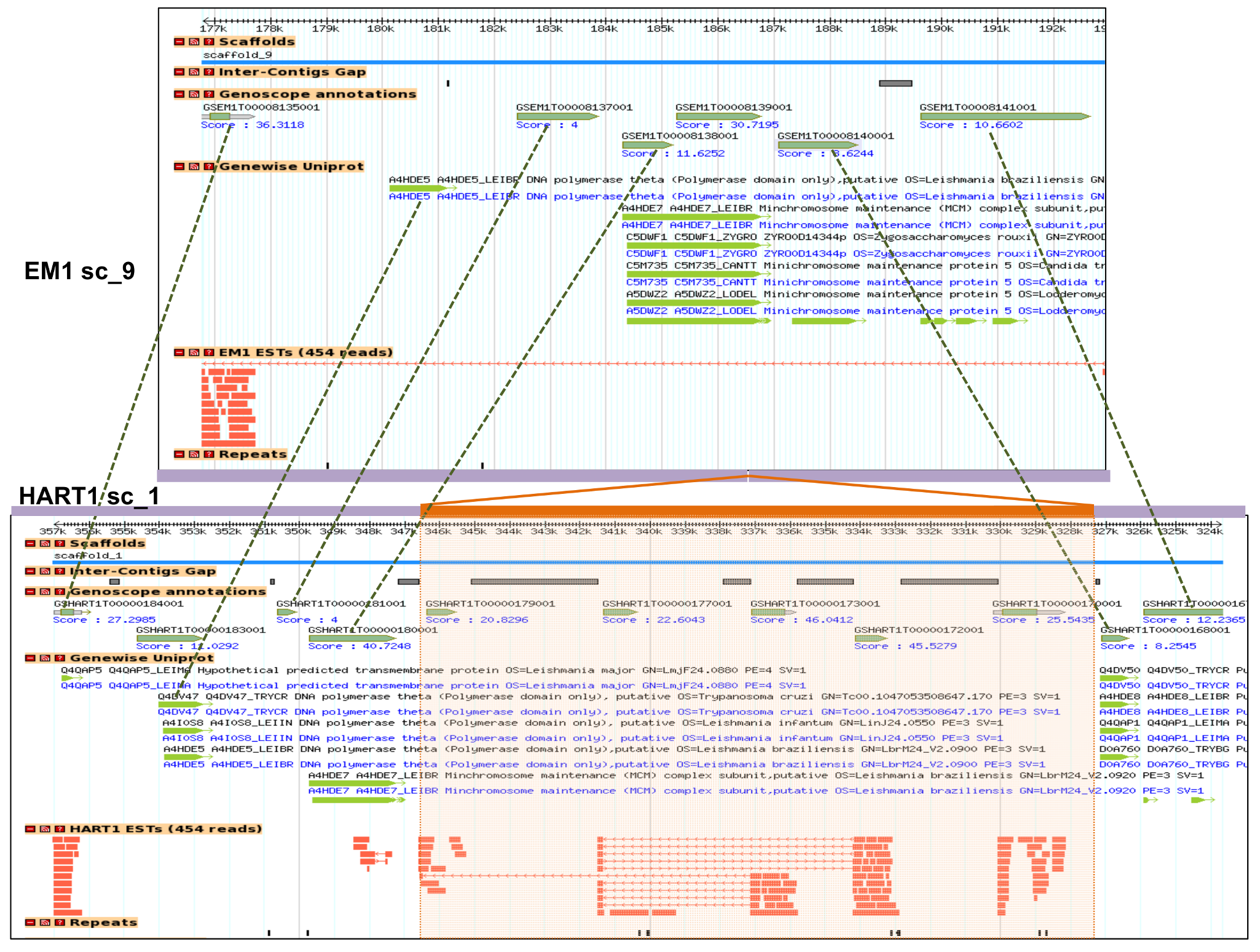

Supplement: Figure S23 — Genome browser view of the aspartic protease cluster. The upper part of the figure is a view of the EM1 genomic region lacking the cluster. The lower part represents the HART1 genomic region of the aspartyl protease cluster. Dotted lines indicate the synteny of the two regions. (TIF) [file pgen.1004007.s023.tif]
