# Supplementary material for: The Streamlined Genome of Phytomonas spp. Relative to Human Pathogenic Kinetoplastids Reveals a Parasite Tailored for Plants
Source: PLoS Genet. 2014 Feb 6;10(2):e1004007. doi: 10.1371/journal.pgen.1004007 (PMC3916237; doi:10.1371/journal.pgen.1004007)
Supplement: Figure S8 — Phytomonas HART1 maxicircle. (PDF) [file pgen.1004007.s008.pdf]

```
                     20        40        60        80
Phytomonas HART1    -----------------------------------------------------------------------------------------------
Translation ORF/CDS
Phytomonas serpens  AAGCTTTTAATAATTCACAGTTAAATAAAAGTAATATATTACAGTCGAACAAAAATAAAATGTTATTGTAGTTATATAACTGTCAAAACTTAAAT
Translation ORF/CDS

                    100       120       140       160       180
Phytomonas HART1    ------------------------------------------------------------------------------------------------
Translation ORF/CDS
Phytomonas serpens  ATTTAAATTTTAAGTATTATATATAGAGATATAAATAACAAAGTAAAGTATAGTATGATATTAATAGTATGAAAACAATCAAAGAAGTGTGTAATA
Translation ORF/CDS

                    200       220       240       260       280
Phytomonas HART1    ------------------------------------------------------------------------------------------------
Translation ORF/CDS
                    5' and 3' ends uncertain
Phytomonas serpens  AAAATAAAAAAGCATAAGAAATAAATCTTTTATAAAATTGTTATTTATTTAAATATTGATGTAGTAAAGTGTAAATAAATATATTAATTTTGTGTC
Translation ORF/CDS

                    300       320       340       360       380
Phytomonas HART1    ------------------------------------------------------------------------------------------------
Translation ORF/CDS
Phytomonas serpens  AAAATATATTTTTTATAATTAACAAGATTGGTATTTAATTCTTAAATATTAAATTTGTTGTTTTATATTTGGATTAATATTTTAAAAATAGAGTAT
Translation ORF/CDS

                    400       420       440       460       4
Phytomonas HART1    ------------------------------------------------------------------------------------------------
Translation ORF/CDS
Phytomonas serpens  TAATATTTAAAATTTAAAATTAAATTGAACTGTTATTATTAGTTTATTATTTTTTATTTTATGTTTAAATATATAATTAATGATGACACAGTTGTT
Translation ORF/CDS

                    480       500       520       540       560
Phytomonas HART1    ------------------------------------------------------------------------------------------------
Translation ORF/CDS
Phytomonas serpens  TTATATGTAACTAATTAAAATAGTAAGATTATGTATTTAATTTAAAAAAAAAATATTTAAAAATAAAGTAAATATTATGTATATTTTAAAAATTAA
Translation ORF/CDS
```

580 600 620 640 660

12S

Phytomonas HART1 ---AAAATATTT
Translation ORF/CDS

Phytomonas serpens AAAAGTTAGCCAATAAAGTATTAAATAAATTAATAATTTTAATTGAGTATTAATATTACTAATGTAATTTGTACTTAATAGTGAGATATAAACAA
Translation ORF/CDS

680 700 720 740 760

Phytomonas HART1 ATGGTATAAAATTTATAATAAAACAAATATTATTCTAATATTTAATATTTGTTAAT-AAAAAGTAAATGTAATTTTATTAAAAAGTATTATTATAA
Translation ORF/CDS

Phytomonas serpens AAATTGGTGTGAAAATAATAGTAATAATAGTATTCTAATATTTAGTATTTGTTAATTTAAAAGTAAAAATATCATAA-TAAAAAGCATTATTATAT
Translation ORF/CDS

780 800 820 840 860

Phytomonas HART1 CATAAA-ATTTATAAAAATACAATAATATTGTTAATAAAATATTAAAATTTCAAAAACGTTTATTAAAGGCGTTAGTCTAAGAACTTTATTTAAGA
Translation ORF/CDS

Phytomonas serpens AATATATATTTTTAAAATTACAAAAATATTGTTAATAAAGTCTTCAAATTTTAAAAACGTTTATTAAATGCGTCTATCTAAGAACAATATTTAAGA
Translation ORF/CDS

880 900 920 940 9

Phytomonas HART1 TCATTCTAGTAAATATTTTTATATTTTTTTAATTCTATTTAATATGTTAATTACCCTATAATGTTATATATAGTAGCATAGTAATTTGTTAATTAA
Translation ORF/CDS

Phytomonas serpens TTATTCTGGTAAATATTTTTAATTTTTAATAGTTCTAATAAATATATAAAAAACCCTTGTGTGTAATAAAATGTAGCATAGTAATTTGTTAACTAA
Translation ORF/CDS

)60 980 1,000 1,020 1,040

Phytomonas HART1 TTATTAAAGTGTTCCATAGAAAATTTTAAAATTAAAGCAATACGTGTAATATATAAATTAAAATAAAGATTTTAAAAAAAGTCAAATCATTAAAAT
Translation ORF/CDS

Phytomonas serpens TTATTAAAGTGTTCCATAGAAAATTTTAAAATTAAAACAATCATAGTAATTTATGAATTAAAATAAAAATTTTAAAAAAAATTAAAACATTAAAAT
Translation ORF/CDS

1,060 1,080 1,100 1,120 1,140

Phytomonas HART1 AGGGCAAGTCCTACTCTCCTTTACAAAGAGACCATATAATATGTATTTGTATGTTTGATTGGGGCAATACTATATTTAAATATATAGAAAAGAAC
Translation ORF/CDS

Phytomonas serpens AGGGCAAGTCCTACTCTCCTTTACAAAGAAACCATGTAATATGTATTTGTATGTTTGATTGGGGCAATACTATATCTTTATATATAGAATAAGAAC
Translation ORF/CDS

1,160 1,180 1,200 1,220 1,240

Phytomonas HART1 TATAATTATTGAAATAATAAAAGGTTCGAGCAGGTTAACAAGCATCAAAAAAAATGTGTTTCATCGTCTACTTATTATTATAATTGATTGTTCATC
Translation ORF/CDS

Phytomonas serpens TATAATTATTGAAATAATAAAAGGTTCGAGCAGGTTAACAAGCATTAAAAAAAATGTGTTTCATCGTCTACTTATTATTAAAATTGATTGTTCATC
Translation ORF/CDS

1,260 1,280 1,300 1,320 1,340

Phytomonas HART1 AAAAGTATAATTCGTTAGTTGGGTTAAAATCGTTGTAAAGCAGATTTGTTTATATATTTAATGTATATATATAAAATAAATTACATTAGTACGCAA
Translation ORF/CDS

Phytomonas serpens AAAAATATAATTCGTTAGTTGGGTTAAAATCGTTGTAAAGCAGATTTGTTTATATATTTAATGTGTATAAAGCATCAAATTTACATTAGTACGCAA
Translation ORF/CDS

1,360 1,380 1,400 1,420 1,

9S

Phytomonas HART1 GGATCATTTATTAGATAACTTATTATAAATACAAAAAA-----------------GTATAGTCATTTGTTATTATTAATATTAATTTATTTAAAAG
Translation ORF/CDS

5' and 3' ends uncertain

Phytomonas serpens GGATCATTTATTAGCCAT-------------CAAAAAACAGCGAATATTATTAAAATATAGTCAACTGTTATTATTCATACTAATTTTTTTTAAATG
Translation ORF/CDS

440 1,460 1,480 1,500 1,520

Phytomonas HART1 TTTTTATTTTTAATTTTAGTTTATTTATGTA--AAAATTTAATTGTATAGTTTAGGAATAGTTAATAGTAATTTATAATTTTGATTAGATTATTTT
Translation ORF/CDS

Phytomonas serpens TTTTTTATTTTAATAATAGTTTATTTATAGATCATAAATATAAAATAAAGTTTAGGAATAGTTAATAATAATTTATAATTTTGATTAGATTAATTT
Translation ORF/CDS

1,540 1,560 1,580 1,600 1,620

Phytomonas HART1 GTTAATGCTATTATATGGGTGTGGAAAGTTATGTAAAATTAATTACAAATTCAAATAATAAATAGAATGTACTCATAGGTCAGAAATGGATGCCAG
Translation ORF/CDS

Phytomonas serpens GTTAATGCTATTATATGGGTGTGGAAAAT-ATGAAAAATAAATAATATAATAAAATAATAAATAAAATTTAATTATAGGTCAGAAATGGATGCCAG
Translation ORF/CDS

1,640 1,660 1,680 1,700 1,720

Phytomonas HART1 CCGTTGCGGTAATTTCTATGCTTTTAAATATTATACAATAATATTTATGTTATTGTTGCTAGTATATATAACAAACAAAAGTATACA-ACTAATAT
Translation ORF/CDS

Phytomonas serpens CCGTTGCGGTAATTTCTATGCTTTTAAATATTATACAATAATACTTATGATATTGTTAACATACATTATAACAAACAAAAATAAATTCAACAATAT
Translation ORF/CDS

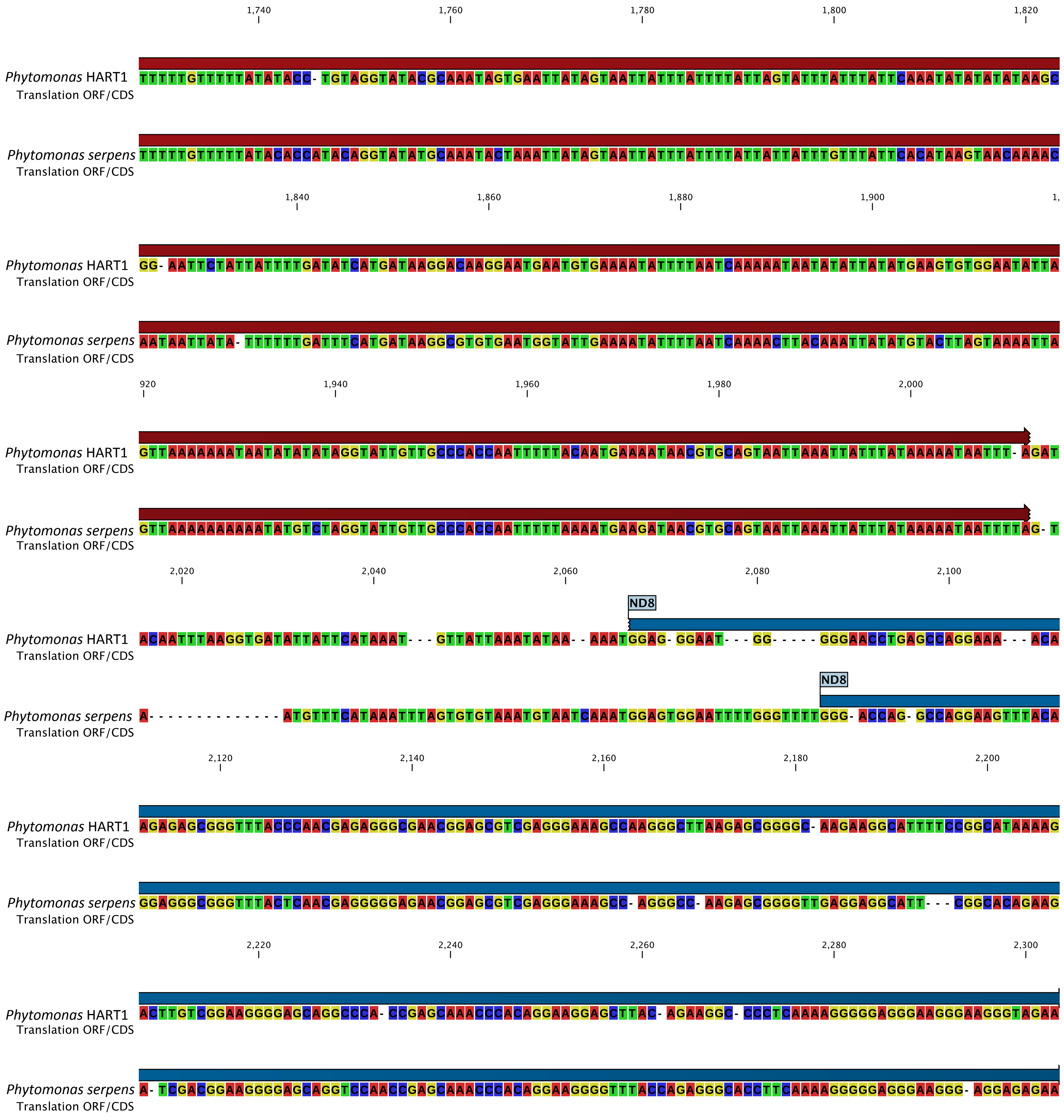

1,740
1,760
1,780
1,800
1,820
Phytomonas HART1
Translation ORF/CDS
Phytomonas serpens
Translation ORF/CDS
1,840
1,860
1,880
1,900
Phytomonas HART1
Translation ORF/CDS
Phytomonas serpens
Translation ORF/CDS
920
1,940
1,960
1,980
2,000
Phytomonas HART1
Translation ORF/CDS
Phytomonas serpens
Translation ORF/CDS
2,020
2,040
2,060
2,080
2,100
ND8
Phytomonas HART1
Translation ORF/CDS
ND8
Phytomonas serpens
Translation ORF/CDS
2,120
2,140
2,160
2,180
2,200
Phytomonas HART1
Translation ORF/CDS
Phytomonas serpens
Translation ORF/CDS
2,220
2,240
2,260
2,280
2,300
Phytomonas HART1
Translation ORF/CDS
Phytomonas serpens
Translation ORF/CDS

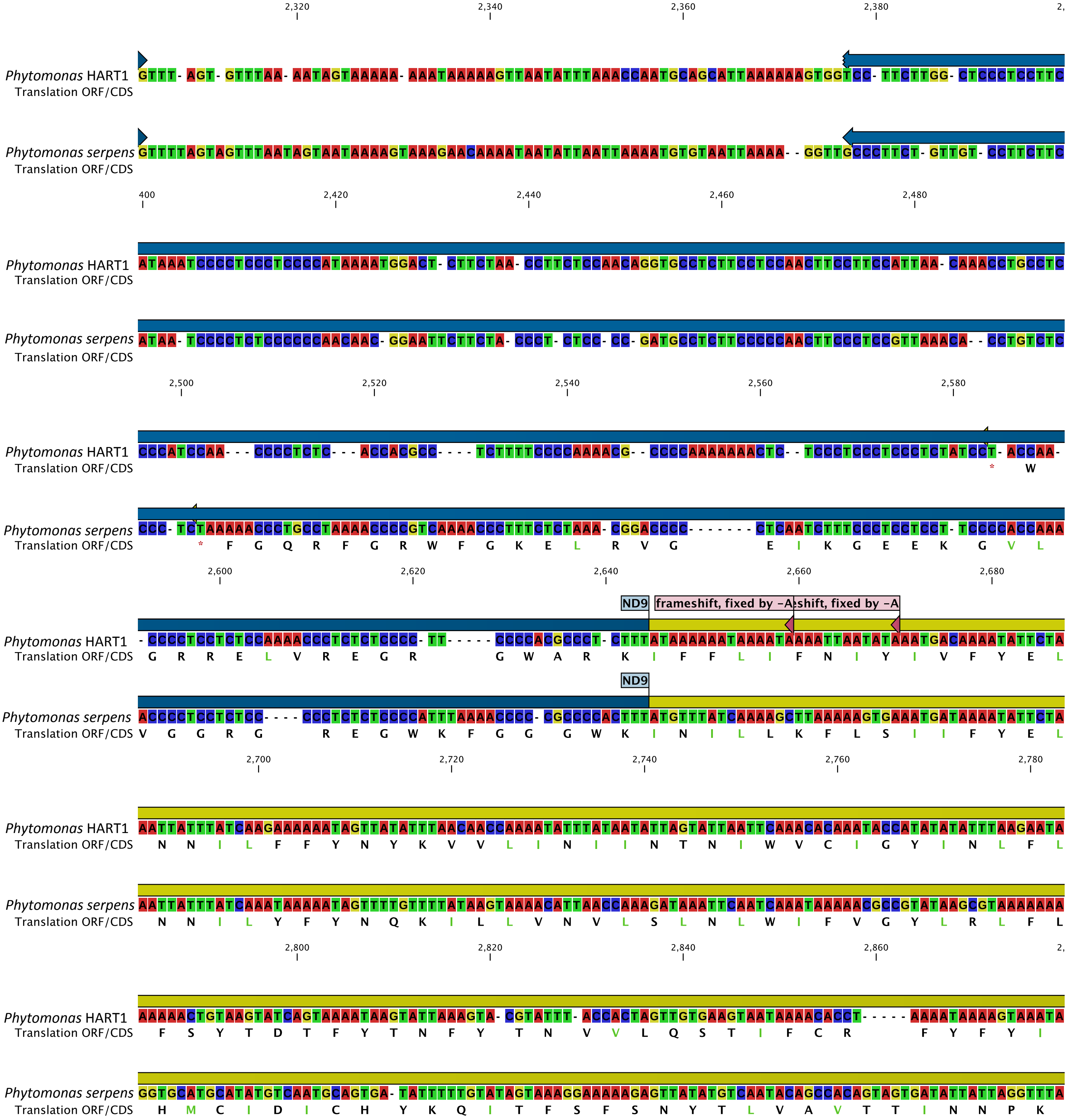
Phytomonas HART1
Translation ORF/CDS
Phytomonas serpens
Translation ORF/CDS
2,320
2,340
2,360
2,380
400
2,420
2,440
2,460
2,480
2,500
2,520
2,540
2,560
2,580
2,600
2,620
2,640
2,660
2,680
ND9
frameshift, fixed by –A
shift, fixed by –A
2,700
2,720
2,740
2,760
2,780
2,800
2,820
2,840
2,860

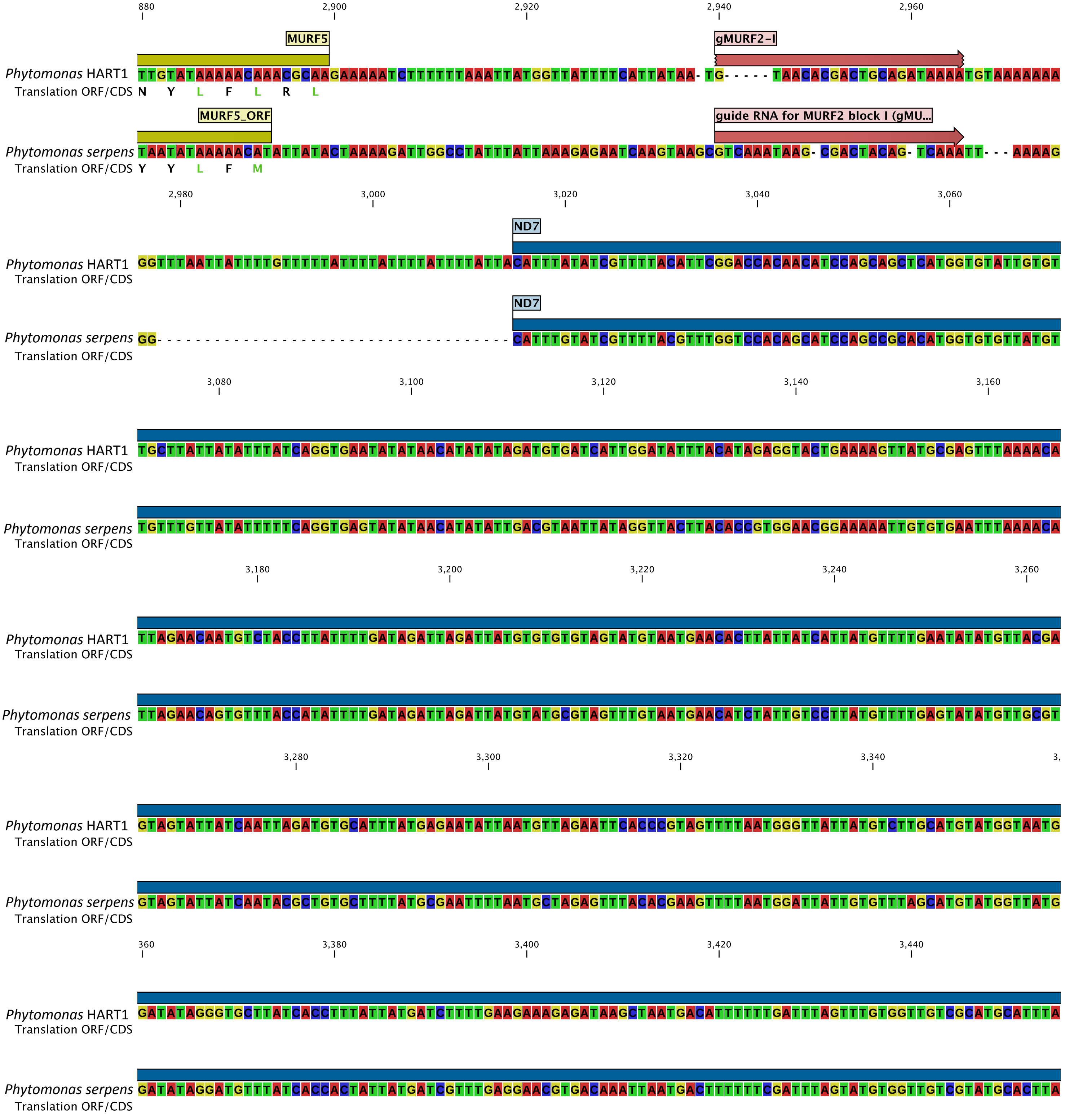

880
2,900
2,920
2,940
2,960
MURF5
gMURF2-I
Phytomonas HART1
Translation ORF/CDS
N Y L F L R L
MURF5_ORF
guide RNA for MURF2 block I (gMU...
Phytomonas serpens
Translation ORF/CDS
Y Y L F M
2,980
3,000
3,020
3,040
3,060
ND7
Phytomonas HART1
Translation ORF/CDS
ND7
Phytomonas serpens
Translation ORF/CDS
3,080
3,100
3,120
3,140
3,160
Phytomonas HART1
Translation ORF/CDS
Phytomonas serpens
Translation ORF/CDS
3,180
3,200
3,220
3,240
3,260
Phytomonas HART1
Translation ORF/CDS
Phytomonas serpens
Translation ORF/CDS
3,280
3,300
3,320
3,340
Phytomonas HART1
Translation ORF/CDS
Phytomonas serpens
Translation ORF/CDS
3,380
3,400
3,420
3,440
Phytomonas HART1
Translation ORF/CDS
Phytomonas serpens
Translation ORF/CDS

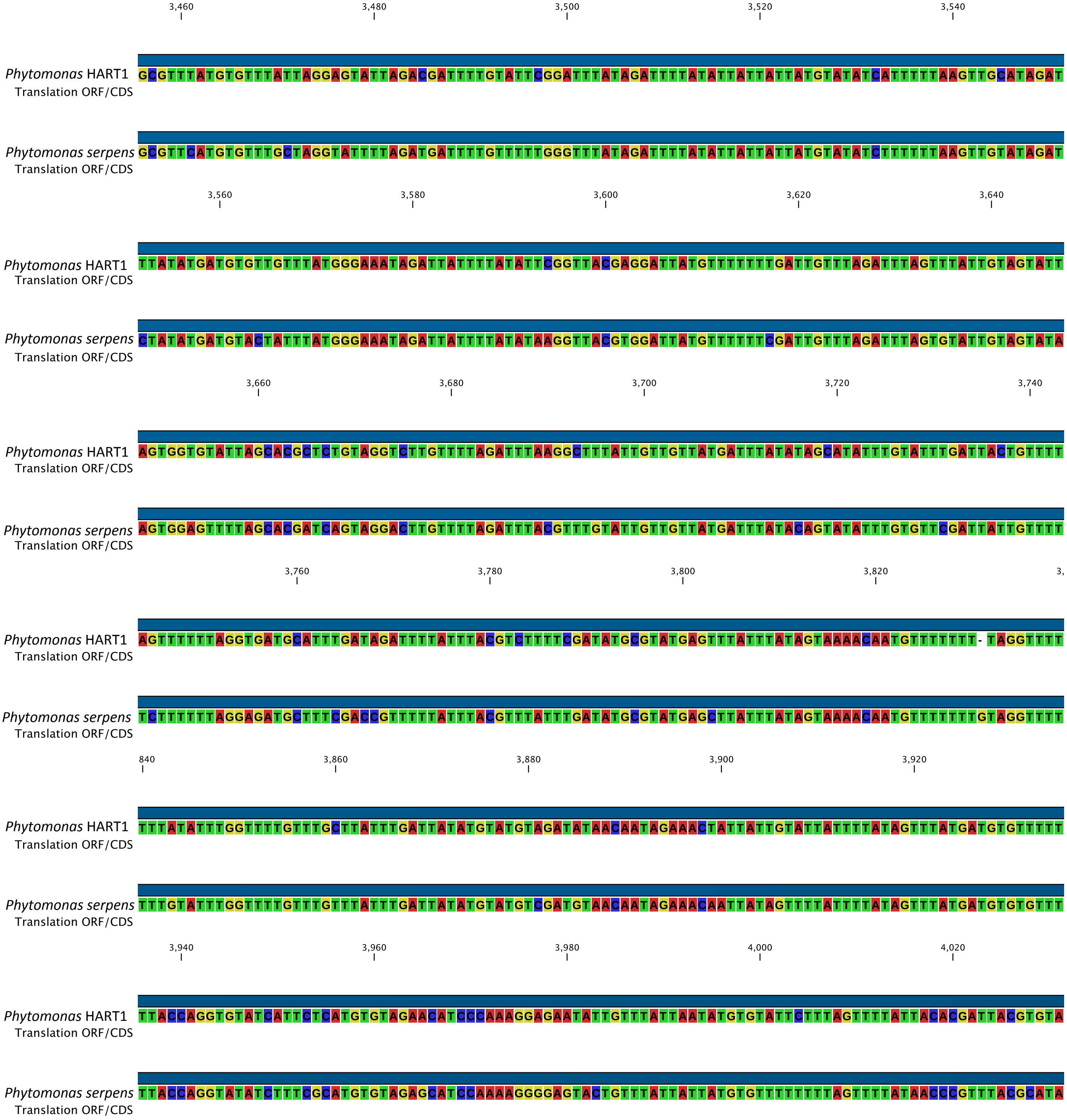
3,460
3,480
3,500
3,520
3,540
Phytomonas HART1
Translation ORF/CDS
Phytomonas serpens
Translation ORF/CDS
3,560
3,580
3,600
3,620
3,640
Phytomonas HART1
Translation ORF/CDS
Phytomonas serpens
Translation ORF/CDS
3,660
3,680
3,700
3,720
3,740
Phytomonas HART1
Translation ORF/CDS
Phytomonas serpens
Translation ORF/CDS
3,760
3,780
3,800
3,820
Phytomonas HART1
Translation ORF/CDS
Phytomonas serpens
Translation ORF/CDS
840
3,860
3,880
3,900
3,920
Phytomonas HART1
Translation ORF/CDS
Phytomonas serpens
Translation ORF/CDS
3,940
3,960
3,980
4,000
4,020
Phytomonas HART1
Translation ORF/CDS
Phytomonas serpens
Translation ORF/CDS

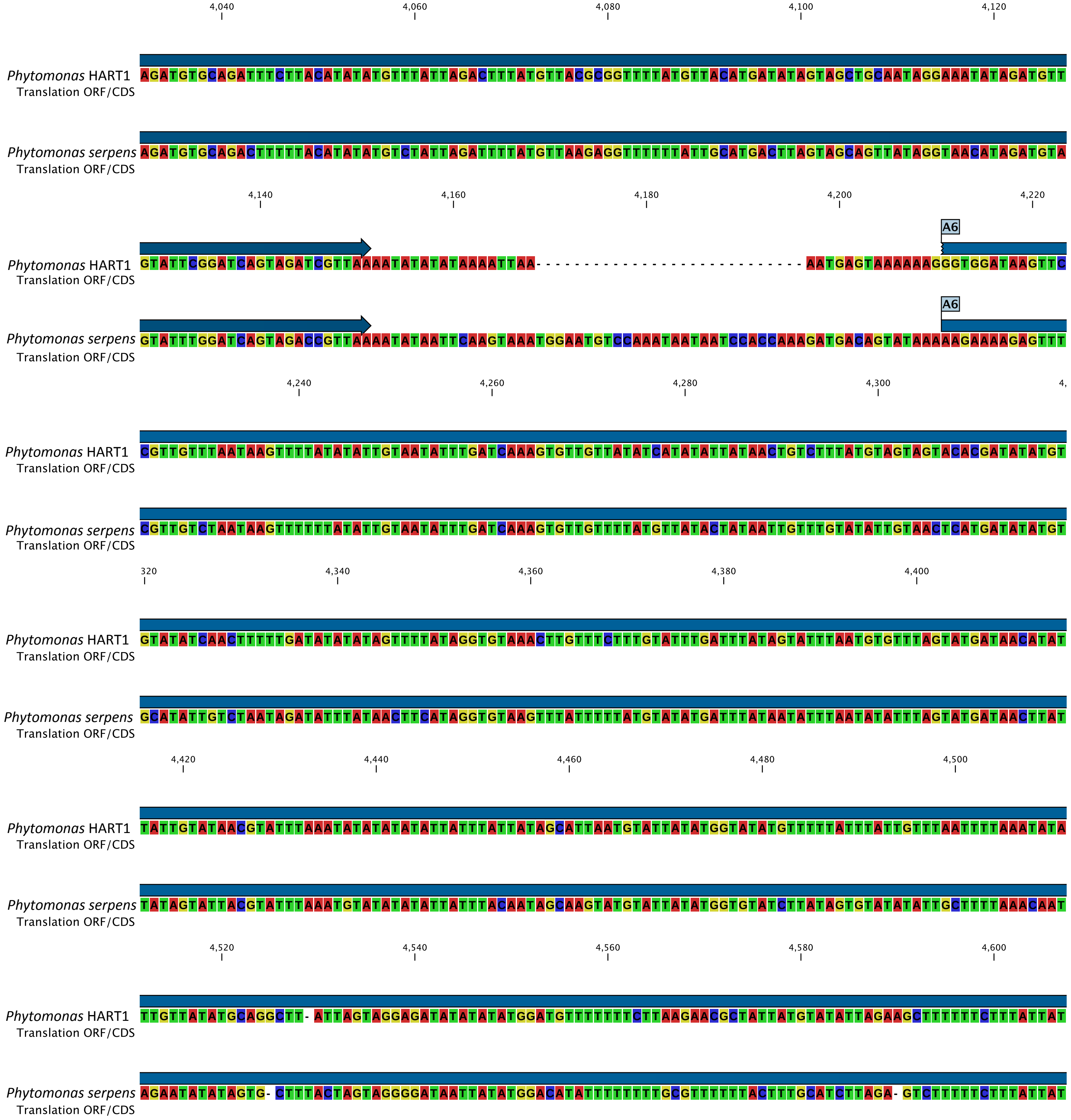
4,040
4,060
4,080
4,100
4,120
Phytomonas HART1
Translation ORF/CDS
Phytomonas serpens
Translation ORF/CDS
4,140
4,160
4,180
4,200
4,220
A6
4,240
4,260
4,280
4,300
320
4,340
4,360
4,380
4,400
4,420
4,440
4,460
4,480
4,500
4,520
4,540
4,560
4,580
4,600

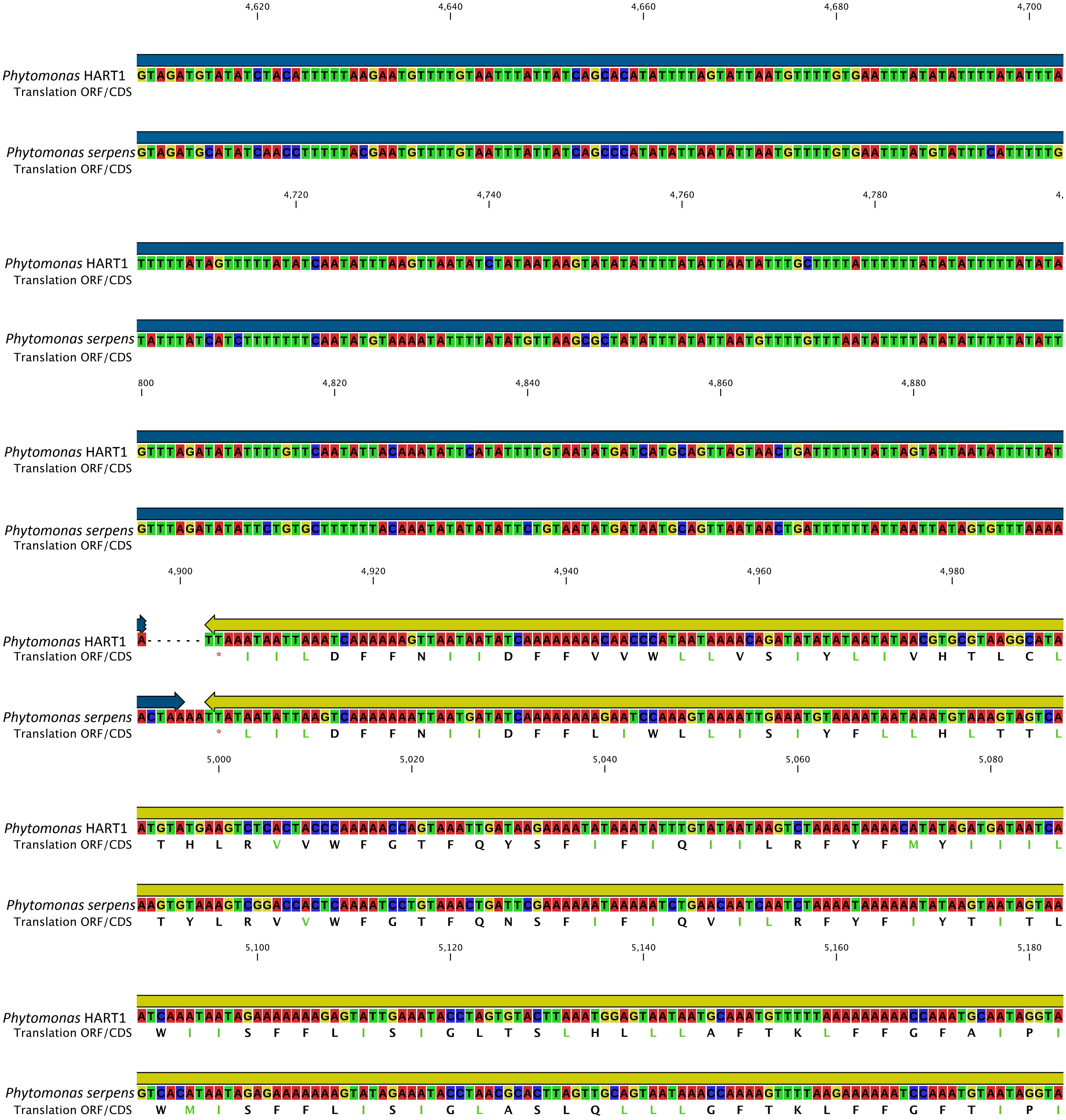
Phytomonas HART1
Translation ORF/CDS
Phytomonas serpens
Translation ORF/CDS
4,620
4,640
4,660
4,680
4,700
4,720
4,740
4,760
4,780
4,820
4,840
4,860
4,880
4,900
4,920
4,940
4,960
4,980
5,000
5,020
5,040
5,060
5,080
5,100
5,120
5,140
5,160
5,180

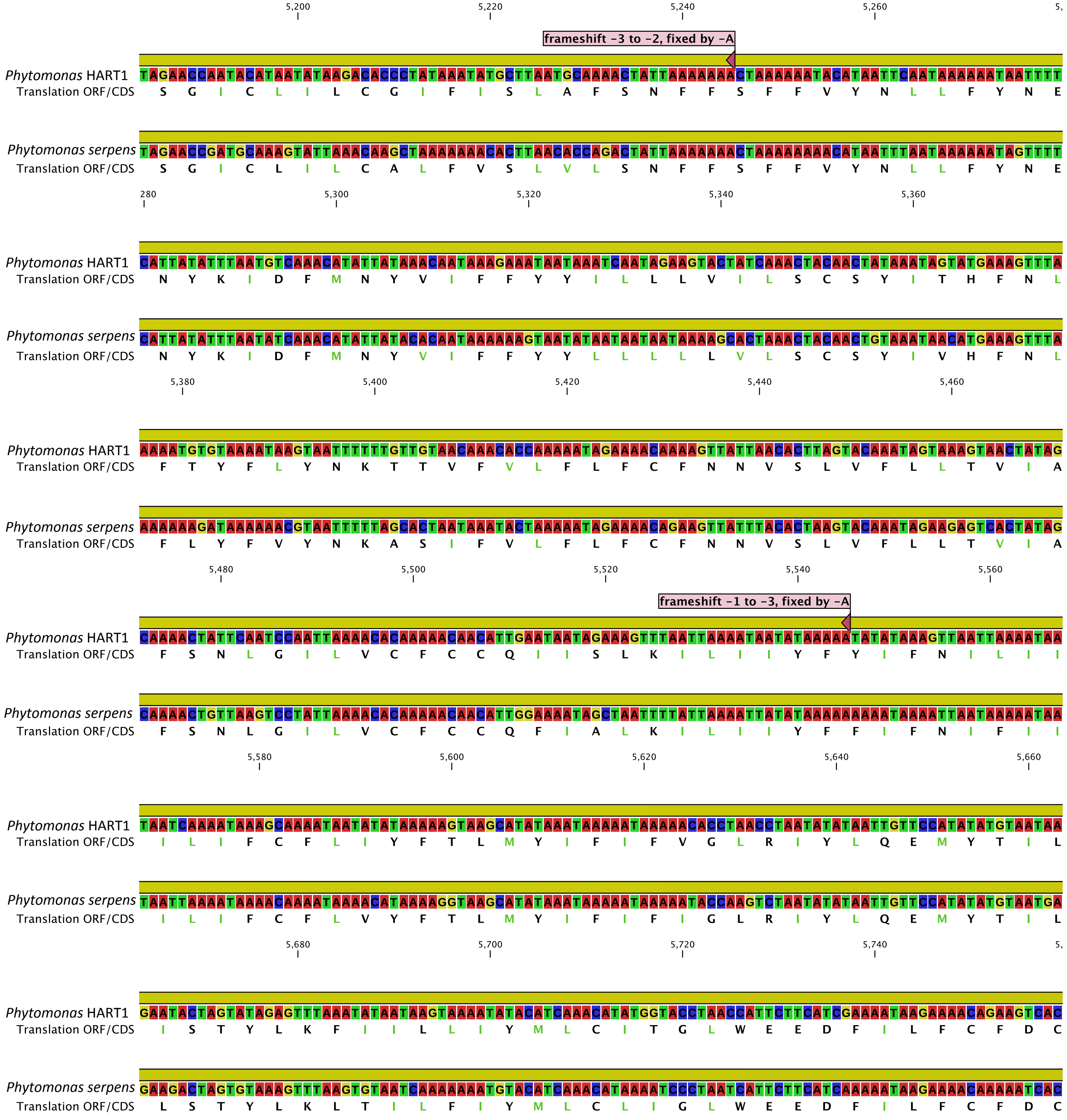
frameshift −3 to −2, fixed by −A
frameshift −1 to −3, fixed by −A
Phytomonas HART1
Phytomonas serpens
Translation ORF/CDS

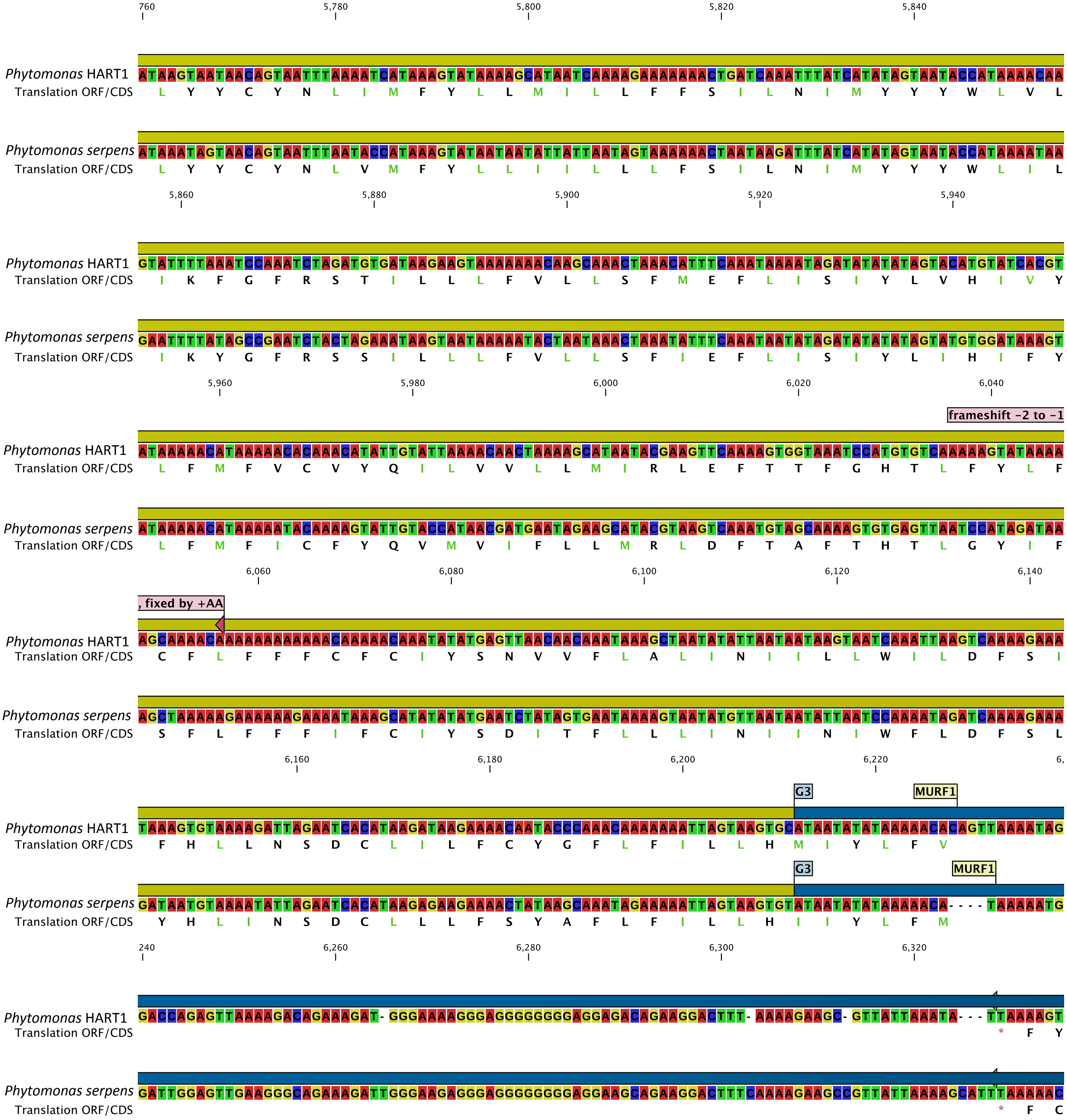
Phytomonas HART1
Phytomonas serpens
Translation ORF/CDS
760
5,780
5,800
5,820
5,840
5,860
5,880
5,900
5,920
5,940
5,960
5,980
6,000
6,020
6,040
frameshift -2 to -1
, fixed by +AA
6,060
6,080
6,100
6,120
6,140
6,160
6,180
6,200
6,220
G3
MURF1
240
6,260
6,280
6,300
6,320

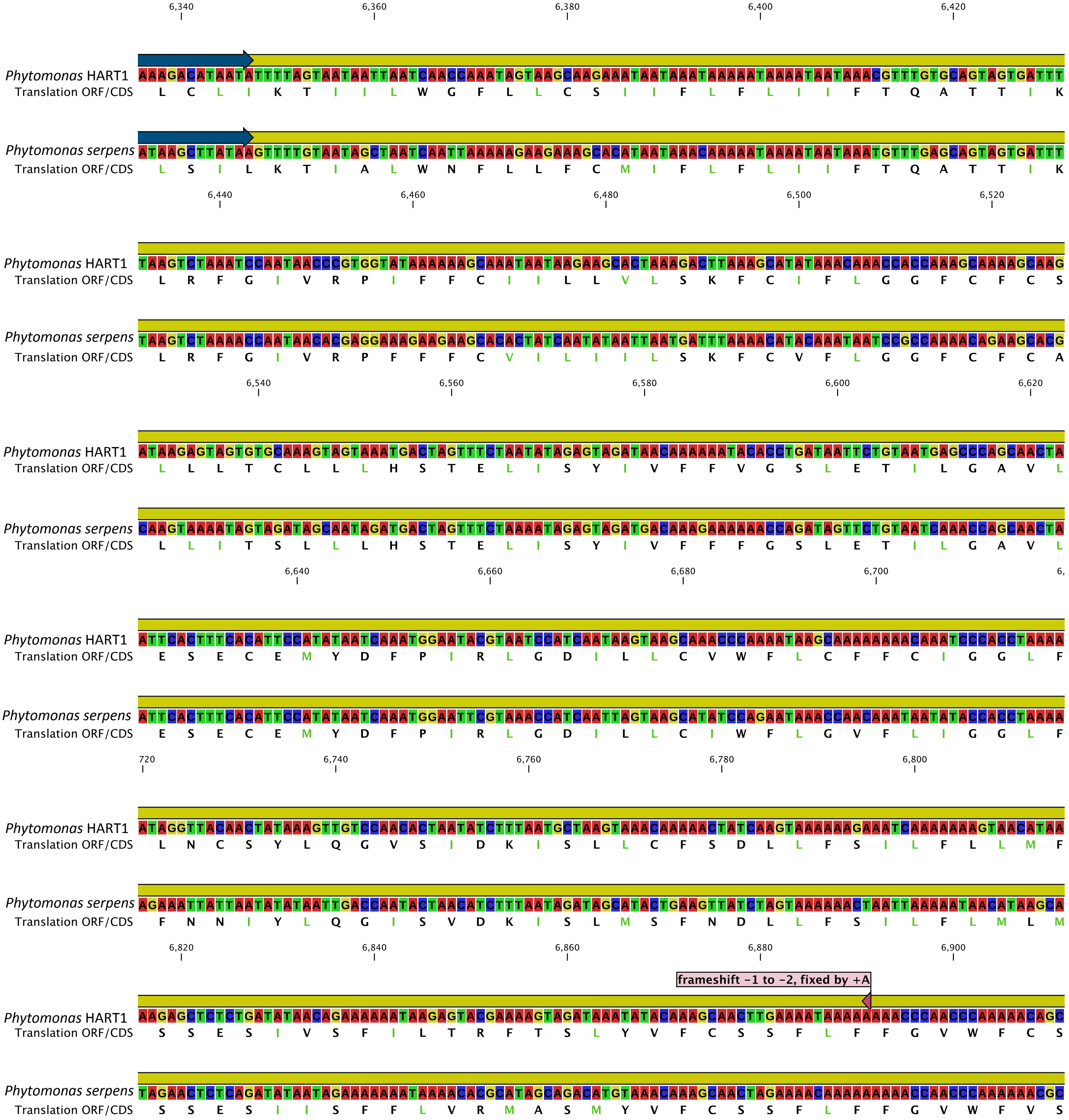
6,340
6,360
6,380
6,400
6,420
Phytomonas HART1
Translation ORF/CDS
Phytomonas serpens
Translation ORF/CDS
6,440
6,460
6,480
6,500
6,520
6,540
6,560
6,580
6,600
6,620
6,640
6,660
6,680
6,700
6,740
6,760
6,780
6,800
6,820
6,840
6,860
6,880
6,900
frameshift −1 to −2, fixed by +A

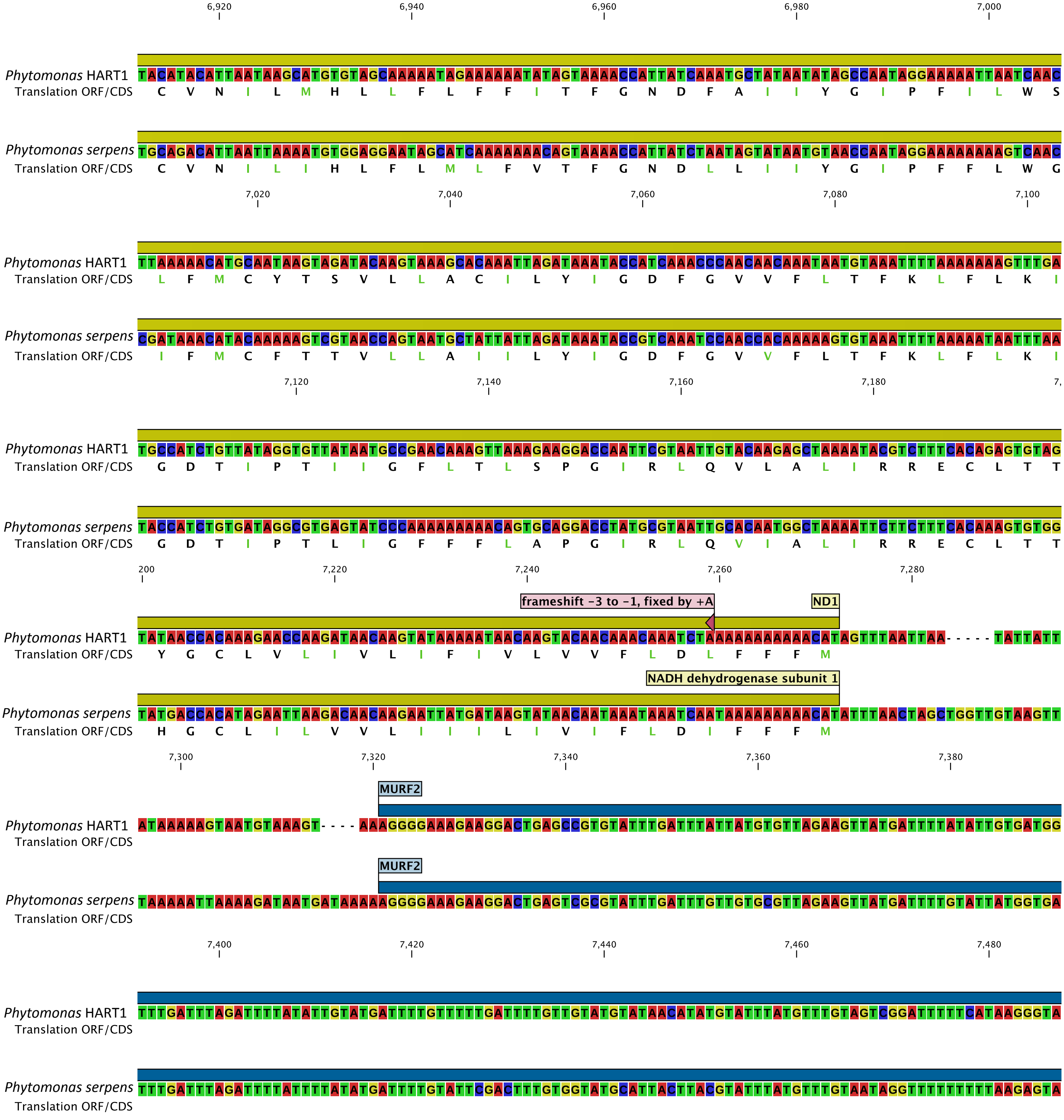
Phytomonas HART1
Phytomonas serpens
Translation ORF/CDS
frameshift −3 to −1, fixed by +A
ND1
NADH dehydrogenase subunit 1
MURF2

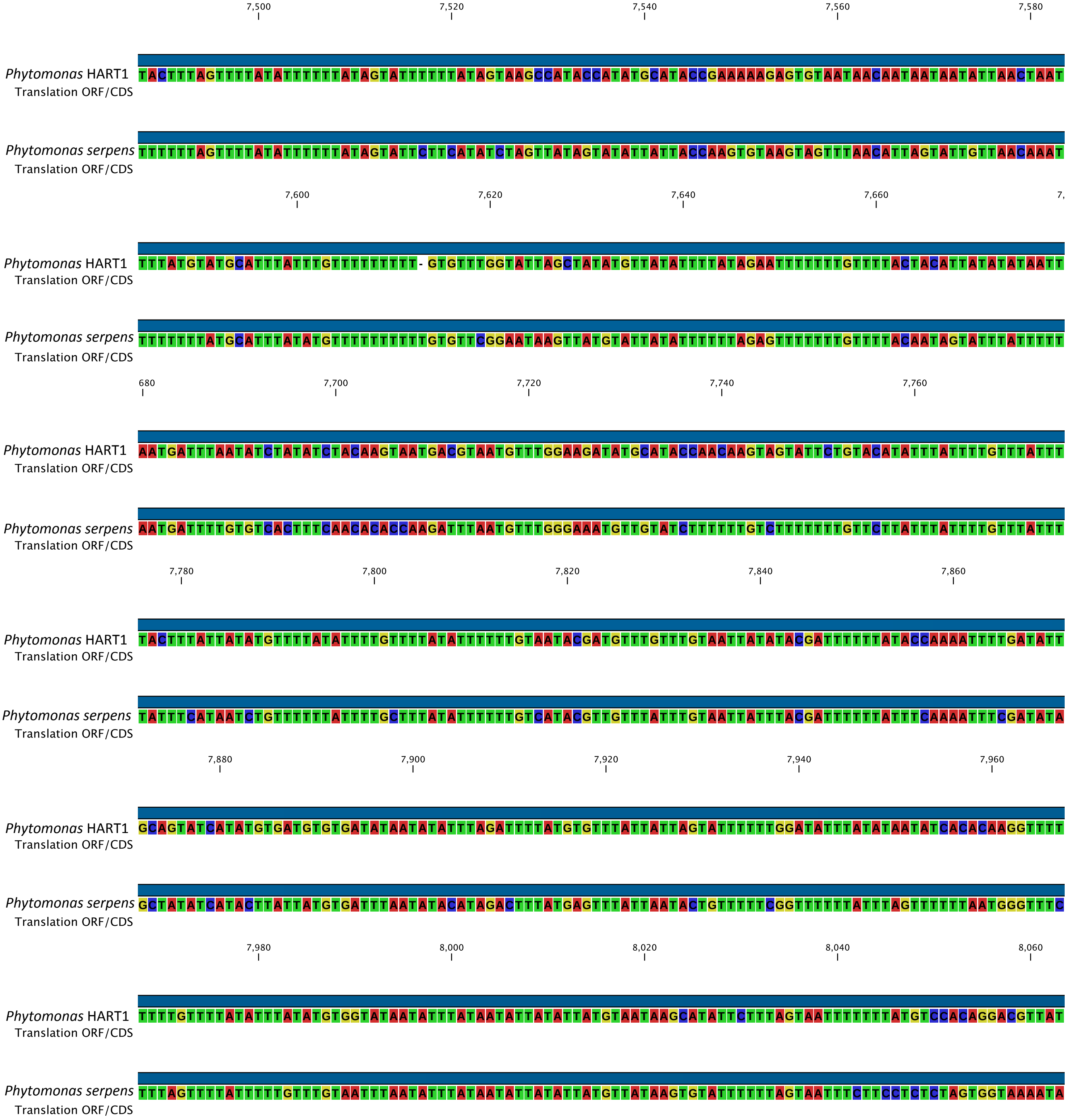

7,500
7,520
7,540
7,560
7,580
Phytomonas HART1
Translation ORF/CDS
Phytomonas serpens
Translation ORF/CDS
7,600
7,620
7,640
7,660
7,680
7,700
7,720
7,740
7,760
7,780
7,800
7,820
7,840
7,860
7,880
7,900
7,920
7,940
7,960
7,980
8,000
8,020
8,040
8,060

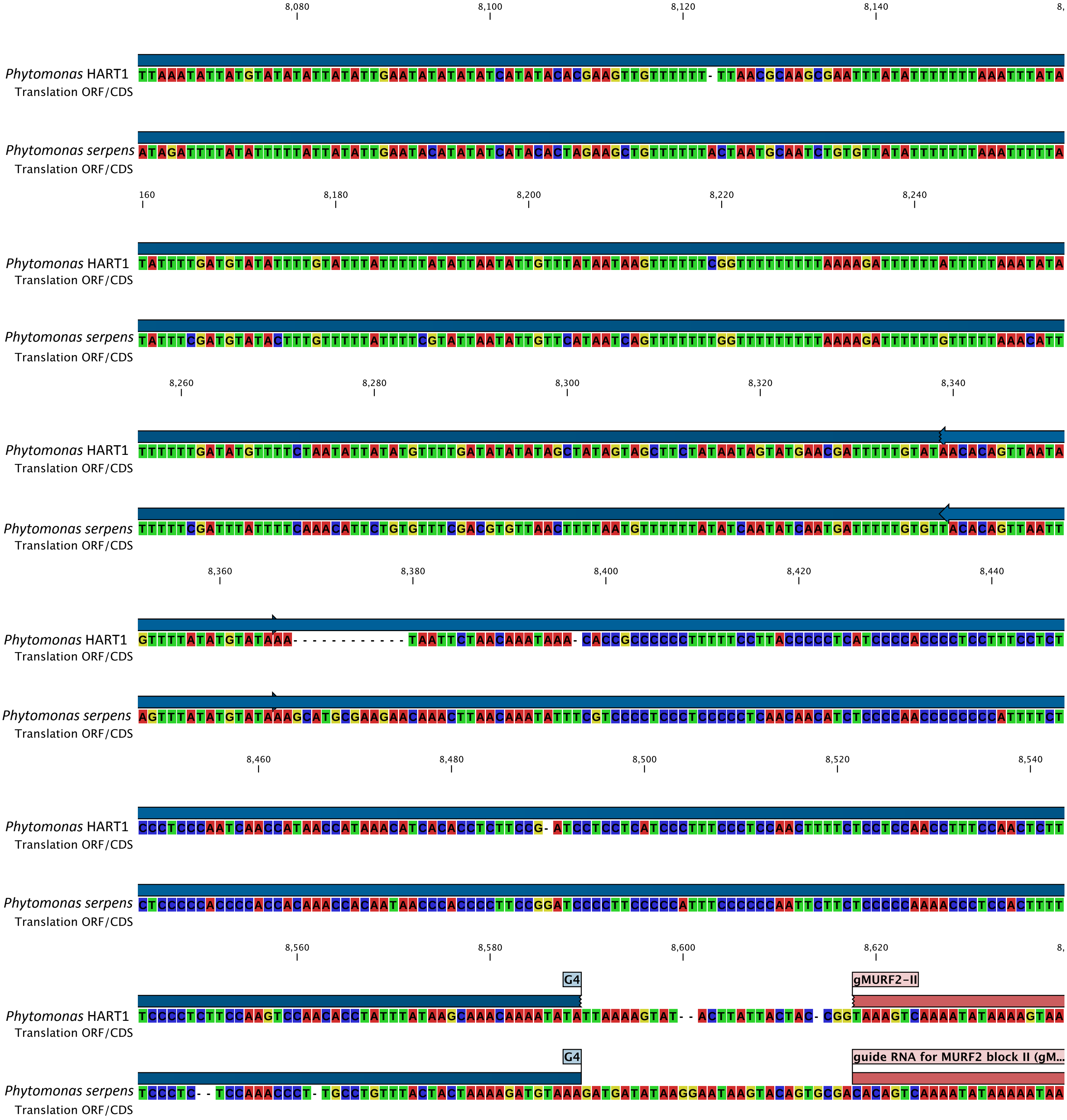
8,080
8,100
8,120
8,140
Phytomonas HART1
TTAAATATTATGTATATATTATATTGAATATATATATCATATACACGAAGTTGTTTTTT- TTAACGCAAGCGAATTTATATTTTTTTAAATTTATA
Translation ORF/CDS
Phytomonas serpens
ATAGATTTTATATTTTTATTATATTGAATACATATATCATACACTAGAAGCTGTTTTTACTAATGCAATCTGTGTTATATTTTTTTAAATTTTTA
Translation ORF/CDS
160
8,180
8,200
8,220
8,240
Phytomonas HART1
TATTTTGATGTATATTTTGTATTTATTTTTATATTAATATTGTTTATAATAAGTTTTTCGGTTTTTTTTTAAAAGATTTTTTATTTTTAAATATA
Translation ORF/CDS
Phytomonas serpens
TATTTCGATGTATACTTTGTTTTTATTTTCGTATTAATATTGTTCATAATCAGTTTTTTGGTTTTTTTTTAAAAGATTTTTTGTTTTTAAACATT
Translation ORF/CDS
8,260
8,280
8,300
8,320
8,340
Phytomonas HART1
TTTTTTGATATGTTTTCTAATATTATATGTTTTGATATATATAGCTATAGTAGCTTCTATAATAGTATGAACGATTTTTGTATAACACAGTTAATA
Translation ORF/CDS
Phytomonas serpens
TTTTTCGATTTATTTTCAAACATTCTGTGTTTCGACGTGTTAACTTTTAATGTTTTTATATCAATATCAATGATTTTTGTGTTACACAGTTAATT
Translation ORF/CDS
8,360
8,380
8,400
8,420
8,440
Phytomonas HART1
GTTTTATATGTATAAA------------TAATTCTAACAAATAAA- CACCGCCCCCCTTTTTCCTTACCCCCTCATCCCCACCCCTCCTTTCCTCT
Translation ORF/CDS
Phytomonas serpens
AGTTTATATGTATAAAGCATGCGAAGAACAAACTTAACAAATATTTCGTCCCCTCCCTCCCCCTCAACAACATCTCCCCAACCCCCCCCATTTTCT
Translation ORF/CDS
8,460
8,480
8,500
8,520
8,540
Phytomonas HART1
CCCTCCCAATCAACCATAACCATAAACATCACACCTCTTCCG- ATCCTCCTCATCCCTTTCCCTCCAACTTTTCTCCTCCAACCTTTCCAACTCTT
Translation ORF/CDS
Phytomonas serpens
CTCCCCCACCCCACCACAAACCACAATAACCCACCCCTTCCGGATCCCCTTCCCCCATTTCCCCCCAATTCTTCTCCCCCAAAACCCTCCACTTTT
Translation ORF/CDS
8,560
8,580
8,600
8,620
G4
gMURF2–II
Phytomonas HART1
TCCCTCTTCCAAGTCCAACACCTATTTATAAGCAAACAAAATATATTAAAAGTAT- - ACTTATTACTAC- CGGTAAAGTCAAAATATAAAAGTAA
Translation ORF/CDS
G4
guide RNA for MURF2 block II (gM...
Phytomonas serpens
TCCCTC- - TCCAAACCCT- TGCCTGTTTACTACTAAAAGATGTAAAGATGATATAAGGAATAAGTACAGTGCGACACAGTCAAAATATAAAAATAA
Translation ORF/CDS

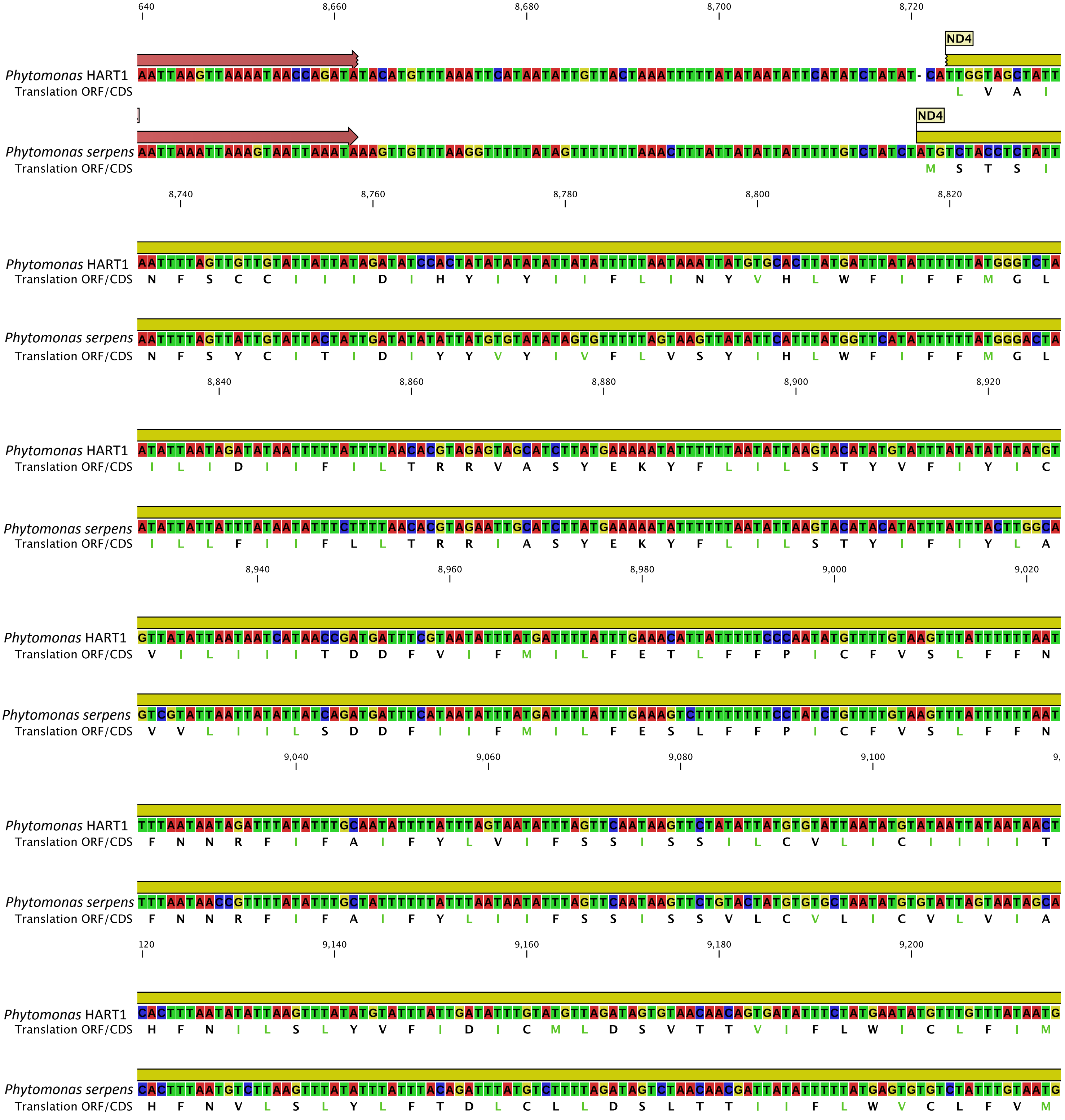
640
8,660
8,680
8,700
8,720
ND4
Phytomonas HART1
Translation ORF/CDS
Phytomonas serpens
Translation ORF/CDS
8,740
8,760
8,780
8,800
8,820
8,840
8,860
8,880
8,900
8,920
8,940
8,960
8,980
9,000
9,020
9,040
9,060
9,080
9,100
120
9,140
9,160
9,180
9,200

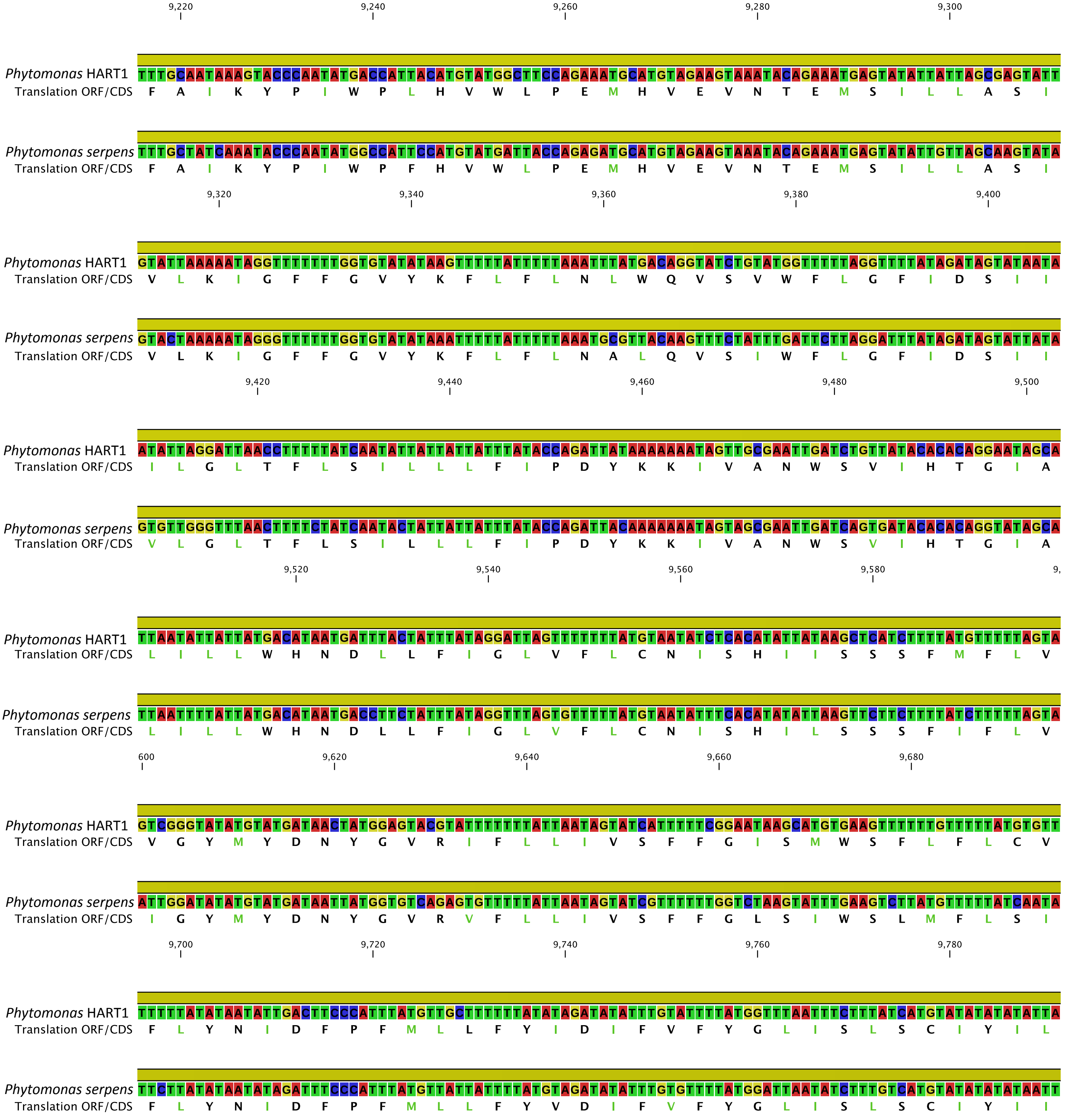
9,220
9,240
9,260
9,280
9,300
Phytomonas HART1 TTTGCAATAAAGTACCCAATATGACCATTACATGTATGGCTTCCAGAAATGCATGTAGAAGTAAATACAGAAATGAGTATATTATTAGCGAGTATT
Translation ORF/CDS F A I K Y P I W P L H V W L P E M H V E V N T E M S I L L A S I
Phytomonas serpens TTTGCTATCAAATACCCAATATGGCCATTCCATGTATGATTACCAGAGATGCATGTAGAAGTAAATACAGAAATGAGTATATTGTTAGCAAGTATA
Translation ORF/CDS F A I K Y P I W P F H V W L P E M H V E V N T E M S I L L A S I
9,320
9,340
9,360
9,380
9,400
Phytomonas HART1 GTATTAAAAATAGGTTTTTTTGGTGTATATAAGTTTTTATTTTTAAATTTATGACAGGTATCTGTATGGTTTTTAGGTTTTATAGATAGTATAATA
Translation ORF/CDS V L K I G F F G V Y K F L F L N L W Q V S V W F L G F I D S I I
Phytomonas serpens GTACTAAAAATAGGGTTTTTTGGTGTATATAAATTTTTATTTTTAAATGCGTTACAAGTTTCTATTTGATTCTTAGGATTTATAGATAGTATTATA
Translation ORF/CDS V L K I G F F G V Y K F L F L N A L Q V S I W F L G F I D S I I
9,420
9,440
9,460
9,480
9,500
Phytomonas HART1 ATATTAGGATTAACCTTTTTATCAATATTATTATTATTTATACCAGATTATAAAAAAATAGTTGCGAATTGATCTGTTATACACACAGGAATAGCA
Translation ORF/CDS I L G L T F L S I L L L F I P D Y K K I V A N W S V I H T G I A
Phytomonas serpens GTGTTGGGTTTAACTTTTCTATCAATACTATTATTATTTATACCAGATTACAAAAAAATAGTAGCGAATTGATCAGTGATACACACAGGTATAGCA
Translation ORF/CDS V L G L T F L S I L L L F I P D Y K K I V A N W S V I H T G I A
9,520
9,540
9,560
9,580
9,
Phytomonas HART1 TTAATATTATTATGACATAATGATTTACTATTTATAGGATTAGTTTTTTTATGTAATATCTCACATATTATAAGCTCATCTTTTATGTTTTTAGTA
Translation ORF/CDS L I L L W H N D L L F I G L V F L C N I S H I I S S S F M F L V
Phytomonas serpens TTAATTTTATTATGACATAATGACCTTCTATTTATAGGTTTAGTGTTTTTATGTAATATTTCACATATATTAAGTTCTTCTTTTATCTTTTTAGTA
Translation ORF/CDS L I L L W H N D L L F I G L V F L C N I S H I L S S S F I F L V
600
9,620
9,640
9,660
9,680
Phytomonas HART1 GTCGGGTATATGTATGATAACTATGGAGTACGTATTTTTTTATTAATAGTATCATTTTTCGGAATAAGCATGTGAAGTTTTTTGTTTTTATGTGTT
Translation ORF/CDS V G Y M Y D N Y G V R I F L L I V S F F G I S M W S F L F L C V
Phytomonas serpens ATTGGATATATGTATGATAATTATGGTGTCAGAGTGTTTTTATTAATAGTATCGTTTTTTGGTCTAAGTATTTGAAGTCTTATGTTTTTATCAATA
Translation ORF/CDS I G Y M Y D N Y G V R V F L L I V S F F G L S I W S L M F L S I
9,700
9,720
9,740
9,760
9,780
Phytomonas HART1 TTTTTATATAATATTGACTTCCCATTTATGTTGCTTTTTTATATAGATATATTTGTATTTTATGGTTTAATTTCTTTATCATGTATATATATATTA
Translation ORF/CDS F L Y N I D F P F M L L F Y I D I F V F Y G L I S L S C I Y I L
Phytomonas serpens TTCTTATATAATATAGATTTCCCATTTATGTTATTATTTTATGTAGATATATTTGTGTTTTATGGATTAATATCTTTGTCATGTATATATATAATT
Translation ORF/CDS F L Y N I D F P F M L L F Y V D I F V F Y G L I S L S C I Y I I

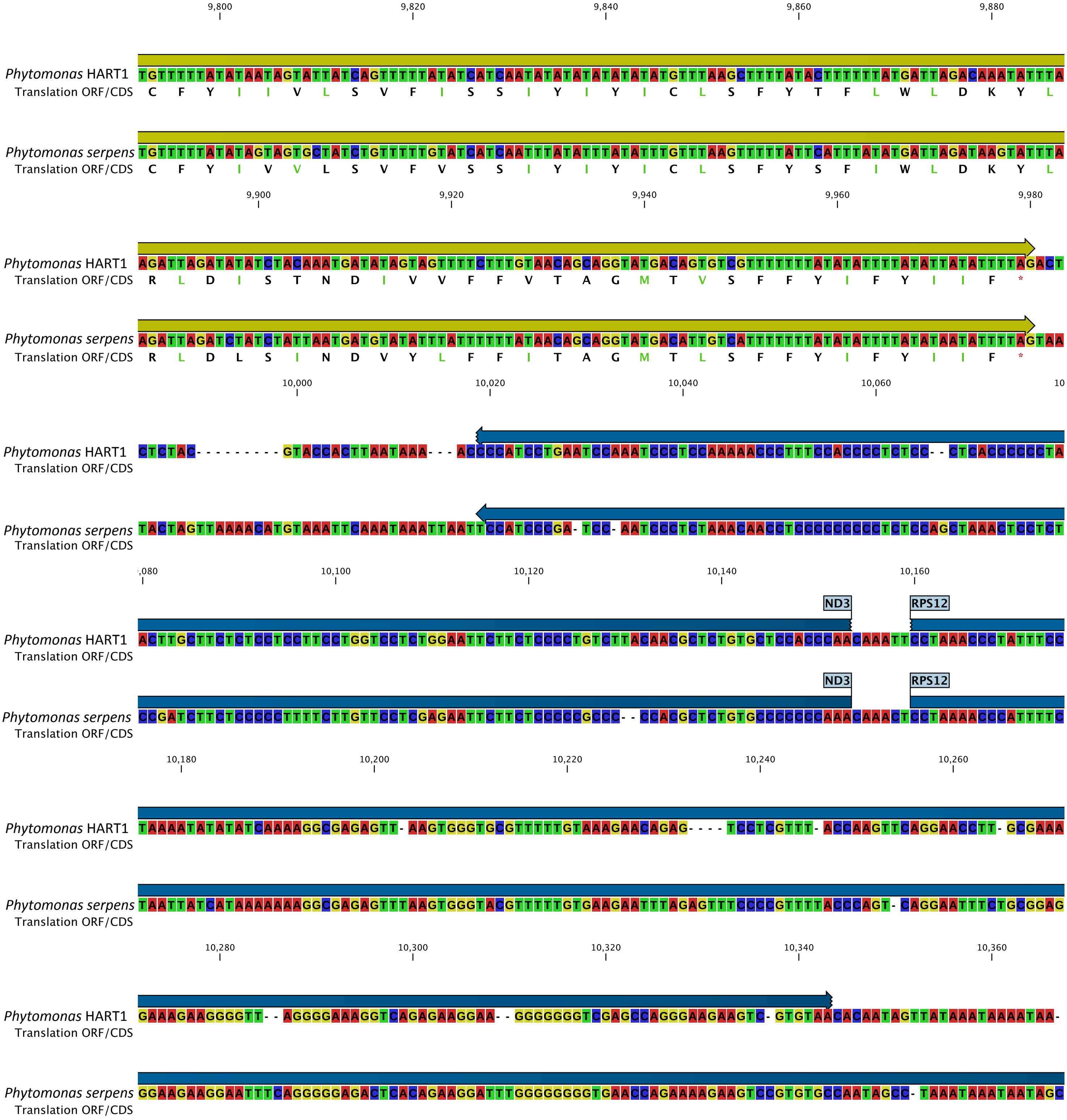
9,800
9,820
9,840
9,860
9,880
Phytomonas HART1
Translation ORF/CDS
Phytomonas serpens
Translation ORF/CDS
9,900
9,920
9,940
9,960
9,980
10,000
10,020
10,040
10,060
10,080
10,100
10,120
10,140
10,160
ND3
RPS12
10,180
10,200
10,220
10,240
10,260
10,280
10,300
10,320
10,340
10,360

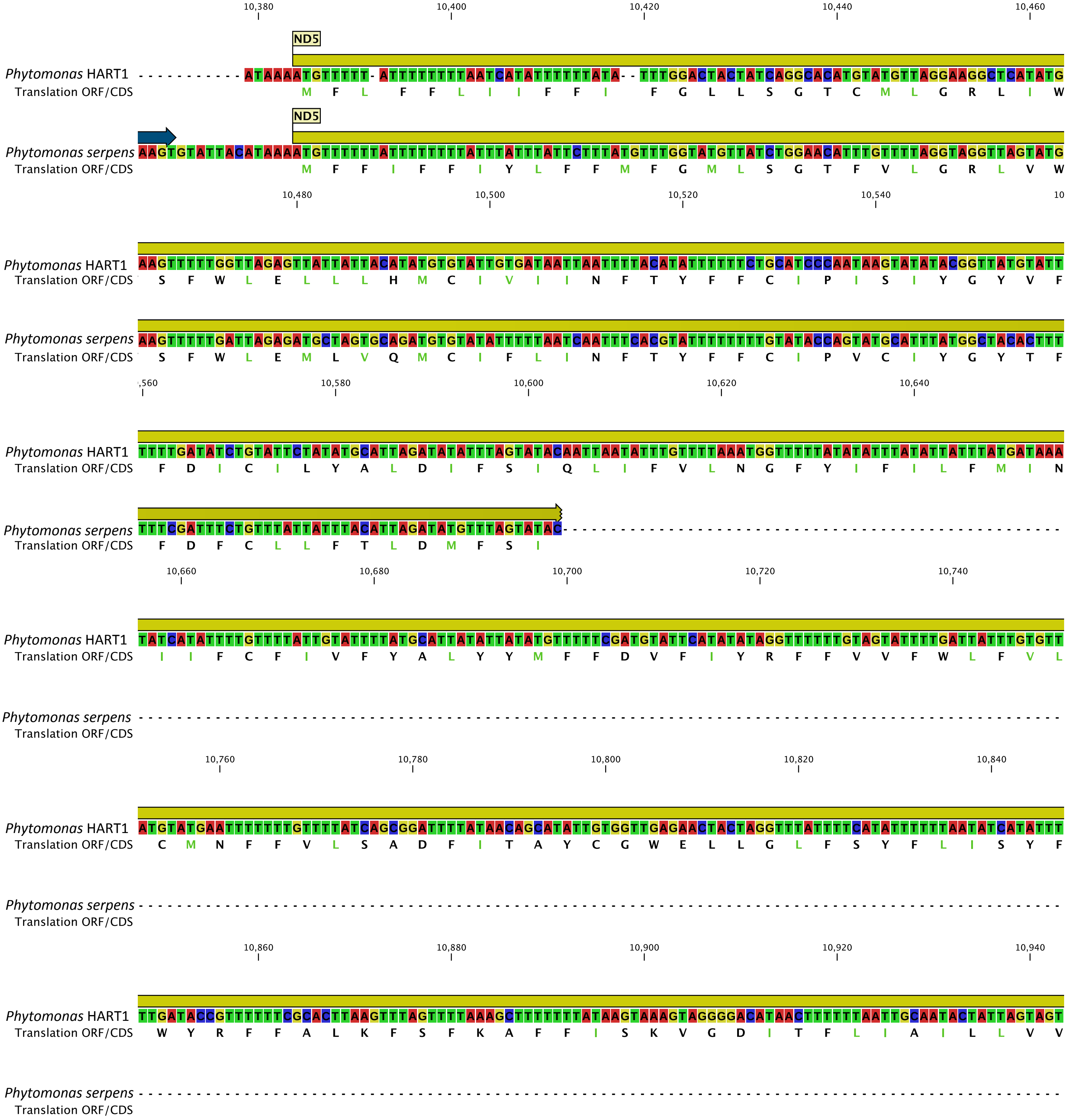
ND5
Phytomonas HART1
Phytomonas serpens
Translation ORF/CDS
10,380
10,400
10,420
10,440
10,460
10,480
10,500
10,520
10,540
10,580
10,600
10,620
10,640
10,660
10,680
10,700
10,720
10,740
10,760
10,780
10,800
10,820
10,840
10,860
10,880
10,900
10,920
10,940

10,960 10,980 11,000 11,020 11

Phytomonas HART1 ATATAATCATGGTTATAGTTGTGTCATATATCTGTTAGTCACATTTTTTAATGTAGATTACTATTGTATGATATGAACACTATTTATGTTATTAAT

Translation ORF/CDS Y N H G Y S C V I Y L L V T F F N V D Y Y C M I W T L F M L L I

Phytomonas serpens --

Translation ORF/CDS

,040 11,060 11,080 11,100 11,120

Phytomonas HART1 ATGTGCTTTTACTAAGTCAACACAATTTGGTTTACATATATGATTACCAGATGCAATGGAGGGTCCTATCCCAGTATCAGCGTTAATACATGCAGC

Translation ORF/CDS C A F T K S T Q F G L H I W L P D A M E G P I P V S A L I H A A

Phytomonas serpens --

Translation ORF/CDS

11,140 11,160 11,180 11,200 11,220

Phytomonas HART1 TACATTAGTTATATGTGGTATTGTATTAATGAGTTTCTTATTTTGATGTTATGATTTCTGAATATGTTGTTTTTACCCATTATTATATTGATCAAG

Translation ORF/CDS T L V I C G I V L M S F L F W C Y D F W I C C F Y P L L Y W S S

Phytomonas serpens --

Translation ORF/CDS

11,240 11,260 11,280 11,300 11,320

Phytomonas HART1 TTTAATAGTAGTAGTGGTGGCTATATCAGTAACATATAATTTTGATGTAAAACGTTATATAGCATTTAGCACAATATGCCAAATATCATTTTCTAT

Translation ORF/CDS L I V V V V A I S V T Y N F D V K R Y I A F S T I C Q I S F S M

Phytomonas serpens --

Translation ORF/CDS

11,340 11,360 11,380 11,400 11,420

Phytomonas HART1 GTTTTGTTGTTTAATGATAGACATATATGTAGGAGCATTATTTTTCTGTTATCATATGTGTTATAAAGCAGCATTATTTATTATAATAGGGGTATG

Translation ORF/CDS F C C L M I D I Y V G A L F F C Y H M C Y K A A L F I I I G V W

Phytomonas serpens --

Translation ORF/CDS

11,440 11,460 11,480 11,500 11

Phytomonas HART1 GATCCATCTTTTTTATGGATTACAAGATATAAGGTGTTTCTTCTTTTTCCATTTTTGTGGATGTGTGTTAGCAAAGGTATTAGTAGTATTCGCATT

Translation ORF/CDS I H L F Y G L Q D I R C F F F F H F C G C V L A K V L V V F A L

Phytomonas serpens --

Translation ORF/CDS

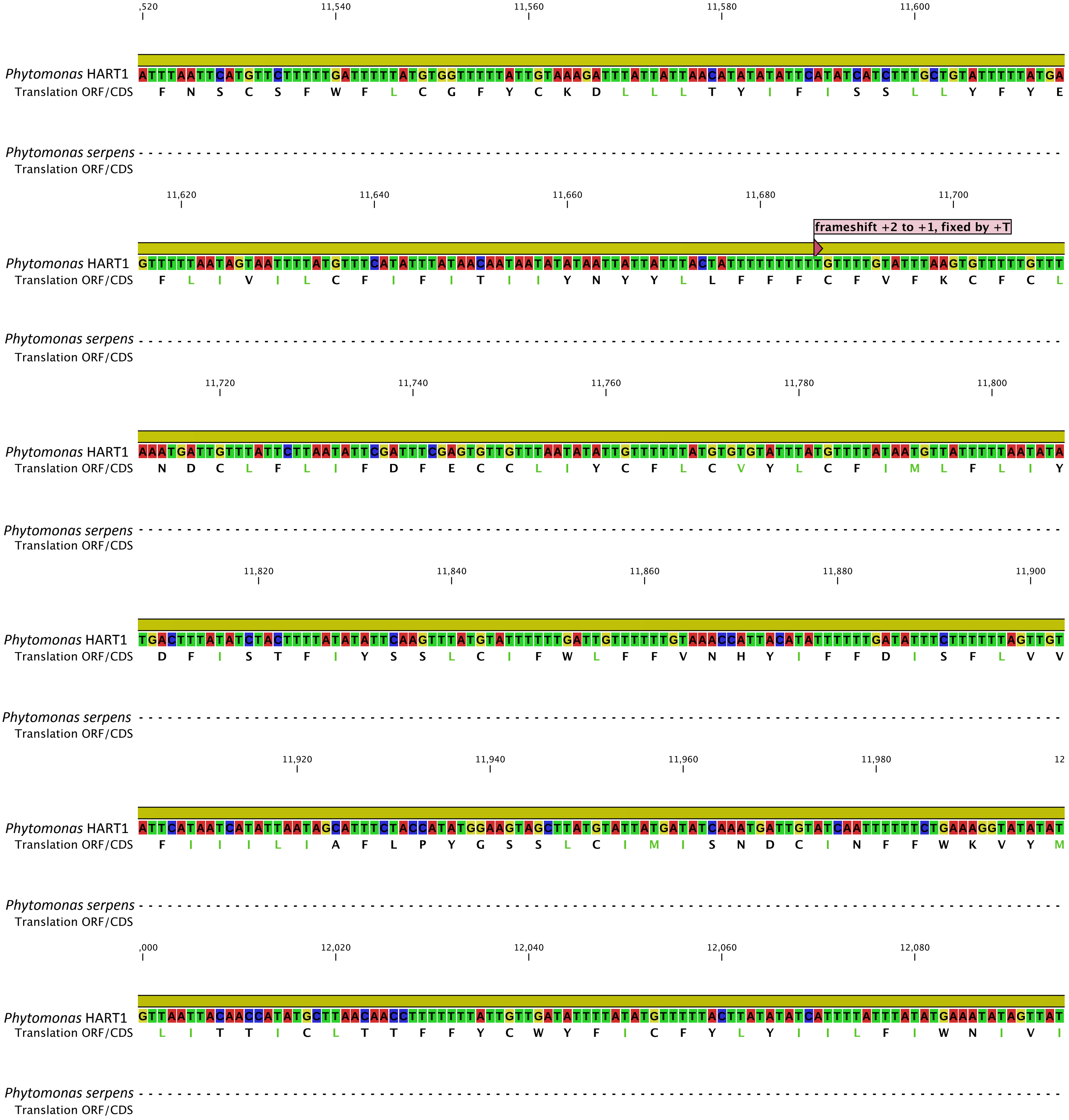
,520
11,540
11,560
11,580
11,600
Phytomonas HART1
ATTTAATTCATGTTCTTTTTGATTTTTATGTGGTTTTTATTGTAAAGATTTATTATTAACATATATATTCATATCATCTTTGCTGTATTTTTATGA
Translation ORF/CDS
F N S C S F W F L C G F Y C K D L L L T Y I F I S S L L Y F Y E
Phytomonas serpens
Translation ORF/CDS
11,620
11,640
11,660
11,680
11,700
frameshift +2 to +1, fixed by +T
Phytomonas HART1
GTTTTTAATAGTAATTTTATGTTTCATATTTATAACAATAATATATAATTATTATTTACTATTTTTTTTTTGTTTTGTATTTAAGTGTTTTTGTTT
Translation ORF/CDS
F L I V I L C F I F I T I I Y N Y Y L L F F F C F V F K C F C L
Phytomonas serpens
Translation ORF/CDS
11,720
11,740
11,760
11,780
11,800
Phytomonas HART1
AAATGATTGTTTATTCTTAATATTCGATTTCGAGTGTTGTTTAATATATTGTTTTTTATGTGTGTATTTATGTTTTATAATGTTATTTTTAATATA
Translation ORF/CDS
N D C L F L I F D F E C C L I Y C F L C V Y L C F I M L F L I Y
Phytomonas serpens
Translation ORF/CDS
11,820
11,840
11,860
11,880
11,900
Phytomonas HART1
TGACTTTATATCTACTTTTATATATTCAAGTTTATGTATTTTTTGATTGTTTTTTGTAAACCATTACATATTTTTTGATATTTCTTTTTTAGTTGT
Translation ORF/CDS
D F I S T F I Y S S L C I F W L F F V N H Y I F F D I S F L V V
Phytomonas serpens
Translation ORF/CDS
11,920
11,940
11,960
11,980
12
Phytomonas HART1
ATTCATAATCATATTAATAGCATTTCTACCATATGGAAGTAGCTTATGTATTATGATATCAAATGATTGTATCAATTTTTTCTGAAAGGTATATAT
Translation ORF/CDS
F I I I L I A F L P Y G S S L C I M I S N D C I N F F W K V Y M
Phytomonas serpens
Translation ORF/CDS
,000
12,020
12,040
12,060
12,080
Phytomonas HART1
GTTAATTACAACCATATGCTTAACAACCTTTTTTTATTGTTGATATTTTATATGTTTTTACTTATATATCATTTTATTTATATGAAATATAGTTAT
Translation ORF/CDS
L I T T I C L T T F F Y C W Y F I C F Y L Y I I L F I W N I V I
Phytomonas serpens
Translation ORF/CDS

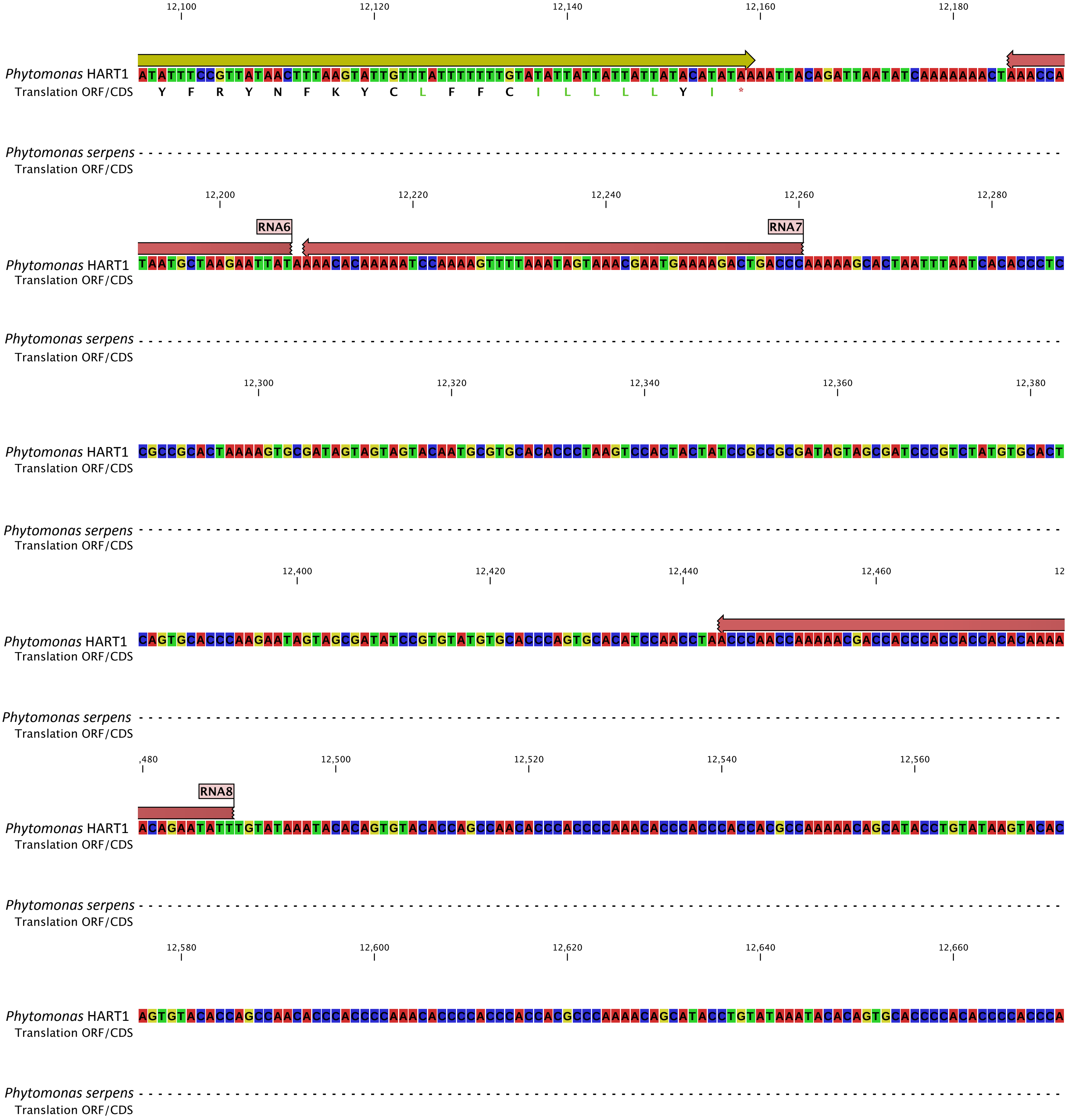
12,100
12,120
12,140
12,160
12,180
Phytomonas HART1 ATATTTCCGTTATAACTTTAAGTATTGTTTATTTTTTTGTATATTATTATTATTATATACATATAAAATTACAGATTAATATCAAAAAAACTAAACCA
Translation ORF/CDS Y F R Y N F K Y C L F F C I L L L L Y I *
Phytomonas serpens
Translation ORF/CDS
12,200
12,220
12,240
12,260
12,280
RNA6
RNA7
Phytomonas HART1 TAATGCTAAGAATTATAAAACACAAAAATCCAAAAGTTTTAAATAGTAAACGAATGAAAAGACTGACCCAAAAAGCACTAATTTAATCACACCCTC
Translation ORF/CDS
Phytomonas serpens
Translation ORF/CDS
12,300
12,320
12,340
12,360
12,380
Phytomonas HART1 CGCCGCACTAAAAGTGCGATAGTAGTAGTACAATGCGTGCACACCCTAAGTCCACTACTATCCGCCGCGATAGTAGCGATCCCGTCTATGTGCACT
Translation ORF/CDS
Phytomonas serpens
Translation ORF/CDS
12,400
12,420
12,440
12,460
12
Phytomonas HART1 CAGTGCACCCAAGAATAGTAGCGATATCCGTGTATGTGCACCCAGTGCACATCCAACCTAACCCAACCAAAAACGACCACCCACCACCACACAAAA
Translation ORF/CDS
Phytomonas serpens
Translation ORF/CDS
,480
12,500
12,520
12,540
12,560
RNA8
Phytomonas HART1 ACAGAATATTTGTATAAATACACAGTGTACACCAGCCAACACCCACCCCAAACACCCACCCACCACGCCAAAAACAGCATACCTGTATAAGTACAC
Translation ORF/CDS
Phytomonas serpens
Translation ORF/CDS
12,580
12,600
12,620
12,640
12,660
Phytomonas HART1 AGTGTACACCAGCCAACACCCACCCCAAACACCCCACCCACCACGCCCAAAACAGCATACCTGTATAAATACACAGTGCACCCCACACCCCACCCA
Translation ORF/CDS
Phytomonas serpens
Translation ORF/CDS

12,680 12,700 12,720 12,740 12,760

Phytomonas HART1 CCCAAAAACAACTACCCCACGAAAAAAAATGTATTTGTAAGATAATACCTATGTGTAAACACATAGTAAAAACGAAAAACACCAGTAAAACTTGCAG
Translation ORF/CDS

Phytomonas serpens -
Translation ORF/CDS

12,780 12,800 12,820 12,840 12,860

Phytomonas HART1 ATAATAAACTAAAGTAAATTTAAAAGTAAAACAAATTAAATTAAAAGTAAATCAAAACAAATTGAAAGTAAATCAAAGTAAATTAAAAGTAAATCA
Translation ORF/CDS

Phytomonas serpens -
Translation ORF/CDS

12,880 12,900 12,920 12,940 12

Phytomonas HART1 AAATAGACTGAAAGTAAATTAAAACAGACTGAAAGTAAAACAAAGTAAATTAAAAGTAAATTAAAATAAATTGAAAGTAAATCAAAACAAATTAAA
Translation ORF/CDS

Phytomonas serpens -
Translation ORF/CDS

,960

Phytomonas HART1 AGTAAA 12099
Translation ORF/CDS

Phytomonas serpens - - - - - - 10478
Translation ORF/CDS
