# Supplementary material for: The Streamlined Genome of Phytomonas spp. Relative to Human Pathogenic Kinetoplastids Reveals a Parasite Tailored for Plants
Source: PLoS Genet. 2014 Feb 6;10(2):e1004007. doi: 10.1371/journal.pgen.1004007 (PMC3916237; doi:10.1371/journal.pgen.1004007)
Supplement: Table S1 — Scaffold somy calls in Phytomonas EM1 and Hart1 isolates. Median read depth coverage was computed for each scaffold across the whole EM1 and HART1 assemblies, and normalized by setting the average of the read depth to 2 (details of the procedure used can be found in Materials and Methods). Scaffolds bigger than 100 kb (scaffolds above the red line) highlighted in yellow are supernumerary. (DOC) [file pgen.1004007.s024.doc]

| **EM1** | | **HART1** | |
| --- | --- | --- | --- |
| **Scaffold number** | **Somy** | **Scaffold number** | **Somy** |
| scaffold_1 | 2,02 | scaffold_1 | 2,16 |
| scaffold_2 | 1,95 | scaffold_2 | 1,99 |
| scaffold_3 | 1,95 | scaffold_3 | 2 |
| scaffold_4 | 2,08 | scaffold_4 | 1,84 |
| scaffold_5 | 2,96 | scaffold_5 | 2,01 |
| scaffold_6 | 2,08 | scaffold_6 | 2,4 |
| scaffold_7 | 1,95 | scaffold_7 | 2,03 |
| scaffold_8 | 1,95 | scaffold_8 | 2,05 |
| scaffold_9 | 1,95 | scaffold_9 | 1,85 |
| scaffold_10 | 1,89 | scaffold_10 | **3,1** |
| scaffold_11 | 2,27 | scaffold_11 | 2 |
| scaffold_12 | 1,89 | scaffold_12 | 2,23 |
| scaffold_13 | 1,83 | scaffold_13 | **4,15** |
| scaffold_14 | 2,83 | scaffold_14 | 2,42 |
| scaffold_15 | 1,7 | scaffold_15 | 2,05 |
| scaffold_16 | 1,95 | scaffold_16 | 2,47 |
| scaffold_17 | 2,08 | scaffold_17 | **3,06** |
| scaffold_18 | 1,51 | scaffold_18 | **3,02** |
| scaffold_19 | 1,95 | scaffold_19 | 2,11 |
| scaffold_20 | 1,45 | scaffold_20 | 2,67 |
| scaffold_21 | 1,95 | scaffold_21 | 1,94 |
| scaffold_22 | 1,89 | scaffold_22 | **6,67** |
| scaffold_23 | 2,2 | scaffold_23 | 1,94 |
| scaffold_24 | **3,65** | scaffold_24 | 1,23 |
| scaffold_25 | **4,03** | scaffold_25 | 3,85 |
| scaffold_26 | 1,89 | scaffold_26 | 1,49 |
| scaffold_27 | 1,89 | scaffold_27 | 1,05 |
| scaffold_28 | 1,89 | scaffold_28 | 4,14 |
| scaffold_29 | 1,57 | scaffold_29 | 4,69 |
| scaffold_30 | 2,08 | scaffold_30 | 5,21 |
| scaffold_31 | 1,64 | scaffold_31 | 4,41 |
| scaffold_32 | 1,89 | scaffold_32 | 4,65 |
| scaffold_33 | 1,7 | scaffold_33 | 1,67 |
| scaffold_34 | 1,76 | scaffold_34 | 2,38 |
| scaffold_35 | 1,95 | scaffold_35 | 1,78 |
| scaffold_36 | 1,7 | scaffold_36 | 4,1 |
| scaffold_37 | 1,7 | scaffold_37 | 0,45 |
| scaffold_38 | 1,57 | scaffold_38 | 1,25 |
| scaffold_39 | 2,14 | scaffold_39 | 29,75 |
| scaffold_40 | 1,32 | scaffold_40 | 0,31 |
| scaffold_41 | 1,7 | scaffold_41 | 2,02 |
| scaffold_42 | **2,77** | scaffold_42 | 2,08 |
| scaffold_43 | 1,26 | scaffold_43 | 3 |
| scaffold_44 | 1,01 | scaffold_44 | 1,97 |
| scaffold_45 | 1,95 | scaffold_45 | 0,63 |
| scaffold_46 | 1,51 | scaffold_46 | 3,22 |
| scaffold_47 | 1,39 | scaffold_47 | 1,09 |
| scaffold_48 | 1,51 | scaffold_48 | 0,79 |
| scaffold_49 | 1,7 | scaffold_49 | 1,54 |
| scaffold_50 | 1,95 | scaffold_50 | 1,98 |
| scaffold_51 | 1,57 | scaffold_51 | 3,65 |
| scaffold_52 | 1,95 | scaffold_52 | 0,96 |
| scaffold_53 | 1,57 | scaffold_53 | 1,37 |
| scaffold_54 | 1,2 | scaffold_54 | 2,57 |
| scaffold_55 | 1,32 | scaffold_55 | 6,04 |
| scaffold_56 | 2,08 | scaffold_56 | 35,7 |
| scaffold_57 | 4,66 | scaffold_57 | 9,45 |
| scaffold_58 | 1,2 | scaffold_58 | 5,74 |
| scaffold_59 | 1,7 | scaffold_59 | 2,24 |
| scaffold_60 | 1,76 | scaffold_60 | 2,07 |
| scaffold_61 | 0,69 | scaffold_61 | 4,45 |
| scaffold_62 | 1,13 | scaffold_62 | 1,98 |
| scaffold_63 | 4,98 | scaffold_63 | 30,57 |
| scaffold_64 | 1,2 | scaffold_64 | 1,02 |
| scaffold_65 | 2,2 | scaffold_65 | 16,22 |
| scaffold_66 | 0,82 | scaffold_66 | 4,22 |
| scaffold_67 | 1,95 | scaffold_67 | 1,25 |
| scaffold_68 | 6,93 | scaffold_68 | 7,6 |
| scaffold_69 | 1,95 | scaffold_69 | 17,9 |
| scaffold_70 | 4,54 | scaffold_70 | 39,13 |
| scaffold_71 | 2,58 | scaffold_71 | 2,1 |
| scaffold_72 | 2,14 | scaffold_72 | 6,51 |
| scaffold_73 | 1,76 | scaffold_73 | 1,11 |
| scaffold_74 | 0,57 | scaffold_74 | 6,55 |
| scaffold_75 | 2,65 | scaffold_75 | 11,86 |
| scaffold_76 | 25,64 | scaffold_76 | 1,11 |
| scaffold_77 | 0,82 | scaffold_77 | 1,33 |
| scaffold_78 | 1,89 | scaffold_78 | 3,94 |
| scaffold_79 | 4,09 | scaffold_79 | 2,34 |
| scaffold_80 | 0 | scaffold_80 | 2,19 |
| scaffold_81 | 0 | scaffold_81 | 3,72 |
| scaffold_82 | 1,83 | scaffold_82 | 8,72 |
| scaffold_83 | 1,07 | scaffold_83 | 1,04 |
| scaffold_84 | 0 | scaffold_84 | 2,89 |
| scaffold_85 | 3,97 |  |  |
| scaffold_86 | 3,97 |  |  |
| scaffold_87 | 2,96 |  |  |
| scaffold_88 | 1,07 |  |  |
| scaffold_89 | 4,98 |  |  |
| scaffold_90 | 0,94 |  |  |
| scaffold_91 | 2,08 |  |  |
| scaffold_92 | 1,83 |  |  |
| scaffold_93 | 0,69 |  |  |
| scaffold_94 | 1,64 |  |  |
| scaffold_95 | 2,96 |  |  |
| scaffold_96 | 1,76 |  |  |
| scaffold_97 | 1,83 |  |  |
| scaffold_98 | 1,32 |  |  |
| scaffold_99 | 1,39 |  |  |
| scaffold_100 | 0,38 |  |  |
| scaffold_101 | 1,2 |  |  |
| scaffold_102 | 3,9 |  |  |
| scaffold_103 | 1,76 |  |  |
| scaffold_104 | 1,76 |  |  |
| scaffold_105 | 1 |  |  |
| scaffold_106 | 1,25 |  |  |
| scaffold_107 | 1,51 |  |  |
| scaffold_108 | 2,07 |  |  |
| scaffold_109 | 0,88 |  |  |
| scaffold_110 | 1,38 |  |  |
| scaffold_111 | 0,81 |  |  |
| scaffold_112 | 7,81 |  |  |
| scaffold_113 | 0,75 |  |  |
| scaffold_114 | 1,76 |  |  |
| scaffold_115 | 1,95 |  |  |
| scaffold_116 | 0,56 |  |  |
| scaffold_117 | 2,01 |  |  |
| scaffold_118 | 1,51 |  |  |
| scaffold_119 | 1,19 |  |  |
| scaffold_120 | 1,25 |  |  |
| scaffold_121 | 1 |  |  |
| scaffold_122 | 1,95 |  |  |
| scaffold_123 | 1,95 |  |  |
| scaffold_124 | 1,95 |  |  |
| scaffold_125 | 1,19 |  |  |
| scaffold_126 | 2,2 |  |  |
| scaffold_127 | 11,52 |  |  |
| scaffold_128 | 0,88 |  |  |
| scaffold_129 | 1,63 |  |  |
| scaffold_130 | 7,62 |  |  |
| scaffold_131 | 1,32 |  |  |
| scaffold_132 | 1,2 |  |  |
| scaffold_133 | 1,45 |  |  |
| scaffold_134 | 1,39 |  |  |
| scaffold_135 | 1,64 |  |  |
| scaffold_136 | 0,88 |  |  |
| scaffold_137 | 1,07 |  |  |
| scaffold_138 | 1,39 |  |  |
