# Supplementary material for: The Streamlined Genome of Phytomonas spp. Relative to Human Pathogenic Kinetoplastids Reveals a Parasite Tailored for Plants
Source: PLoS Genet. 2014 Feb 6;10(2):e1004007. doi: 10.1371/journal.pgen.1004007 (PMC3916237; doi:10.1371/journal.pgen.1004007)
Supplement: Table S2 — Resources used for Phytomonas EM1 and HART1 genome annotation. (DOC) [file pgen.1004007.s025.doc]

|  | *Phytomonas* spp. | |
| --- | --- | --- |
| Description | EM1 | HART1 |
| % of masked bases | 0.6 | 0.7 |
| % of mapped cDNAs 454 reads* (avg.)‏ | 72 | 86 |
| % of mapped cDNAs 454 reads* from the other isolate (avg.)‏ | 10 | 17 |
| % of mapped Kinetoplastid ESTs (avg.)‏ | 15 | 16 |
| % of mapped proteins from (UNIPROT) | 3 | 3 |
| # of *Ab initio* gene models (SNAP) | 6,535 | 6,825 |
