# Supplementary material for: The Streamlined Genome of Phytomonas spp. Relative to Human Pathogenic Kinetoplastids Reveals a Parasite Tailored for Plants
Source: PLoS Genet. 2014 Feb 6;10(2):e1004007. doi: 10.1371/journal.pgen.1004007 (PMC3916237; doi:10.1371/journal.pgen.1004007)
Supplement: Table S7 — Transposable elements in EM1 and HART1 Phytomonas genomes. a, number of amino acids contained in the multifunctional protein encoded by the consensus sequence of autonomous and active retroposons; b, autonomous retroposons (“Auto”) potentially code for a protein responsible for their retrotransposition. Retroposons are considered active when bioinformatics analyses suggest recent retrotransposition events for most of the elements in the family; c, copy number per haploid genome.; d, not determined due to the high sequence heterogeneity; e, non-coding retroposons; f, the copy number of each retroposon in the 41.8 Mb dataset (the size of the haploid genome is not known); g, the copy number of each retroposon in the 47.7 Mb dataset (the size of the haploid genome is not known); h, number of copies in the assembled sequences. (DOC) [file pgen.1004007.s030.doc]

| **Species** | **Name** | **Size**  **(bp)** | **Gene product^a^** | **Autonomous - Active^b^** | **Copy Nb^c^** | **Ref** |
| --- | --- | --- | --- | --- | --- | --- |
| *T. brucei* | Tbingi | 5,250 | 1,657 | Auto - Active | 115 | [62] |
|  | TbDIRE | ~ 5,000 | *nd*^d^ | Auto - | 73 | [64] |
|  | TbRIME | 500 | NC^e^ | - Active | 86 | [62] |
|  | TbSIDER | ~ 570 | NC | - | 22 | [65] |
| *T. congolense* | Tcoingi | 5,404 | 1,751 | Auto - Active | 56^f^ | [66] |
|  | LITco | 4,733 | 1,505 | Auto - Active | 12^f^ | [66] |
|  | TcoDIRE | ~ 5,000 | *nd* | Auto - | 173^f^ | [66] |
| *T. vivax* | Tvingi | 5,419 | 1,752 | Auto – Active | 756^g^ | [66] |
|  | TvDIRE | ~ 5,000 | *Nd* | Auto - | 108^g^ | [66] |
|  | TvRIME | 1,030 | NC | - Active | 58^g^ | [66] |
| *T. cruzi* | L1Tc | 4,736 | 1,524 | Auto - Active | 320 | [63] |
|  | TcDIRE | ~ 5,000 | NC | Auto - | 257 | [64] |
|  | NARTc | 260 | NC | - Active | 133 | [63] |
| *L.major* | LmDIRE | ~ 5,000 | *nd* | Auto - | 52 | [64] |
|  | LmSIDER | ~ 550 | NC | - | 1,858 | [65] |
| *L. braziliensis* | LbDIRE | ~ 5,000 | *nd* | Auto - | 65 | [66] |
|  | LbSIDER | ~ 550 | NC | - | 1,986 | [67] |
| *Phytomonas* EM1 | PhDIRE | ~ 5,000 | *nd* | Auto - ? | 41^h^ |  |
|  | PhSIDER | ~ 680 | NC | - ? | 7^h^ |  |
| *Phytomonas* HART1 | PhDIRE | ~ 5,000 | *nd* | Auto - ? | 2^h^ |  |
