# Supplementary material for: The Streamlined Genome of Phytomonas spp. Relative to Human Pathogenic Kinetoplastids Reveals a Parasite Tailored for Plants
Source: PLoS Genet. 2014 Feb 6;10(2):e1004007. doi: 10.1371/journal.pgen.1004007 (PMC3916237; doi:10.1371/journal.pgen.1004007)
Supplement: Table S8 — Conservation and taxonomic distribution of Phytomonas isolate snoRNAs. (DOC) [file pgen.1004007.s031.doc]

| Comparison | H/ACA snoRNA | C/D snoRNA |
| --- | --- | --- |
| EM1-HART1 | 75% to 90% | 75% to 93% |
| EM1 – *L. major* | 55% to 76% | 43% to 76% |
| EM1 – *T. brucei* | 34% to 76% | 39% to 78% |
| HART1 - *L. major* | 42% to 73% | 37% to 76% |
| HART1 – *T. brucei* | 22% to 78% | 32% to 73% |
