# Supplementary material for: The Streamlined Genome of Phytomonas spp. Relative to Human Pathogenic Kinetoplastids Reveals a Parasite Tailored for Plants
Source: PLoS Genet. 2014 Feb 6;10(2):e1004007. doi: 10.1371/journal.pgen.1004007 (PMC3916237; doi:10.1371/journal.pgen.1004007)
Supplement: Text S1 — Supplementary information. A detailed description of methods used for the sequencing and annotation of Phytomonas EM1 and HART1 genomes and the manual inspection of selected Phytomonas gene families. (DOC) [file pgen.1004007.s037.doc]

**SUPPLEMENTARY INFORMATION**

**TABLE OF CONTENTS:**

**1 Sequencing and assembly**

**2 Genome Annotation**

**2.1 Repeat Masking**

**2.2 Genewise**

**2.3 GeneID and SNAP**

**2.4 *Phytomonas* cDNAs**

**2.5 Kinetoplastids ESTs**

**2.6 Integration of resources using GAZE**

**2.7 Identifying blocs of co-oriented genes**

**3 Manual inspection of *Phytomonas* selected gene families**

**3.1 RNA genes**

**3.2 Small nuclear RNAs (snoRNAs)**

**3.3 Transposable elements (TEs) in *Phytomonas* genomes**

**3.4 *Phytomonas* kinases in EM1 and HART1 isolates**

**3.5 Amino acid and sugar transporters in *Phytomonas* EM1 and HART1**

**3.6 Calcium homeostasis and acidocalcisome function in *Phytomonas* EM1 and HART1**

**3.7 *Phytomonas* EM1 and HART1 metabolism**

**3.7.1 Glycoconjugates**

**3.7.2 Mitochondrial metabolism**

**3.7.3 Lipid metabolism**

**3.7.4 Purine salvage and pyrimidine synthesis**

**3.7.5 Vitamins and cofactors**

**3.7.6 Folate metabolism**

**1 Sequencing and assembly**

The *Phytomonas* EM1 and HART1 genomes were sequenced using a Whole Genome Shotgun strategy. Data was generated using next generation sequencers: Roche/454 GSFLX Titanium and Illumina HiSeq 1000, together with a 0.1X coverage by Sanger reads on an Applied Biosystems xl3070.

The Roche/454 reads were assembled with Newbler, generating 1,454 contigs (contig N50=4.7 kb; European Nucleotide Archive http://www.ebi.ac.uk/ena/data/view/CAVQ010000001-CAVQ010001400 and http://www.ebi.ac.uk/ena/data/view/CAVR010000001-CAVR010002560) linked into 138 scaffolds for EM1. In the case of HART1, sequence reads were assembled in 2,622 contigs (contig N50=12.1 kb) linked into 84 scaffolds. Post-processing of both EM1 and HART1 assemblies was applied to remove bacterial contaminations. Each scaffold was cut into 100 nt overlapping windows of 1 kb, and each window was aligned against the nr databases with BLASTX . The windows were categorized by their best hit in one of the following categories: bacterial, archae, eukaryote or unknown. Scaffolds composed of at least 60% of bacterial windows were considered as bacterial contaminations and were removed.

**2 Genome Annotation**

**2.1 Repeat Masking**

Most of the genome comparisons were performed with repeat masked sequences. For this purpose, we searched and masked sequentially several kinds of repeats:

• known repeats and transposons available in Repbase with the Repeat masker program .

• tandem repeats with the TRF program .

• ab initio repeat detection with RepeatScout . While de novo repeats only covered 0.2% of the EM1 genome, this procedure identified repeated sequences in over 1.5% of the HART1 genome, possibly corresponding to specific TE or gene families only represented in the pathological *Phytomonas* isolate.

From this pipeline, only 0.6-0.7% of the assembled bases were masked.

**2.2 Genewise**

The Uniprot database (11,009,767 sequences) was used to detect conserved genes between *Phytomonas* EM1 and HART1 isolates and other species. As Genewise is time greedy, the Uniprot database was first aligned with both the *Phytomonas* EM1 and HART1 genome assemblies using BLAT . Subsequently, we extracted the genomic regions where no proteic hit had been found by BLAT and realigned Uniprot protein with more permissive parameters. Each significant match was then refined using Genewise in order to identify exon/intron boundaries (Table S2).

**2.3 Geneid and SNAP**

SNAP *ab initio* gene prediction software were trained on open reading frames derived from the *Phytomonas* EM1 and HART1 cDNA contigs.

**2.4 *Phytomonas* cDNAs**

*Phytomonas* full-length cDNA libraries have been constructed. Sequences were generated using 454 GS-FLX pyrosequencing technology, which produced 274,106 useful reads for EM1 isolate (average size 275 nt.), and 279,579 (average size 327 nt.) for HART1 isolate. After a cleaning procedure, these reads, masked for the splice leader gene sequence (Accession Number AF250969 for HART1) were aligned to the corresponding *Phytomonas* genome assembly with the following pipeline: the sequences were aligned with BLAT on the assembly and only the best match (with identity percent greater than 90%) for each read was selected. Then, each match was extended by 1 kb on each end, and realigned with the read using the Est2genome software .

Also, the *Phytomonas* EM1 and HART1 cDNA reads were assembled by Newbler (software release 2.0.00.20) into 2,712 (EM1; average size 808 nt) and 2,654 (HART1; average size 879 nt) contigs. These contigs were mapped on the corresponding genome assembly following the same pipeline used for reads mapping (Table S2).

**2.5 Kinetoplastids ESTs**

A collection of 72,179 public mRNAs from the Kinetoplastids clade (downloaded from the EMBL database) were first aligned with the *Phytomonas* EM1 and HART1 genome assemblies by BLAT , using default parameters between translated genomic and translated ESTs. To refine BLAT alignment, we used Est2Genome . Only the best match (from BLAT alignments) was selected for each mRNAs sequences. Afterwards, Est2Genome software was used in order to refine BLAT alignments (Table S2).

**2.6 Integration of resources using GAZE**

All the resources described above were later used to automatically build *Phytomonas* EM1 and HART1 gene models using GAZE . GAZE was set up to predict only genes without introns.

A weight was assigned to each resource to further reflect its reliability and accuracy in predicting gene models

cDNA reads, transcript start (SL AG) and transcript stop (poly A) > SNAP > Uniprot > ESTs *Phytomonas* and Kinetoplastids

This weight acts as a multiplier for the score of each information source, before processing by GAZE. When applied to the entire assembled sequence, GAZE predicted 8,270 and 8,458 EM1 and HART1 gene models. Finally, gene predictions created by GAZE were filtered following their scores, their lengths and the presence of co-oriented genes, resulting in 6,381 EM1 and 6,451 HART1 gene models (Figure 1A).

**2.7 Identifying blocs of co-oriented genes**

Protein-coding genes in trypanosomes are organized into large polycistronic transcription units (PTUs), comprising tens-to-hundreds copies successively arranged on the same DNA strand . Therefore, clusters of genes with the same orientation and located on the same strand were grouped in blocks, where order of each gene depends on its position in the cluster. Blocks with only one gene with a score smaller than 5 were next filtered. Novel blocks were recreated, resulting in a final set of 298 and 334 gene blocks for *Phytomonas* EM1 and HART1 isolates, respectively (Figure S6).

**3. Manual inspection of *Phytomonas* EM1 and HART1 selected gene families**

**3.1 RNA genes**

Trypanosomatids exhibit other distinctive features, as trans-splicing of precursor RNAs. Even though only one copy of the splice leader RNA gene (SL) was found in the assembly from each isolate, several matches were obtained against the non-assembled reads, suggesting that the SL gene is present as multicopy tandem arrays in *Phytomonas*, as previously observed .

Most of the individual tRNAs detected in these *Phytomonas* isolates corresponded to the ones previously identified in *T. brucei*, *L. major* and *T. cruzi*. In spite of the newly acquired Asn and Ser tRNAs, most of the tRNA genes are syntenic between both *Phytomonas* sequenced isolates. As shown in Figure S7, tRNA genes in HART1 scaffold 4 are syntenic with EM1 scaffold 45 with, as the only difference, the presence of the U3 RNA gene in HART1, not found in EM1. In kinetoplastids U snRNA genes are associated precisely (95-98 bp apart) with oppositely-transcribed tRNA genes, which provide the Box-B promoter elements for both genes. In the case of *Phytomonas*, the HART1 U3 gene is associated with the same isoacceptor tRNA (Lys TTT) as previously found in *L. major*.

Together with the synteny of U3 between *Phytomonas* and *L. major*, the 7SL RNA genes are as well highly conserved in both organisms (see Figure S7 B and C). The 7SL RNA gene also shares transcriptional linkage with a tRNA gene. In HART1, 7SL RNA is associated with the same isoacceptor tRNA (Arg -ACG) as found in *L. major*. No tRNA gene was predicted upstream of the EM1 7SL RNA gene. Some snRNAs are not associated with a tRNA gene but are associated with a functional Box-B element. Further investigation would be required to reveal the presence of an upstream box B in EM1. Either way, the synteny at this locus does not appear to be conserved between HART1 and EM1.

**3.2 Small nuclear RNAs (snoRNAs)**

To date 142 small nucleolar RNAs (snoRNAs) have been identified In *T. brucei*. Still, based on mapping data and estimates on other organisms, the total number is likely to be close to 200. The results of an initial scan of the HART1 and EM1 genomes allowed us to identify quite a reduced number of snoRNAs (Table S8). The HART1 and EM1 *Phytomonas* genomes show higher similarity to each other than to *T. brucei* and *L. major*. Besides, *L. major* and *T.brucei* seemed about equally distant from these two genomes with respect to their snoRNA repertoire. Further work must be done to uncover the rest of the snoRNAs including species specific snoRNAs as well as a targeted search for specific small RNAs such as the SLA1. In addition, a global analysis of genomic cluster of these snoRNAs would be able to answer if these snoRNAs are found in clusters as they are found in *L. major* and in *T. brucei* as well as if the synteny is conserved across the clusters.

The repertoire of 34 H/ACA and 57 C/D snoRNAs sequences from *T. brucei* was extracted from . Several additional studies identified 15 H/ACA and 13 C/D molecules from *T. brucei* . Recently, an RNA-Seq study of small RNAs in *T. brucei* yielded additional snoRNAs bringing the total number of published snoRNAs in *T. brucei* to 142, comprising 63 H/ACAs and 79 C/Ds . Similar data for 40 H/ACA and 63 C/D sequences in *L. major* were taken from . Additional unpublished data from a recent snoRNA sequencing dataset which contained 50 H/ACA and 16 C/D in *L. major* was also used. Thus bringing the total number of snoRNAs in *L. major* to 169, 90 H/ACA and 79 C/D.

Orthologs of the published snoRNAs in *T. brucei* and *L. major* were found using WU-BLAST 2.0 (BLASTN) . Any hit regardless of its e- value was accepted on condition that that the hit covered 90% of the length of the query, and that the target sequence, as well as the boxes, were conserved.

Additional C/D snoRNAs were found by Snoscan followed by WU-BLAST 2.0 (BLASTN) versus the *L. major* and *T. brucei* C/D snoRNAs. Those C/Ds that matched in the target area were accepted.

For the H/ACA snoRNAs, published covariance models of *T. brucei* and *L. major* H/ACA snoRNAs were used as input to Infernal to search versus EM1 and HART1 genomes. Those hits that had a p-value of e-05 or lower and a conserved AGA box were considered orthologs.

A total of 37 C/D and 39 H/ACA snoRNAs were identified in EM1, and a total of 38 C/D and 36 H/ACA snoRNAs were identified in HART1. The H/ACA snoRNAs varied in length from about 57 to 84 bp, and the C/D snoRNAs varied in length from about 62 to 136 bp. Search programs were performed on the EM1 and HART1 sequences independently. The results from these programs were then merged and duplicate snoRNAs were removed.

The degree of conservation of the H/ACA and C/D snoRNAs varies among the different trypanosomatid species. The identity between the orthologous molecule (we considered two H/ACA from different species to be orthologous if they have the same target on the rRNA; we considered two C/D from different species to be orthologous if they have the same target on the rRNA) is shown in Table S8.

**3.3 Transposable elements (TEs) in *Phytomonas* genomes**

Approximately 50 BLASTN/TBLASTP searches were performed on each *Phytomonas* genome assembly using the BLAST-2.2.13 package, with the nucleotide and/or amino acid sequences of all known trypanosomatid TE, telomeric repeats (GGGTTA) and SL-RNA genes as queries. The BLAST outputs were loaded on the top of the *Phytomonas* assemblies using Artemis and the annotated TE were saved in separate files.

The BLAST-Extend-Repraze search algorithm (BER) was used to reconstitute the coding sequence of the degenerate TE (DIRE). This algorithm extends the boundaries of each ORF by 300 bp on both ends and a modified Smith-Waterman alignment is then performed between the proteins, including the translation of the extensions. The extensions allow the examination of all translation frames and past stop codons. To tentatively reconstitute chimeric proteins from the analyzed DIRE, frame shifts were removed manually from the DNA sequences using the BER outputs to precisely determine the frame shift positions. This approach was used to generate a pseudogene for each DIRE encoding a single ingi/L1Tc-like sequence that contains numerous stop codons in most cases, as performed before . The phylogenetic analysis was performed as before . The reverse transcriptase (RT) amino acid domain was aligned using the multiple-alignment software CLUSTAL X, followed by minor manual adjustments using MacClade version 4.06 (Sinauer Associates, Inc.). Phylogenetic trees were generated by the neighbour-joining method as implemented in PAUP version 4.0b10 (Sinauer Associates, Inc.), using the default parameters.

Among the PhDIREs detected only PhSIRE-15800 (EM1) contains the reverse transcriptase domain, which is the best TE amino acid domain used to reconstitute the phylogenetic relationship between TE of the ingi clade. This phylogenetic analysis shows that PhSIRE-15800 belongs to the ingi1 subclade, which contains all the L1Tc-related sequences, L1Tc (*T. cruzi*), L1Tco (*T. congolense*) and most LbrDIRE (*L. braziliensis*) (Figure 2). Actually, all the other PhDIRE are more closely related to L1Tc/L1Tco than to Tbingi/Tcoingi/Tvoingi (data not shown). This observation fit with the recent model proposing that L1Tc-related TE appeared in the trypanosomatid ancestor, while ingi-related TE appears later in the trypanosome branch (Figure 2).

Twenty nine of these PhDIREs, including the longest PhDIRE identified (PhDIRE-15800), contain the 79-bp signature, which is a hallmark of all the trypanosomatid retroposons belonging to the ingi clade characterized so far. The first 74 residues of the PhDIREs (as well as the PhSIDERs, see below) are similar to the 79-bp signature all the other TE of the ingi clade.

The EM1 genome also contained 7 relatively conserved sequences (ranging between 569 and 745 bp), which share the first 200 bp with PhDIRE, 98% identical between PhSIDER and PhDIRE consensus sequences (Table S7). Although conserved, the alignment of the 7 PhSIDER showed a high degree of divergence, suggesting that these sequences have not been mobilised in the recent “geological history” and are probably not functional anymore. We proposed that these sequences derived from PhDIRE by deletion to form a PhDIRE/PhSIDER couple as previously proposed for the potentially active Tbingi/TbRIME , Tvingi/TvRIME and L1Tc/NARTc couples, as well as for the non-functional and extinct LmDIRE/LmSIDER couple .

The relative high degree of divergence between the 7 PhSIDER copies was also observed while comparing the first 200 ntd conserved between PhSIDERs (7 copies) and PhDIREs (29 copies). The consensus sequence, determined from the alignment of all the identified elements of a given family, approximates the element’s original sequence at the time of insertion. For the potentially active NARTc and RIME, a significant fraction of the analysed elements is identical or very close to their consensus sequence (with a median value of 2% and 4% of divergence, respectively), suggestive of recent retrotranposition events. However, the non-functional extinct LmSIDER2 and TbSIDER1 are very divergent, with a respective median value of 22% and 11% of divergence from the consensus sequence. The first 200 nt conserved between and PhDIRE are also very divergent (median value of 11% of divergence from the consensus sequence), indicating that the PhDIRE and PhSIDER elements have lost their ability to retrotranspose in the *Phytomonas* EM1 genome.

During retrotransposition, the retroposon-encoded endonuclease performs two assymetrical single-strand cleavages, leading to a duplication of the residues between both cleavages . The duplicated motif, flanking the newly inserted retroposons, is called Target Site Duplication (TSD). One particularity of the retroposons of the ingi clade is the size conservation of the TSD, which is 12 bp long in the case of Tbingi/TbRIME , Tvingi/TvRIME , Tcoingi , L1Tco , L1Tc/NARTc and LmSIDER . Among the 29 PhDIRE containing the 79-bp signature and the 7 PhSIDER identified, 19 (66%) and 6 (86%), respectively, are flanked by a conserved motif (>65% identity), which resemble vestiges of TSDs. Most of the TSD are 12 bp long (23 out of 25). Only 3 PhDIRE/PhSIDER are flanked by identical 12 bp residues, which reflect the relative high degree of divergence.

We previously observed that retroposons of the ingi clade are preceded by a conserved motif, which is considered as the endonuclease binding-site. Because of the relative conservation of this motif, we concluded that the ingi elements display a relative site-specificity for insertion . The conserved sequence is ingi subfamily specific, as exemplified by the comparison of the L1Tc/L1Tco (ingi1), Tbingi (ingi3) and Tvingi/Tcoingi (ingi6) consensus sequences (Figure 2). To determine the sequence conservation upstream of PhDIRE and PhSIDER, which belong to the ingi1 subclade, we considered all the 25 retroposons flanked by a TSD (flanquing). Both the PhSIDER and PhDIRE retroelements are preceded by the same well-conserved patterns (5’ GAxxatGaxxxTxTATGAxxxxaxxxxxx 3’), which is similar to the conserved sequence upstream of L1Tc (5’ GAxxAxGAxxxTxTATGAxxxxxxxxxxx 3’). The conservation of the upstream pattern between the closely related L1Tc/L1Tco and PhDIRE/PhSIDER retroposons (all beloging to the ingi1 subfamily), suggests that the endonuclease domain of these TE recognize similar target sites for insertion. This observed also confirmed that all the identified TE in the *Phytomonas* genomes belong to the ingi1 subclade, which is considered as the early diverging ingi subfamily also present in *Leishmania* spp. (Figure 2) .

No traces of other trypanosomatid TEs, such as VIPER/SIRE , SLACS/CZAR (site-specific retroposon inserted in SL RNA genes; and TATE (site-specific retroposon inserted in telomeric repeats, only observed in *L. braziliensis* were found in these assemblies.

**3.4 *Phytomonas* kinases in EM1 and Hart 1 isolates**

Candidate protein kinases were identified by searching for all predicted proteins bearing the Interpro protein kinase domain (IPR017442) in *Phytomonas* isolates EM1 and HART1. Protein kinases were placed into Groups and Families as defined by using the Kinome web site (http://kinase.com/human/kinome/) and Kinome and some custom scripts to query and annotate the sequences. Candidate protein kinases were use to perform a BLAST search against the *T. brucei* *brucei* 927 proteome to identify orthologs. *L. major* orthologs of *T. brucei* kinases have previously been identified . Likewise, *L. major* orphan kinases, not present in *T. brucei*, were individually used as query against the *Phytomonas* proteomes to identify additional *Phytomonas* kinase orthologs. Confirmation of the orthologous groups was obtained by generating a phylogenetic tree of the *T. brucei* and *L. major* kinases with the *Phytomonas* EM1 and HART1 protein kinases. To identify cyclins and other kinase accessory proteins, the *T. brucei* brucei 927 and/or *L. major* Friedlin protein sequences were used in a BLAST search against the *Phytomonas* EM1 and HART1 databases. Hits were verified by reciprocal blasting against the NCBI non-redundant database. Detailed results of BLAST searches can be seen in Table S3.

Major regulators are conserved in both *Phytomonas* isolates, as they are in *T. brucei* and *L. major*. As examples, the master cell cycle regulators, CRK3 , WEE1 and aurora kinase (AUK1) ,as well as translational control kinase, eukaryotic initiation factor 2 kinase (EIF2K) , glycogen synthase kinase (GSK3) , 3-phophoinositide-dependent kinase 1 (PDK1) , casein kinases (CK1 and CK2 ) and uncoordinated-51-like kinase 1 (ULK1).

Looking at the AGC family kinases, notably PK50 is absent from the draft kinomes of *Phytomonas* spp., suggesting that these isolates may only have one NDR kinase, PK53 , like *Leishmania*. Similarly, only one MOB protein (MOB1B), a putative NDR kinase activator, was found in EM1; the second MOB1A protein found in *T. brucei* was not identified (Table S3). Further, only one catalytic subunit for PKA has been identified in each of the *Phytomonas* isolates (Table S3) rather than the 3 catalytic subunits found in other organisms including the TriTryps , raising the possibility that PKA comprises a homodimer of the single catalytic subunit with a dimer of the regulatory subunit PKAR, which was also identified in both *Phytomonas* isolates (Table S3).

There are also reduced numbers of Ca2+/calmodulin-dependent kinases (CAMKs) or CAMK-like kinases (CAMKLs) in the *Phytomonas* draft kinomes (Table S3). Within the CMGC group, most of the CRKs are present in both *Phytomonas* isolates, with the exception of CRK10, while the cdc2-like kinases (CLKs) are largely absent (just one in both EM1 and HART1 isolates compared to four in *T. brucei* and *L. major*). There are also reduced numbers of DYRK kinases: five in EM1 (which includes one novel DYRK kinase) and just two in HART1, compared to seven in *T. brucei* and *L. major*) (Table S3). Additionally, only GSK3 short and not GSK3 long [REF49] is present in the *Phytomonas* draft kinomes.

In contrast to the CMGC group, most mitogen activated protein kinases (MAPKs) are conserved in the *Phytomonas* isolates. There are also reduced numbers of NEKs and STE family kinases in the *Phytomonas* isolates compared to the *T. brucei* and L. major counterparts. STE11 kinases are more poorly represented in the *Phytomonas* draft kinomes compared to the *T. brucei* and *L. major* kinomes (Table S3). The function of the unique kinases cannot be predicted from their sequences and further work will be required to define their roles in these *Phytomonas* isolates.

**3.5 Amino acid and sugar transporters in *Phytomonas* EM1 and HART1.**

Parasites amino acid transporters (AAPs) belong to two families; amino acid/auxin permeases (AAAP) and amino acid polyamine organo cation (APC) that contain between 9-14 trans membrane domains (TMDs) with N- termini facing the inside of cells. In the case of human trypanosomes, a specific group of aminoacid transporters was identified . Akerman’s group has named the 25 genes that encode for putative amino acid permeases/transporters in *L. donovani* LdAAP1 through LdAAP25 . These are >95% identical to *L. major* and *L. infantum* AAPs. All LdAAPs show high level of similarity with AAPs from all TriTryps (Table S3) and even share with them specific motifs . Recently, the Zilberstein’s group has identified and characterized the function of three AAPs in *Leishmania donovani*; LdAAP3 encodes an arginine-specific transporter , LdAAP7 encodes a lysine-specific transporter and LdAAP24 a neutral amino acid (proline and alanine) transporter. Functional orthologs for these genes in the trypanosomatids have also been identified; an arginine transporter in *T. cruzi* (TcAAP3 ), neutral amino acid transporters in *T. brucei* and *T. cruzi* (TbAAP24 and TcAAP24, respectively) and lysine transporter in *T. cruzi* (TcAAP7; ).

This time, a remote BLAST search was performed automatically against the non-redundant database (http://www.ncbi.nlm.nih.gov); for each *Phytomonas* protein, using a script developed in-house that uses several BioPerl package modules . We used the term “AAP” (e.g. amino acid permease) since it has already been used to functionally annotate amino acid transporters in all TriTryps, thus (i.e. EM1AAP or HART1AAP). Table S3 E lists all EM1 and HART1 proteins that based on this analyses, are annotated as putative amino acid transporters.

Both isolates contain the same repertoire but different copy numbers of AAPs (except for AAP25, only present in EM1): EM1 genome has two copies of AAP1 whereas HART1 genome contains three copies of this gene. Two of these are adjacent (2,106,001 and 2,108,001) and the third localizes at a distance (7,272,001). Note that the latter ORF contains very short sequence and therefore our prediction has been determined at low confidence. AAP2 appears as a single copy gene in EM1, but as two identical gene copies that are apart from one another in HART1 (3,635,001 and 7,079,001). EM1 genome contain two distantly localized identical copies of AAP20 (5,872,001 and 6,177,001) whereas HART1 has only one gene that encodes for AAP20 (2,177,001). Both species contain two identical copies of AAP21 (Table S3 E). BLAST alignment of the orthologous AAP proteins from the two species indicated high levels of identity (>50%) and expectancy (e values < e-40).

To date, the functions of three AAPs - arginine transporter AAP3 , the lysine transporter AAP7 and the proline-alanine transporter, AAP24 – have been determined experimentally in mammalian trypanosomatids. Their counterparts in the *Phytomonas* genomes have been therefore annotated, at high confidence, as arginine transporters (EM1AAP3 and HART1AAP3), lysine transporters (EM1AAP7 and HART1AAP7) and neutral amino acid transporters HART1(EM1AAP24 and HART1AAP24).

Phylogenetic analysis performed using the amino acid sequences from *Phytomonas* and mammalian trypanosomatids have been performed for the three AAPs whose functions have been determined, AAP3, AAP7 and AAP24. As illustrated in Figure S18, analysis demonstrated that genes clustered in three clades according to their function. i.e. arginine (ARG), lysine (LYS) and proline-alanine (PRO) transporters. This further emphasizes sequence-function association between the trypanosomatid and *Phytomonas* AAP genes in each clade. Hence, we can determine at relatively high confidence that Em1AAP3 and HART1AAP3 are arginine transporters, and that Em1AAP7 and HART1AAP7 are lysine transporters.

Moreover, this analysis indicated how close phylogenetically the *Phytomonas* AAPs are to mammalian trypanosomatid genes. Interestingly, in the ARG clade, Em1AAP3 was closer to Trypanosomes arginine transporter genes whereas HART1AAP3 was closer to *Leishmania* arginine transporters. In contrast, in the other clades both *Phytomonas* genes clustered together and form separate sub-clade, indicating that they are closer to each other than to the other trypanosomatids genes. The analysis in Figure S18 further supported previous observation that trypanosomatids amino acid transporters form a closely related family of AAPs .

So far, the function of three of them has been experimentally verified in mammalian trypanosomes: that is the case for the arginine (AAP3; ), lysine (AAP7; ) and proline-alanine (AAP24) transporters. The equivalents for these transporters were also identified in both EM1 and HART1 isolates. Unexpectedly, phylogenetic analysis of these *Phytomonas* and mammalian trypanosomatid AAPs supported previous observation, showing that trypanosomatid amino acid transporters form a closely related family of AAPs (Figure S18).

It is remarkable to note that, even though *Phytomonas* are plant parasites, the EM1 and HART1 AAPs were phylogenetically closer to human trypanosomatids than to plants. Previous phylogenetic analysis indicated that *Leishmania* AAPs are closer to plants than to human ones, most likely due to processes such as gene transfer . In our case, analysis strongly suggested *Phytomonas* AAP genes as close to plant AAPs as to the trypanosomatid counterparts, suggesting lack or minimal gene transfer from their plant hosts.

Another interesting feature was the closeness of *Phytomonas* lysine and arginine transporters to each other compared with the proline-alanine transporter (PRO) family. Even if AAPs transport lysine and arginine in higher eukaryotes , AAP7 and AAP3 transport lysine and arginine separately in *Leishmania* and *T. cruzi*. This indicates that either ancient eukaryotes transported these amino acids separately and they merged later in evolution or that separation is required for virulence. Our analysis identified 23 EM1 and 24 HART1 putative ABC transporter genes. Using phi-BLAST analysis we were able to define the sub families of about half of the annotated transporters (Table 2 and Table S12). The largest sub-family of ABC transporters in both isolates is ABCA, with six genes in HART1 and seven in EM1. The second group in size is ABCG, with three genes in EM1 and five in HART1. This analysis did not identify ABCC, ANCD, ABCF and ABCH sub-families, even though both are present in mammalian trypanosomatids . Transporters of these sub-families might be present in the list of “unknown” ABC transporters. Interestingly, ABCA is also the largest sab family group of ABC transporters in *Leishmania* but not in *T. cruzi* and *T. brucei*, where distribution is equal across all ABC transporter sub-families.

**3.6 Calcium homeostasis and acidocalcisome function in *Phytomonas* EM1 and HART1**

The BLAT search tool from the SeqTryplant Genome browser was used to find *T. cruzi* orthologous sequences in the *Phytomonas* EM1 and HART1 genomes. The *T. cruzi* protein sequences were provided to do the BLAT search and find orthologs previously found in acidocalcisomes, or demonstrated to be important in regulation of Ca2+ homeostasis including Ca2+-pumps, Ca2+-binding proteins, vacuolar proton ATPase subunits, vacuolar transporter chaperones (VTC), exopolyphosphatase, and soluble and vacuolar-type proton pyrophosphatases. The predicted protein sequences obtained from the BLAT search were confirmed using the BLASTp search tool from the OrthoMCL DB (version 4) resource (www.orthomcl.org) and the *Phytomonas* gene ID were annotated after positive identification. When a region in the genome was found to contain the *T. cruzi* orthologous sequence (query) but a gene ID and annotation was missing, we only included information on the scaffold region. These sequences require confirmation of the transcript and predicted protein sequences for gene ID assignation and annotation. In the case that more than one sequence was found for the same query and the gene ID was missing, the selection of the putative sequence was based on the score, sequence coverage and percent of protein identity. If the query sequence orthologue was not found, we labeled it as “Not identified”.

Trypanosomatids control intracellular Ca2+ using several Ca2+ transporting systems. They apparently lack some of the proteins that control influx of Ca2+ across the plasma membrane of higher eukaryotes. For example, there is no evidence for receptor-operated (Ca2+ influx after receptor stimulation) or store-operated (Ca2+ influx initiated by depletion of intracellular stores) Ca2+ channels. There are no orthologs, in any trypanosomatid genome, including those of the *Phytomonas* EM1 and HART1 isolates, to the proteins STIM (the endoplasmic reticulum Ca2+ sensor) and ORAI (the Ca2+ channel forming subunit), which are involved in store operated Ca2+ entry in higher eukaryotes . A putative voltage-dependent calcium channel was identified in some trypanosomatid genomes, such as that of *T. cruzi* and orthologs are present in *Phytomonas* HART1 and EM1 (Table S3A, calcium pump and channels). The protein product of the *T. brucei* gene is located in the flagellar membrane . Demonstration of these gene products as functional calcium channel awaits direct analysis by electrophysiology. Eukaryotic cells export Ca2+ using either a Na+/Ca2+ exchanger or a Ca2+ ATPase (PMCA). However, Na+/Ca2+ exchangers are not present in early eukaryotes . PMCA-type Ca2+-ATPases, in contrast, were characterized and localized to the plasma membrane and acidocalcisomes of *T. cruzi* and *T. brucei* and both *Phytomonas* spp. possess orthologs to these genes. Gene orthologs to one encoding for another putative PMCA of *T. cruzi* (Tc00.1047053509647.150) were also identified in both *Phytomonas* isolates (Table S3A, calcium pump and channels). There are also gene orthologs to what has described as a Na+-ATPase in *T. cruzi* (ENA) in both *Phytomonas* isolates (Table S3A).

The endoplasmic reticulum also possesses a Ca2+-ATPase (SERCA) for influx and a channel for efflux. Two orthologs to SERCA-type Ca2+-ATPasas of *T. cruzi* (TcSCA1, Tc00.1047053509770.70, ) and *T. brucei* (Tba1, ) are present in both *Phytomonas* isolates. Ca2+ release from the ER of higher eukaryotic cells is mediated by ryanodine (RyR) or inositol 1,4,5-trisphosphate (InsP3R) channels. RyR are activated by a rise in intracellular Ca2+ [Ca2+], (Ca2+-induced Ca2+ release, CICR). In addition, there are RyR-like channels activated by cyclic ADP-ribose (cADPR), sphingosine, and nicotinic acid adenine dinucleotide phosphate (NAADP) . *T. cruzi* phosphoinositide-specific phospholipase C (TcPI-PLC, Tc00.1047053504149.160) -the enzyme that generates the second messengers InsP3 and diacylglycerol- was well characterized in *T. cruzi* and orthologs to either the *T. cruzi* phospholipase C (Tc00.1047053504149.160; Table S3A, Ca-signalling) or the putative *T. cruzi* InsP3 receptor (Tc00.1047053509461.90, Table S3A, calcium pumps and channels) are present in other pathogenic trypanosomatids but are absent in both *Phytomonas* isolates. The *T. brucei* InsP3 receptor was recently found to be functional and localized to acidocalcisomes . Acidocalcisomes of *Trypanosoma brucei* have an inositol 1,4,5-trisphosphatase receptor that is required for growth and infectivity . The intramembrane aspartyl protease presenilin, which is predominantly localized in the ER, may interact with the SERCA ATPase to modulate Ca2+ influx into the ER or affect endogenous leak of channels from the ER . An ortholog of the *T. cruzi* presenilin (Tc00.1047053503543.10) was found in both EM1 and HART1 isolates.

Ca2+ moves into mitochondria first through a voltage-dependent anion-selective channel localized in the outer mitochondrial membrane (VDAC or mitochondrial porin), which has been identified in *T. brucei* and then down an electrochemical gradient through a uniport mechanism localized in the inner mitochondrial membrane whose molecular nature was recently identified in higher eukaryotes . Orthologs to this MCU are found in all trypanosomatids (Tc00.1047053503893.120 in *T. cruzi*) including *Phytomonas* species (Table S3A, calcium pumps and channels). An ortholog of a mitochondrial protein (MICU1) required for Ca2+ uptake in human cells is also present in trypanosomatids (Tc00.1047053511391.210 in *T. cruzi*; GSHART1T00003862001 in HART1). Efflux from mitochondria appears driven by electroneutral exchange of matrix Ca2+ with external Na+ or H+ and a gene encoding a Ca2+/H+ antiporter has recently been identified in humans (Letm1) and *Drosophila* (CG4589) . We found a human Letm1 ortholog in the genomes of *T. cruzi* (Tc00.1047053507951.270) and both *Phytomonas* isolates.

Acidocalcisomes possess an array of cation and proton transporters. Two proton pumps, a vacuolar H+-ATPase (V-H+-ATPase), and a vacuolar H+-pyrophosphatase (V-H+-PPase) localize to acidocalcisomes and are responsible for their acidification . Genes encoding the V-H+-ATPase complex (14 subunits, Table S3A) and the V-H+-PPase (GSHART1T00007214001 in HART1) were found in both *Phytomonas* isolates. Furthermore, putative soluble pyrophosphatases (GSHART1T00005890001 in HART1) and exopolyphosphatases (GSHART1T00001743001 in HART1) were also identified (Table S3A). Polyphosphate synthases (vacuolar transporter chaperones or VTC’s, ) are present in acidocalcisomes of *T. brucei* and *T. cruzi*. However, VTC proteins were not found in these *Phytomonas* isolates.

Inside the cell, Ca2+ interacts with soluble Ca2+-binding proteins or is sequestered within intracellular organelles in complexes with storage proteins or polyphosphate. The trypanosomatid genome projects uncovered a wide variety of Ca2+-binding proteins (as an example for *T. cruzi* see Table S3A), many of which are uncharacterized and share little or no homology with non-kinetoplastid proteins. We found an orthologue of the *T. cruzi* calmodulin (CaM, Tc00.1047053507483.39), a cytosolic Ca2+ receptor, in both EM1 and HART1 isolates. EF-hand domains are lacking in some of these putative calmodulins in *T. cruzi*, and others have 2–5 of these calcium-binding domains. The specific roles of each protein are unclear, but it is likely that they bind calcium with different affinities and modulate regulatory activity. No orthologs of calreticulin, a Ca2+ storage protein found within the endoplasmic reticulum of *T. cruzi* , were identified in these *Phytomonas* isolates. An interesting Ca2+-binding protein in *T. cruzi* is the flagellar Ca2+-binding protein (FCaBP; ). Multiple copies of the gene encoding this protein are present in its genome (Table S3A). This protein is N-myristoylated and palmitoylated and associates with the flagellar membrane in a calcium-dependent manner reminiscent of the recoverin family of calcium-myristoyl switch proteins . The function of this protein remains unknown. No orthologs of flagellar Ca2+-binding proteins were found in *Phytomonas* isolates. However, an ortholog of another calcium-binding protein that has not been studied in detail in *T. cruzi* was found in *Phytomonas* isolates (Table S3A; calcium-binding proteins).

Two main Ca2+-sensitive proteins that decode Ca2+ signals are protein kinase C (PKC) and Ca2+/calmodulin-dependent kinase (CaMK). Although a group of AGC kinases was identified in the trypanosomatid genomes, it was not possible to assign them to the PKC family by sequence alone . We found several genes encoding putative Ca2+/CaM regulated kinases in the *Phytomonas* EM1 and HART1 genomes (Table S3A). A Ca2+/CaM kinase activity was previously detected in *T. cruzi* , and the soluble enzyme was partially purified and characterized . Ca2+ also activates ion channels and orthologs of *T. cruzi* genes encoding Ca2+-activated K+ channels are present in *Phytomonas* spp. (Table S3A). In addition, orthologs of calcineurin B (GSHART1T00007958001), *T. cruzi* caltractin and several centrins were also identified in both isolates (Table S3A, Ca-signalling).

**3.7 *Phytomonas* metabolism**

The complete proteomes of *Phytomonas* EM1 and HART1 isolates were used in a BLAST search for identification against the SwissProt database with a cut-off value E=1e-^20^. 600 previously characterized metabolic protein sequences of *L. major* were used as query against the complete proteomes of *Phytomonas* EM1 and HART1 isolates. Best hits of both EM1 and HART1 were retained and their protein sequences aligned with the *L. major* sequence. The results obtained using this approach allowed us to build up a database of *Phytomonas* metabolic proteins for both the EM1 and HART1 isolates (Figure 7, Figure 8, Figure S20 and Figure S21; for details see Table S3).

**3.7.1 Glycoconjugates**

*Phytomonas* EM1 and HART1 isolates seem to differ in the make-up of their surface glycoproteins. For example chitin, a surface polysaccharide of N-acetylglucosamine, that was previously identified in *Phytomonas* *françai*  should also be present in the *Phytomonas* EM1 isolate, but apparently cannot be synthesized by the HART1 isolate. (Table S3). Sialic acid cannot be synthesized by any trypanosomatid, including *Phytomonas*. *T. cruzi* and *T. brucei* are able to transfer sialic acid residues from host sialoglycoconjugates to their own surface oligosaccharides, using a unique cell-surface transsialidase. Leishmania has no transsialidase and thus sialic acid is not part of the surface coat. Also *Phytomonas* lacks a transsialidase gene and thus it is unlikely that the latter organism is able to incorporate sialic acid in its surface components. Results are summarized in Figure S20; for details see Table S3.

**3.7.2 Mitochondrial metabolism**

In *Phytomonas* isolates the typical catabolic NAD-dependent isocitrate dehydrogenase isoenzyme of the TCA cycle is absent and has been replaced by an anabolic NADP-dependent isoenzyme. The latter enzyme functions preferably in the reverse direction to generate NADPH required for protection against oxidant stress. As a consequence, it would be difficult to envisage how *Phytomonas* could use a functional TCA cycle for the full oxidation of pyruvate, fatty acids and amino acids into carbon dioxide and water. Instead, the enzymes of the “cycle” are used to supply the cell with the necessary intermediates for various biosynthetic pathways and its metabolism can best be described as a form of aerobic fermentation.

Fully functional mitochondria, as present in most aerobic eukaryotes, generate an electrochemical gradient across the mitochondrial inner membrane by the transfer of electrons from NADH to oxygen via the electron transport chain which comprises the complexes I (NADH dehydrogenase), III (cytochrome bc1), IV (cytochrome oxidase) and V (the mitochondrial ATP synthase). The subunits of these complexes are partly encoded by the mitochondrial genome and partly by the nuclear genome. The HART1 mitochondrial genome encodes the following subunits of complex I: ND1, ND3, ND4, ND5, ND7, ND8 and ND9. In addition, 12 nuclear encoded subunits of the NADH dehydrogenase complex I, also described for *Leishmania* and *Trypanosoma* spp. , were identified (see Table S3). However, several membrane subunits of complex I, believed to be involved in the creation of a proton gradient over the mitochondrial inner membrane seem to be missing. This suggests that the *Phytomonas* complex I would not be capable to generate a membrane potential. Succinate dehydrogenase, or complex II, is also present. Both its flavin and iron sulfur subunits were detected in the nuclear genome, as well as a number of the additional complex II subunits recently identified in *T. cruzi* . Not only electrons coming from NADH and succinate reduce ubiquinone, other mitochondrial dehydrogenases also donate their electrons to ubiquinone. These include an alternative rotenone-insensitive NADH dehydrogenase, a FAD-dependent glycerol-3-phosphate dehydrogenase, involved in the reoxidation of cytosolic NADH via the dihydroxyacetone-phosphate:glycerol-3-phosphate shuttle, and a delta-1-pyrroline-5-carboxylate dehydrogenase, which receives its reducing equivalents directly from proline. However, an electron transfer flavoprotein (ETF)-ubiquinone oxidoreductase, and long, medium and short/branched chain fatty acid-specific mitochondrial acyl-CoA dehydrogenases, which all donate their electrons to the ETF, are absent and this indicates that *Phytomonas* is not capable of oxidizing any fatty acids via a beta oxidation pathway. Moreover, contrary to the situation in the mitochondria of most other eukaryotes, the re-oxidation of reduced ubiquinone cannot be carried out by complex III (cytochrome bc1) since none of its subunits were detected. Cytochrome c, nor any of the subunits of complex IV (cytochrome c oxidase), were detected either. Thus the respiratory components in the *Phytomonas*' mitochondrion are not only not capable of generating a proton gradient over the mitochondrial membrane, but the ubiquinone reduced by the complexes I and II and the above mentioned dehydrogenases can only be oxidized by an alternative oxidase, that transfers electrons from ubiquinol directly to oxygen, similar to what has been described for the bloodstream forms of the African trypanosomes . A mitochondrial ATP synthase is present. Genes encoding the alpha, beta, gamma, delta and epsilon subunits of its catalytic F1 portion were detected in the nuclear genome, while the ATPase 6 subunit was identified on the mitochondrial maxicircle. This indicates that *Phytomonas* generates a mitochondrial membrane potential by the import and subsequent hydrolysis of cytosolic ATP whereby the mitochondrial ATP synthase functions in the reverse direction, similar as has been described earlier for the bloodstream form of the African trypanosome . On the basis of genome analysis, *Phytomonas* possesses a number of mitochondrial solute transporters. For both pyruvate, the end-product of glycolysis, and phosphate there are specific mitochondrial carriers. Some of the carriers seem to be homologues of the dicarboxylate carriers described in other organisms. Also a tri-carboxylate exchanger was identified. These carriers are probably involved in transport of TCA cycle intermediates and of aspartate and glutamate across the mitochondrial membrane. An ATP/ADP exchanger, a possible folate carrier and a mitochondrial ornithine carrier were also found.

**3.7.3 Lipid metabolism**

The *Phytomonas* genome contains several lipase genes that allow the convertion triglycerides into glycerol and free fatty acids. The glycerol may then serve as an energy substrate through oxidation via the glycolytic pathway. By contrast, the liberated fatty acids can be activated for modification and elongation, but they cannot be used as energy substrates because both *Phytomonas* isolates appear to be unable to oxidize fatty acids via the beta-oxidation pathway. While each isolate has tandemly linked fatty acyl-CoA synthetase genes for the activation of fatty acid for metabolism, the corresponding four acyl-CoA dehydrogenases, each with a different chain-length specificity, as found in *Leishmania* and trypanosomes , are absent in *Phytomonas* (Table S3). Also a glycosomal multifunctional enzyme, a thiolase, a fatty acyl-CoA oxidase homologue and ETF, all essential proteins of peroxisomal beta-oxidation, were not found. The presence of a mitochondrial thiolase gene and genes for respectively a 3,2-trans-enoyl-CoA isomerase and 2,4 dienoyl-CoA reductase, suggest that *Phytomonas* is able to modify unsaturated fatty acids, though. *Phytomonas* should be capable of fatty acid biosynthesis. Several genes for enzymes of type II fatty acid synthesis were identified. They probably serve the synthesis of lipoic acid, an essentiel component of the mitochondrial pyruvate dehydrogenase and 2-ketoacid dehydrogenase complexes. In agreement with observations in the other trypanosomatids, no evidence was found for type I fatty acid biosynthesis. On the other hand the presence of an entire family of tandemly linked fatty acid elongase genes suggests that *Phytomonas* is using the same unique elongation machinery for the synthesis of its fatty acids, as do the other trypanosomatids .

**3.7.4 Purine salvage and pyrimidine synthesis**

Trypanosomatids are unable to form purines de novo (reviewed in ). This notion can be extended to the plant trypanosomes as well. Only the adenylosuccinate lyase gene, one of the 10 genes required to synthesize inosine monophosphate (IMP) from phosphoribosyl pyrophosphate, was identified. However, this lyase also plays a role in purine salvage by converting IMP to AMP and simultaneously aspartate to fumarate in the purine-nucleotide cycle. Most genes required for the interconversion of purine bases and nucleosides were identified. As is the situation in *Trypanosoma* spp. several of their gene products carry a PTS, which suggests that the *Phytomonas* glycosome plays an important role in purine salvage. In the HART1 isolate evidence was found for five genes involved in the de novo synthesis of pyrimidines from glutamine, bicarbonate and aspartate. This situation is identical to that found in the other trypanosomatids (Table S3).

**3.7.5 Vitamins and cofactors**

*Phytomonas* is dependent on a number of exogenous cofactors and / or vitamins as it is not capable of forming thiamine (vitamin B1), biotin, vitamin B12 (no enzymes found at a detection level of 1e-5). However, nicotinamide can most likely be converted to NAD and NADP and coenzyme A can be formed from pantothenic acid. Pyridoxin (vitamin B6) is converted to pyrydoxal-phosphate and riboflavin (vitamin B2) to flavin mononucleotide. While *L. major* and *T. cruzi* both have an ascorbate-dependent peroxidase and a gluconolactone oxidase, enzymes responsible for the synthesis and metabolism of ascorbic acid, homologues of these enzymes were not found in *Phytomonas*. This suggests that *Phytomonas* is unable to form ascorbic acid, and that it relies on the supply of vitamin C from its host.

Haem is required for the synthesis of iron-containing proteins, such as cytochromes and catalase. However, in the two *Phytomonas* isolates sequenced here, neither genes coding for cytochromes nor for catalase were found. Thus it is not surprising that none of the genes coding for the enzymes of the haem biosynthetic pathway were found either. Only a ferrochelatase gene, required for the insertion of the Fe2+ ion into preformed haem, was detected. Details in Table S3.

**3.7.6 Folate metabolism**

Like the other trypanosomatids *Phytomonas* cannot synthesize folates (e.g. folate and biopterin). Genes that encode enzymes of folate biosynthesis are absent and therefore it must import these metabolites from an exogenous source. The HART1 genome counts at least 10 folate and/or pteridine transporter genes while the EM1 genome also has several of them (Table S3). The enzymes required for the reduction of folate to tetrahydrofolate (THF) and those involved in one-carbon (C1) transfer reactions, were all identified .

**Addittional references**

129. States DJ, Gish W (1994) Combined use of sequence similarity and codon bias for coding region identification. J Comput Biol 1: 39-50.

130. Chen N (2004) Using RepeatMasker to identify repetitive elements in genomic sequences. Curr Protoc Bioinformatics Chapter 4: Unit 4 10.

131. Benson G (1999) Tandem repeats finder: a program to analyze DNA sequences. Nucleic Acids Res 27: 573-580.

132. Price AL, Jones NC, Pevzner PA (2005) De novo identification of repeat families in large genomes. Bioinformatics 21 Suppl 1: i351-358.

133. Bairoch A., Apweiler R., Wu C.H., Barker W.C., Boeckmann B., et al. (2005) The Universal Protein Resource (UniProt). Nucleic Acids Res 33: D154-159.

134. Korf I (2004) Gene finding in novel genomes. BMC Bioinformatics 5: 59.

135. Mott R (1997) EST_GENOME: a program to align spliced DNA sequences to unspliced genomic DNA. Comput Appl Biosci 13: 477-478.

136. Liang XH, Uliel S, Hury A, Barth S, Doniger T, et al. (2005) A genome-wide analysis of C/D and H/ACA-like small nucleolar RNAs in Trypanosoma brucei reveals a trypanosome-specific pattern of rRNA modification. RNA 11: 619-645.

137. Myslyuk I, Doniger T, Horesh Y, Hury A, Hoffer R, et al. (2008) Psiscan: a computational approach to identify H/ACA-like and AGA-like non-coding RNA in trypanosomatid genomes. BMC Bioinformatics 9: 471.

138. Barth S, Shalem B, Hury A, Tkacz ID, Liang XH, et al. (2008) Elucidating the role of C/D snoRNA in rRNA processing and modification in Trypanosoma brucei. Eukaryot Cell 7: 86-101.

139. Doniger T, Katz R, Wachtel C, Michaeli S, Unger R (2010) A comparative genome-wide study of ncRNAs in trypanosomatids. BMC Genomics 11: 615.

140. Michaeli S, Doniger T, Gupta SK, Wurtzel O, Romano M, et al. (2012) RNA-seq analysis of small RNPs in Trypanosoma brucei reveals a rich repertoire of non-coding RNAs. Nucleic Acids Res 40: 1282-1298.

141. Liang XH, Hury A, Hoze E, Uliel S, Myslyuk I, et al. (2007) Genome-wide analysis of C/D and H/ACA-like small nucleolar RNAs in Leishmania major indicates conservation among trypanosomatids in the repertoire and in their rRNA targets. Eukaryot Cell 6: 361-377.

142. Lopez R, Silventoinen V, Robinson S, Kibria A, Gish W (2003) WU-Blast2 server at the European Bioinformatics Institute. Nucleic Acids Res 31: 3795-3798.

143. Lowe TM, Eddy SR (1999) A computational screen for methylation guide snoRNAs in yeast. Science 283: 1168-1171.

144. Doniger T, Michaeli S, Unger R (2009) Families of H/ACA ncRNA molecules in trypanosomatids. RNA Biol 6: 370-374.

145. Nawrocki EP, Kolbe DL, Eddy SR (2009) Infernal 1.0: inference of RNA alignments. Bioinformatics 25: 1335-1337.

146. Rutherford K, Parkhill J, Crook J, Horsnell T, Rice P, et al. (2000) Artemis: sequence visualization and annotation. Bioinformatics 16: 944-945.

147. Smith TF, Waterman MS (1981) Identification of common molecular subsequences. J Mol Biol 147: 195-197.

148. Luan DD, Korman MH, Jakubczak JL, Eickbush TH (1993) Reverse transcription of R2Bm RNA is primed by a nick at the chromosomal target site: a mechanism for non-LTR retrotransposition. Cell 72: 595-605.

149. Vazquez M, Ben-Dov C, Lorenzi H, Moore T, Schijman A, et al. (2000) The short interspersed repetitive element of Trypanosoma cruzi, SIRE, is part of VIPER, an unusual retroelement related to long terminal repeat retrotransposons. Proc Natl Acad Sci U S A 97: 2128-2133.

150. Aksoy S, Lalor TM, Martin J, Van der Ploeg LH, Richards FF (1987) Multiple copies of a retroposon interrupt spliced leader RNA genes in the African trypanosome, Trypanosoma gambiense. EMBO J 6: 3819-3826.

151. Villanueva MS, Williams SP, Beard CB, Richards FF, Aksoy S (1991) A new member of a family of site-specific retrotransposons is present in the spliced leader RNA genes of Trypanosoma cruzi. Mol Cell Biol 11: 6139-6148.

152. Manning G, Whyte DB, Martinez R, Hunter T, Sudarsanam S (2002) The protein kinase complement of the human genome. Science 298: 1912-1934.

153. Miranda-Saavedra D, Barton GJ (2007) Classification and functional annotation of eukaryotic protein kinases. Proteins: Structure, Function and Bioinformatics 68: 893-914.

154. Martin DM, Miranda-Saavedra D, Barton GJ (2009) Kinomer v. 1.0: a database of systematically classified eukaryotic protein kinases. Nucleic Acids Res 37: D244-250.

155. Hassan P, Fergusson D, Grant KM, Mottram JC (2001) The CRK3 protein kinase is essential for cell cycle progression of Leishmania mexicana. Mol Biochem Parasitol 113: 189-198.

156. Nurse P, Thuriaux P (1980) Regulatory genes controlling mitosis in the fission yeast Schizosaccharomyces pombe. Genetics 96: 627-637.

157. Li Z, Wang CC (2006) Changing roles of aurora-B kinase in two life cycle stages of Trypanosoma brucei. Eukaryot Cell 5: 1026-1035.

158. Moraes MC, Jesus TC, Hashimoto NN, Dey M, Schwartz KJ, et al. (2007) Novel membrane-bound eIF2alpha kinase in the flagellar pocket of Trypanosoma brucei. Eukaryot Cell 6: 1979-1991.

159. Chow C, Cloutier S, Dumas C, Chou MN, Papadopoulou B (2011) Promastigote to amastigote differentiation of Leishmania is markedly delayed in the absence of PERK eIF2alpha kinase-dependent eIF2alpha phosphorylation. Cell Microbiol 13: 1059-1077.

160. Ojo KK, Gillespie JR, Riechers AJ, Napuli AJ, Verlinde CL, et al. (2008) Glycogen synthase kinase 3 is a potential drug target for African trypanosomiasis therapy. Antimicrob Agents Chemother 52: 3710-3717.

161. Bayascas JR (2010) PDK1: the major transducer of PI 3-kinase actions. Curr Top Microbiol Immunol 346: 9-29.

162. Jensen BC, Kifer CT, Brekken DL, Randall AC, Wang Q, et al. (2007) Characterization of protein kinase CK2 from Trypanosoma brucei. Mol Biochem Parasitol 151: 28-40.

163. Urbaniak MD (2009) Casein kinase 1 isoform 2 is essential for bloodstream form Trypanosoma brucei. Mol Biochem Parasitol 166: 183-185.

164. Garcia-Salcedo JA, Nolan DP, Gijon P, Gomez-Rodriguez J, Pays E (2002) A protein kinase specifically associated with proliferative forms of Trypanosoma brucei is functionally related to a yeast kinase involved in the co-ordination of cell shape and division. Mol Microbiol 45: 307-319.

165. Ma J, Benz C, Grimaldi R, Stockdale C, Wyatt P, et al. (2010) Nuclear DBF-2-related kinases are essential regulators of cytokinesis in bloodstream stage Trypanosoma brucei. J Biol Chem 285: 15356-15368.

166. Hammarton TC, Kramer S, Tetley L, Boshart M, Mottram JC (2007) Trypanosoma brucei Polo-like kinase is essential for basal body duplication, kDNA segregation and cytokinesis. Mol Microbiol 65: 1229-1248.

167. Shaked-Mishan P, Suter-Grotemeyer M, Yoel-Almagor T, Holland N, Zilberstein D, et al. (2006) A novel high-affinity arginine transporter from the human parasitic protozoan Leishmania donovani. Mol Microbiol 60: 30-38.

168. Inbar E, Canepa GE, Carrillo C, Glaser F, Suter Grotemeyer M, et al. (2012) Lysine transporters in human trypanosomatid pathogens. Amino Acids 42: 347-360.

169. Carrillo C, Canepa GE, Giacometti A, Bouvier LA, Miranda MR, et al. (2010) Trypanosoma cruzi amino acid transporter TcAAAP411 mediates arginine uptake in yeasts. FEMS Microbiol Lett 306: 97-102.

170. Stajich JE, Block D, Boulez K, Brenner SE, Chervitz SA, et al. (2002) The Bioperl toolkit: Perl modules for the life sciences. Genome Res 12: 1611-1618.

171. Hatzoglou M, Fernandez J, Yaman I, Closs E (2004) Regulation of cationic amino acid transport: the story of the CAT-1 transporter. Annu Rev Nutr 24: 377-399.

172. Zhang Z, Schaffer AA, Miller W, Madden TL, Lipman DJ, et al. (1998) Protein sequence similarity searches using patterns as seeds. Nucleic Acids Res 26: 3986-3990.

173. Sauvage V, Aubert D, Escotte-Binet S, Villena I (2009) The role of ATP-binding cassette (ABC) proteins in protozoan parasites. Mol Biochem Parasitol 167: 81-94.

174. Cahalan MD (2009) STIMulating store-operated Ca(2+) entry. Nat Cell Biol 11: 669-677.

175. Oberholzer M, Langousis G, Nguyen HT, Saada EA, Shimogawa MM, et al. (2011) Independent analysis of the flagellum surface and matrix proteomes provides insight into flagellum signaling in mammalian-infectious Trypanosoma brucei. Mol Cell Proteomics 10: M111 010538.

176. Pozos TC, Sekler I, Cyert MS (1996) The product of HUM1, a novel yeast gene, is required for vacuolar Ca2+/H+ exchange and is related to mammalian Na+/Ca2+ exchangers. Mol Cell Biol 16: 3730-3741.

177. Lu HG, Zhong L, de Souza W, Benchimol M, Moreno S, et al. (1998) Ca2+ content and expression of an acidocalcisomal calcium pump are elevated in intracellular forms of Trypanosoma cruzi. Mol Cell Biol 18: 2309-2323.

178. Luo S, Rohloff P, Cox J, Uyemura SA, Docampo R (2004) Trypanosoma brucei plasma membrane-type Ca(2+)-ATPase 1 (TbPMC1) and 2 (TbPMC2) genes encode functional Ca(2+)-ATPases localized to the acidocalcisomes and plasma membrane, and essential for Ca(2+) homeostasis and growth. J Biol Chem 279: 14427-14439.

179. Iizumi K, Mikami Y, Hashimoto M, Nara T, Hara Y, et al. (2006) Molecular cloning and characterization of ouabain-insensitive Na(+)-ATPase in the parasitic protist, Trypanosoma cruzi. Biochim Biophys Acta 1758: 738-746.

180. Nolan DP, Reverlard P, Pays E (1994) Overexpression and characterization of a gene for a Ca(2+)-ATPase of the endoplasmic reticulum in Trypanosoma brucei. J Biol Chem 269: 26045-26051.

181. Furuya T, Kashuba C, Docampo R, Moreno SN (2000) A novel phosphatidylinositol-phospholipase C of Trypanosoma cruzi that is lipid modified and activated during trypomastigote to amastigote differentiation. J Biol Chem 275: 6428-6438.

182. Okura M, Fang J, Salto ML, Singer RS, Docampo R, et al. (2005) A lipid-modified phosphoinositide-specific phospholipase C (TcPI-PLC) is involved in differentiation of trypomastigotes to amastigotes of Trypanosoma cruzi. J Biol Chem 280: 16235-16243.

183. Huang G, Bartlett PJ, Thomas AP, Moreno SN, Docampo R (2013) Acidocalcisomes of Trypanosoma brucei have an inositol 1,4,5-trisphosphate receptor that is required for growth and infectivity. Proc Natl Acad Sci U S A 110: 1887-1892.

184. Green KN, LaFerla FM (2008) Linking calcium to Abeta and Alzheimer's disease. Neuron 59: 190-194.

185. Singha UK, Sharma S, Chaudhuri M (2009) Downregulation of mitochondrial porin inhibits cell growth and alters respiratory phenotype in Trypanosoma brucei. Eukaryot Cell 8: 1418-1428.

186. Baughman JM, Perocchi F, Girgis HS, Plovanich M, Belcher-Timme CA, et al. (2011) Integrative genomics identifies MCU as an essential component of the mitochondrial calcium uniporter. Nature 476: 341-345.

187. De Stefani D, Raffaello A, Teardo E, Szabo I, Rizzuto R (2011) A forty-kilodalton protein of the inner membrane is the mitochondrial calcium uniporter. Nature 476: 336-340.

188. Perocchi F, Gohil VM, Girgis HS, Bao XR, McCombs JE, et al. (2010) MICU1 encodes a mitochondrial EF hand protein required for Ca(2+) uptake. Nature 467: 291-296.

189. Jiang D, Zhao L, Clapham DE (2009) Genome-wide RNAi screen identifies Letm1 as a mitochondrial Ca2+/H+ antiporter. Science 326: 144-147.

190. Docampo R, de Souza W, Miranda K, Rohloff P, Moreno SN (2005) Acidocalcisomes - conserved from bacteria to man. Nat Rev Microbiol 3: 251-261.

191. Hothorn M, Neumann H, Lenherr ED, Wehner M, Rybin V, et al. (2009) Catalytic core of a membrane-associated eukaryotic polyphosphate polymerase. Science 324: 513-516.

192. Fang J, Rohloff P, Miranda K, Docampo R (2007) Ablation of a small transmembrane protein of Trypanosoma brucei (TbVTC1) involved in the synthesis of polyphosphate alters acidocalcisome biogenesis and function, and leads to a cytokinesis defect. Biochem J 407: 161-170.

193. Engman DM, Krause KH, Blumin JH, Kim KS, Kirchhoff LV, et al. (1989) A novel flagellar Ca2+-binding protein in trypanosomes. J Biol Chem 264: 18627-18631.

194. Godsel LM, Engman DM (1999) Flagellar protein localization mediated by a calcium-myristoyl/palmitoyl switch mechanism. EMBO J 18: 2057-2065.

195. Ogueta SB, Solari A, Tellez-Inon MT (1994) Trypanosoma cruzi epimastigote forms possess a Ca(2+)-calmodulin dependent protein kinase. FEBS Lett 337: 293-297.

196. Ogueta SB, Macintosh GC, Tellez-Inon MT (1998) Stage-specific substrate phosphorylation by a Ca2+/calmodulin-dependent protein kinase in Trypanosoma cruzi. J Eukaryot Microbiol 45: 392-396.

197. Nakamura CV, Esteves MJ, Andrade AF, Alviano CS, de Souza W, et al. (1993) Chitin: a cell-surface component of Phytomonas francai. Parasitol Res 79: 523-526.

198. Opperdoes FR, Michels PA (2008) Complex I of Trypanosomatidae: does it exist? Trends Parasitol 24: 310-317.

199. Morales J, Mogi T, Mineki S, Takashima E, Mineki R, et al. (2009) Novel mitochondrial complex II isolated from Trypanosoma cruzi is composed of 12 peptides including a heterodimeric Ip subunit. J Biol Chem 284: 7255-7263.

200. Chaudhuri M, Hill GC (1996) Cloning, sequencing, and functional activity of the Trypanosoma brucei brucei alternative oxidase. Mol Biochem Parasitol 83: 125-129.

201. Schnaufer A, Clark-Walker GD, Steinberg AG, Stuart K (2005) The F1-ATP synthase complex in bloodstream stage trypanosomes has an unusual and essential function. EMBO J 24: 4029-4040.

202. Jiang DW, Englund PT (2001) Four Trypanosoma brucei fatty acyl-CoA synthetases: fatty acid specificity of the recombinant proteins. Biochem J 358: 757-761.

203. Stephens JL, Lee SH, Paul KS, Englund PT (2007) Mitochondrial fatty acid synthesis in Trypanosoma brucei. J Biol Chem 282: 4427-4436.

204. Marr JJ, Berens RL (1983) Pyrazolopyrimidine metabolism in the pathogenic trypanosomatidae. Mol Biochem Parasitol 7: 339-356.

205. Vertommen D, Van Roy J, Szikora JP, Rider MH, Michels PA, et al. (2008) Differential expression of glycosomal and mitochondrial proteins in the two major life-cycle stages of Trypanosoma brucei. Mol Biochem Parasitol 158: 189-201.

206. Opperdoes FR, Coombs GH (2007) Metabolism of Leishmania: proven and predicted. Trends Parasitol 23: 149-158.
